# Taxonomic Revision of Hispaniola Tiger Beetles in the Genus *Brasiella* Rivalier 1954 (Coleoptera, Carabidae, Cicindelinae)

**DOI:** 10.3897/zookeys.147.2012

**Published:** 2011-11-16

**Authors:** Robert E. Acciavatti

**Affiliations:** 1 Research Associate, Section of Invertebrate Zoology, The Carnegie Museum of Natural History, 4400 Forbes Avenue, Pittsburgh, Pennsylvania 15213-4080, USA

**Keywords:** Hispaniola, Tiger Beetles, *Brasiella*, new species

## Abstract

The *Brasiella* tiger beetle fauna on Hispaniola, the second largest island of the Greater Antilles, has more species diversity than currently recognized as all populations previously have been assigned to the insular endemic *Brasiella dominicana* (Mandl). A comparative study of adult morphology, particularly male genitalic and female abdominal characters, for available *Brasiella* specimens from populations on Hispaniola, proposes eight additional new species also endemic to this island. Except for three sympatric species in the Sierra de Baoruco in southern Dominican Republic occurring in different habitats, all the *Brasiella* on Hispaniola appear to be allopatric. Most species occur in the major mountainous regions of Hispaniola. Two species, however, are known only from river floodplains in the southern coastal plain of the Dominican Republic. *Brasiella dominicana* (Mandl) and *Brasiella ocoa*, new species, occur along river floodplains emanating from the eastern end of the Cordillera Central in the Dominican Republic. Two new *Brasiella* species, *Brasiella bellorum*, and *Brasiella philipi*, occur in the Cordillera Central, Dominican Republic, the former species from central portions, and the latter species from north slopes of this mountain range, respectively. Three new *Brasiella* species, *Brasiella rawlinsi*, *Brasiella iviei*, and *Brasiella youngi*, are isolated in the Sierra de Baoruco, Dominican Republic, where each occupies a different habitat along an altitudinal gradient. The two new *Brasiella* species in Haiti are *Brasiella darlingtoniana*, in the Massif de la Selle, and *Brasiella davidsoni*, in the Massif de la Hotte. All nine *Brasiella* species on Hispaniola, along with *Brasiella viridicollis* (Dejean) and its two subspecies on Cuba, belong to the *viridicollis* species group of the genus *Brasiella* based on criteria presented in earlier published phylogenetic studies of Brazilian and West Indian tiger beetles. The subspecies *Brasiella viridicollis fernandozayasi* (Kippenhan, Ivie and Hopp) may represent a distinct species within this species group, whereas removal of *Brasiella wickhami* (W. Horn) from this species group seems warranted based on evidence presented. A general overview of species relationships for the *Brasiella* on Hispaniola are discussed, along with the current and ancestral geographic distributions of the *Brasiella viridicollis* species group in the West Indies.

## Introduction

A suite of synapomorphic characters, especially of the male genitalia, not found in related Western Hemisphere Cicindelinae taxa, firmly establishes the distinctiveness of the species classified as *Brasiella* Rivalier, 1954 (Rivalier 1954, 1955). This mostly Neotropical taxonomic grouping of small (<7.5 mm), primarily cursorial, tiger beetles received generic status in the latest and most comprehensive treatises of the Geadephaga of the world (Lorenz 2005), and of the Western Hemisphere Carabidoidea (Erwin and Pearson 2008).

Prior to this revision, all populations on Hispaniola had been assigned to the insular endemic *Brasiella dominicana* (Mandl) (Freitag 1992). The author had the opportunity during the last decade to examine *Brasiella* collected by the Carnegie Museum of Natural History staff from several widely separated populations across Hispaniola, as well as, *Brasiella dominicana* type specimens. These, and other available specimens of *Brasiella* collected from other parts of Hispaniola, exhibited many external differences from the holotype and paratypes of *Brasiella dominicana*. Adults differed superficially in body size and color, as well as, the extent and shape of elytral markings. This suggested that Hispaniola might possess more *Brasiella* species diversity than previously recognized.

Consequently, the possibility of additional *Brasiella* on Hispaniola was investigated more rigorously with a comparative morphological study of available *Brasiella* specimens from populations throughout Hispaniola. Within each population, a small sample of specimens was examined to characterize and document adult external structures for both sexes when available. Male genitalic structures, particularly those within the aedeagus inner sac, and female abdominal structures, also provided useful characters for distinguishing among *Brasiella* populations on Hispaniola. Furthermore, the characteristics of these structures provided the basis for establishing the proposed concepts of the *Brasiella* specieson Hispaniola. The results of these investigations are presented in this taxonomic revision.

This taxonomic revision of the *Brasiella* species on Hispaniola is organized by sections to provide: 1) taxonomic history; 2) sources of study specimens; 3) explanation of selective external adult structures, and internal male genitalic structures; 4) imaging methods for adults and male genitalia; 5) imaging methods for geographic distributions; 6) available ecological information and habitat imaging; 7) a dichotomous species key to identify adults; 8) detailed species accounts; 9) species relationships within the *Brasiella viridicollis* species group.

The detailed accounts for the *Brasiella* species on Hispaniola are arranged alphabetically and each includes: 1) label data from the types and other examined specimens; 2) type depositories; 3) type locality and notes on the type locality; 4) diagnostic characters for distinguishing the taxon from other taxa; 5) detailed redescription of previously known species or description of new species revealed by this revision; 6) ecological information on adult habitat and activity period, and associated tiger beetle species; 7) geographic distribution data listed and related to Hispaniola map; 8) etymology for new species; 9) remarks about comparisons with related species.

A phylogenetic analysis of *Brasiella* species on Hispaniola is beyond the scope of the present revision. However, remarks about species relationships suggested from the author's observations and experience studying the *Brasiella* species on Hispaniola are presented for each species treated in this revision. A general discussion of species relationships, based on the latest published phylogenetic treatment of *Brasiella*, follows the species accounts. Emphasis is placed on the composition of the *Brasiella virdicollis* species group, as well as, its current and ancestral geographic distributions in the West Indies. Such a discussion is considered relevant for developing future studies to investigate the phylogenetic relationships of the Hispaniola *Brasiella* species treated in this revision relative to other Western Hemisphere *Brasiella*.

## Taxonomic history

Philip J. Darlington, Jr., collected the first *Brasiella* specimens in small series on Hispaniola during his Museum of Comparative Zoology expeditions to Haiti in 1934 and the Dominican Republic in 1938. At least one specimen from the 1934 Haitian collection was determined by Walther Horn, the world authority among Cicindelidae taxonomists of the early 20^th^ Century, to be the widespread Neotropical *Cicindela argentata* Fabricius 1801. It appears that the specimens from the 1938 Dominican Republic collection were not sent to Horn because catalogers only listed this species from Haiti when considering the distribution of *Cicindela argentata* Fabricius (Blackwelder 1944) or later as *Brasiella argentata* (Fabricius)(Erwin and Sims 1984). Indeed, even (Rivalier (1954, 1955), who established the generic concept of *Brasiella* based on *Cicindela argentata* Fabricius as its type species, did not alter this view about more than one species on Hispaniola.

The prevailing view about the Hispaniolan *Brasiella* changed, however, when Karl Mandl described *Cicindela (Brasiella) dominicana* 1983 based on specimens collected in 1971 by J. Klapperich (Mandl 1983). Comparative study of a paratypic male specimen of this new species, with those Darlington had collected earlier, led Freitag (1992) to conclude that all the Hispaniola specimens he examined represented one insular endemic species, *Cicindela (Brasiella) dominicana* Mandl.

Since that publication, the author of this revision has had the opportunity to examine large numbers of *Brasiella* specimens collected at several localities on Hispaniola from 1988 to 2008. Specimens were collected as follows: large series at sites in Pedernales Province, Dominican Republic by Michael A. Ivie, WIBP; several large series at sites in Pedernales and La Vega Provinces in the Dominican Republic and Département du l'Sud, Haiti, by John E. Rawlins and Robert L. Davidson, CMNH; a small series from Santiago Province, Dominican Republic, by Brian Farrell, MCZH; small series from Peravia and La Vega Provinces, Dominican Republic, by David Brzoska. All of Darlington's *Brasiella* specimens deposited at MCZH and at NMNH were also examined. Paratypes of *Brasiella dominicana* (Mandl) at CMNH were examined, and the holotype and other paratypes of *Brasiella dominicana* were borrowed from NHMW.

## Methods

### Museum and Collection Study Specimens

This taxonomic revision was fostered by the availability of *Brasiella* specimens, including the holotype and paratypes of *Brasiella dominicana* (Mandl), from the various museums and private collections listed below under Specimen Depositories. All specimens examined are referenced with an exclamation (!). Label information is quoted in the exact format of spelling, punctuation and spacing as it appears on the labels; each label line separated by a slash between spaces ( / ); each label separated by a semicolon. The format and color used for printing each label and the label paper color are indicated in brackets. A Carnegie Museum Specimen Number label [CMNH-xxx,xxx], created by the Carnegie Museum of Natural History staff, has been affixed to all types deposited at CMNH and this unique number resides in a database at this museum. Likewise, unique numbers and/or bar codes, if previously assigned, are indicated for those type specimens deposited in other museums and collections.

### Specimen Depositories

The *Brasiella* specimens available for study were borrowed from, or deposited with, the museums and individuals listed below, and elsewhere in the revision, using accepted abbreviations (Arnett et al. 1993) or those created here for reference.

CMNH Carnegie Museum of Natural History, Pittsburgh, Pennsylvania, USA


DWBC David W. Brzoska Collection, Naples, Florida, USA


MCZH Museum of Comparative Zoology, Harvard University, Cambridge, Massachusetts, USA


MNHN Museo Nacional de Historia Natural, Santiago, Dominican Republic


NHMW Naturhistorische Museum, Wien, Austria


NMNH National Museum of Natural History, Washington, D.C., USA


WIBP West Indian Beetle Fauna Project Collection, Montana State University, Bozeman, Montana, USA


WKSU Western Kentucky State University, Bowling Green, Kentucky, USA


### Comparative Morphology

Obvious external morphological differences exhibited by specimens from different populations were utilized as the initial basis for species recognition. However, the decision about the distinctiveness of each species ultimately involved internal characters of the male genitalia, particularly the aedeagus inner sac or intromittent organ. Female external abdominal characters were utilized secondarily to support the decisions provided by the male genitalia.

### External Structures

The external structures presented below under Systematics for the key couplets to identify the species, and in their individual Species Accounts, are based on recognition of selected sclerites and appendages common to all Coleoptera. Most statements that characterize these external structures are obvious and apparent to those familiar with the study of tiger beetles; however, certain external structures typical of tiger beetles in general, and of *Brasiella* in particular, require clarification and explanation as applied specifically to this revision.

*Body Size*. In the descriptions that accompany each species, a range in body size within that species, from the largest to the smallest specimens available for study, is shown for comparison among species. Body size is presented to the nearest 0.1 mm for body length, measured dorsally from the frons of the head to the posterior tip of the elytra, and elytra width, measured dorsally across the anterior humeral angles of the elytra through the middle of the scutellum. Body length measurements reflect the position of the body when the hypognathus head is in the normal feeding position and the labrum is nearly vertical. Elytra width measurements were obtained at the most stable position on the elytra although the body is widest at the outer apical angle of the elytra. However, because the elytral suture often becomes widely separated at the apex in dried specimens, width measurements across that part of the elytra would be erroneously larger. Body size measurements were obtained with an ocular micrometer calibrated at 10 X magnification.

*Labrum Dimensions*. The labrum is a conspicuous structure in tiger beetles and has been characterized in the descriptions for most species published in the taxonomic literature for this group. For comparison of this structure among the *Brasiella* described in this revision, the dimensions of the labrum are presented as a width to length ratio for the holotype male, and a similar ratio for the known allotype female, of each species. Labral width is the transverse dimension, whereas, labral length is the longitudinal one along the body axis. The measurements of width and length were taken on the longest dimensions of the labrum.

*Eye Size*. The eye size of *Brasiella* species on Hispaniola is presented in this revision as a means for not only distinguishing between the sexes of certain species, but also between some of the species themselves. Undoubtedly, eye size provides a species specific means to acquire visual cues for mating and prey selection, as well as predator avoidance, under the prevailing light conditions within the habitat used by each particular species. Most diurnal tiger beetle species have relatively large eyes to acquire these visual cues. However, their eyes may be “prominent” in the dorsal dimension or “bulging” in the lateral dimension or have characteristics of both conditions.

The first of the two conditions of eye size used in this revision is based on the proportion of the eye surfaces dorsally above the vertex of the head. View a specimen anteriorly such that the top of the vertex between the eyes is nearly parallel to the line of sight of the viewer and the point of insertion of the posterior supraorbital setae is just visible at the eye top. Eyes are considered “prominent” if a majority of the eye surface extends above a line drawn across the inner anterior angle of each eye just below the point of insertion of the anterior supraorbital setae. The second condition of eye size is based on the proportion of the eye surfaces laterally beyond the vertex of the head. View a specimen dorsally and assess the degree of curvature of the outline of the eyes. Eyes are considered “bulging” if the eye surface occupies a larger arc area than that occupied by the vertex as defined by arc length of the curved eye boundary. For the *Brasiella* species on Hispaniola, prominent eyes are the normal condition exhibited by most species, whereas bulging eyes exist only for a few species.

*Coupling Sulcus*. The coupling sulcus is a depression, groove, pit or cavity, or a combination of these, on the female mesepisterna that receives the male mandibles during copulation. This structure was described by Freitag (1974) as an important secondary sexual mechanism for mate selection within a species. Because the characteristics of the coupling sulcus are distinctive and fairly consistent on females within a species, it can be used to differentiate closely related species and provides an additional aid to species identification. The coupling sulcus (CS) has been indicated on the anterior lateral images of adult females presented in the figures accompanying this revision for all species except one whose female is unknown.

*Abdominal Structures*. Some females of the genus *Brasiella* possess abdominal structures visible on the exposed ventral surfaces of the terminal sterna that are unique within Western Hemisphere Cicindelinae. Freitag and Barnes (1989) first noted one of these unusual abdominal characters, which they termed an “unpigmented, bell-shaped spot”, on the female 5^th^ sternum medially along its posterior margin. The color of the integument in Cicindelinae, however, is not derived from pigmentation, but rather, interference reflection of light from different epicuticular layers formed during sclerotization of the integument (Schultz and Rankin 1985a, 1985b). So rather than being unpigmented, this structure is actually membranous, unsclerotized cuticle. This transverse band on the female 5^th^ sternum in this revision is referred to as a membranous wedge (MW). Anterior to this membranous wedge on the same sternum lies a second structure, a membranous longitudinal median band (MB). Both membranous structures are developed to various degrees in most *Brasiella* species on Hispaniola for which the female is known. Transverse wrinkles in the integument also are present medially on the female 6^th^ sternum of certain *Brasiella* species on Hispaniola. In addition, a small mediolateral gibbosity (LG) situated on each side of the female 6^th^ sternum is present in all *Brasiella* species on Hispaniola for which the female is known. These structures on the female 5^th^ and 6^th^ sternum are labelled on the images presented in the figures accompanying this revision for all species except one whose female is unknown.

### Internal Structures

Male genitalia are the only internal structures discussed in this revision. Within the key couplets to species identification, and in the individual species accounts, selected sclerites very specific to *Brasiella* are referenced. Knowledge of these sclerites requires an explanation of each to understand their characteristics and help ensure their recognition. In addition to the shape and size of each sclerite, its arrangement relative to other sclerites within the aedeagus inner sac is important for comparison among species. Application of this knowledge to species identification requires careful dissection of male specimens and preparation of their genitalia to maintain the arrangement of the aedeagus inner sac sclerites.

The orientation of the male genitalia and aedeagus inner sac, as presented in this revision, is referenced based on the genitalia positioning within the abdomen. Male genitalia are normally found in this location when specimens are killed during collecting and later pinned or point-mounted for study. For an interpretation of these structures during mating, refer to Freitag et al. (1985) and the detailed explanation in Acciavatti and Pearson (1989).

*Male Genitalia*. (Rivalier (1954, 1955) characterized the genus *Brasiella* and distinguished species he studied based on distinctive differences exhibited by structures of the male genitalia. Ultimate decisions regarding distinctiveness of species in the present revision are based on the form of the male genitalia, especially the hooked tip at the aedeagus apex, and the degree of differentiation of the four internal sclerites and associated spine fields within the male aedeagus inner sac. These sclerites and spine fields are defined below, and have been indicated by their codes on images of the male aedeagus inner sac presented for all species in the figures accompanying this revision.

The four distinctive sclerites within the inner sac of the aedeagus that characterize *Brasiella* were named and defined by (Rivalier (1954, 1955). Rivalier's name for each of these structures has been translated here into English with his original French terminology shown in brackets. Each sclerite within the aedeagus inner sac is defined along with information about the range of its variation evident in Hispaniola *Brasiella* species, and its location relative to other inner sac sclerites and the genitalia itself. The code assigned here to each sclerite, and other associated structures, are shown in the images for the holotype male of each species as presented in the figures:

Arched piece [*la* *pièce arciforme*] (AP): narrow, obliquely oriented and long to broad, obliquely oriented and short; thinnest sclerite; densely sclerotized along entire length; lies dorsally across other sclerites; not visible externally.

Shield [*un bouclier*] (SH): distinct, single apex with rounded tip to distinct, single apex with tapering tip; smallest sclerite; not densely sclerotized; occupies lower end of genital opening; entire distal end distinctly visible externally.

Large tooth [*la grande dent*] (LT): elongate with a pointed apex to broad with a rounded apex; most bulky sclerite; densely sclerotized except at distal end; extended basally into a densely sclerotized root; not visible externally.

Stylet [*le stylet*] (ST): long, broad and straight with pointed tip to long, broad and bent with recurved or hooked tip; longest sclerite; not densely sclerotized; occupies upper end of genital opening; tip visible externally.

Additional structures within the inner sac were characterized as follows:

Spine Fields (SF): several patches of sclerotized setae of various sizes and shapes, the one within the aedeagus neck being the one most obvious and diagnostic; others are positioned inside the genital opening on either side of it.

Dark Fields (DF): darkened, membranous areas near Root of Large Tooth.

Root (RT): densely sclerotized, curved and forked, basal extension of Large Tooth.

*Male Genitalia Preparation*. A few adult males, representative of the different populations of *Brasiella* available from Hispaniola, were each prepared for removal of the genitalia by soaking the entire specimen in a weak solution of ammonia for a day or two. The aedeagus could then be easily pulled out or removed from each relaxed abdomen, and through gentle squeezing with fine forceps, the four sclerites and associated setal brushes within its inner sac could be extruded for examination and imaging. For future reference as vouchers, the aedeagus of each sampled specimen has been stored in glycerin in a microvial and this pinned underneath the specimen. These specimens have been deposited with the museum or in the collections shown above under the Specimen Depositories utilized for this revision.

### Imaging External and Internal Structures

Adult habitus images of the holotypes, allotypes, and certain paratypes of the *Brasiella* species described and presented in this revision are presented as Figs 1–17, as well as, close-ups of selected body regions, and male genitalic structures. These images not only help to illustrate the structures, but also provide a means for comparing characters among the species to facilitate their recognition.

Images of adults of each sex from various aspects were obtained with a JVC 3CCD Digital Camera, Model KY-F74U, through a Leica M420 APO, Zoom 1:6, stereomicroscope equipped with Syncroscopy Auto-Montage Pro Version 5.01.005 2004 software. Standard fiber optics lights were used to illuminate all specimens imaged with the light diffused by white translucent surfaces.

Male genitalic and aedeagal images were obtained using a Nikon CoolPix L5, 7.2 megapixel, 5 X zoom, digital camera through a Bausch and Lomb zoom stereomicroscope. To facilitate imaging of the male genitalia and the aedeagus with its extruded inner sac sclerites, these structures were placed in glycerin within the well of a glass slide. The glass slide was suspended above neutral gray photographic paper. Thus suspended, the aedeagus was illuminated by two fiber optics lights adjusted from various angles to provide both back lighting and minimize glare.

All images were cropped and photo enhanced with standard photo editor software on a personal computer. The images illustrate the general body form, shape and color of structures, elytral markings, and certain specific morphological characters discussed in the key and descriptions. A 1 mm scale line was added to each enhanced image to indicate the relative size of comparable morphological structures among species.

### Geographic Distributions

For each *Brasiella* species on Hispaniola, its type locality and other localities are presented on the 30 m resolution, digital, topographic map shown as Fig. 22. This map was generated from radar-imaged elevation data on a near-global scale obtained through the NASA Shuttle Radar Topography Mission (SRTM) on board the Space Shuttle Endeavor in February 2000. The map image is in the public domain and available through the Internet. No political boundaries are shown on the map, as these were deemed unnecessary for the purposes of depicting the known distribution of each species in relation to the various mountainous areas on Hispaniola. The legend accompanying Fig. 22 provides a descriptive name for the color-coded circle(s) for each *Brasiella* species to indicate the type locality and/or other localities within either the Dominican Republic Province or Haiti Département, along with the geographic feature associated with the type locality.

### Ecology

The known ecological information concerning the adult habitat and activity period for the species treated in this revision come mostly from label data. This has been augmented by field collection notes provided by Robert L. Davidson, who collected several of the new *Brasiella* species, and from John E. Rawlins, who led the CMNH Expeditions to the Dominican Republic from 1987 through 2003, and to Haiti in 1995. David W. Brzoska provided information on the habitat and associated tiger beetle species for the *Brasiella* specimens he collected in the Dominican Republic in 2005.

Woodruff (2004) presented detailed information about the habitats in the Sierra de Baoruco, Dominican Republic, thereby providing a valuable insight into the ecological settings for several sympatric *Brasiella* in this mountain range. He considered the Sierra de Baoruco to be an area of high endemism for Coleoptera because it lies on what has been referred to as the South Paleoisland of Hispaniola. Woodruff (2004) provided a general account of the geological evidence about Paleoisland concepts for Hispaniola to explain the zoogeography of *Phyllophaga* species (Coleoptera: Scarabaeidae: Melolonthinae) on this island. For further reading on the geologic history of Hispaniola, refer to his publication (Woodruff 2004) as a discussion of these concepts is beyond the scope of this revision.

An indication of the habitat for each *Brasiella* species on Hispaniola is presented as oblique aerial images obtained from Google Earth©. Ground level habitat images also are presented for certain of these species obtained as geotagged images from either websites, http://acme.mapper.com, and http://creativecommons.org, using http://flickr.com by Yahoo©, or from scanned slides and images taken by John E. Rawlins during the CMNH expeditions to the Dominican Republic. Figs 18 to 21 depict these habitats.

## Systematics

Identification Key to Adult *Brasiella* Species on Hispaniola

**Table:** 

1	Elytral markings faint or indistinct, pale tan or translucent when evident, or completely absent; faint markings only slightly contrasting with a darker background surface scattered with metallic blue or green flecks of various sizes that may obscure markings, apical lunule may be present	2
1'	Elytral markings in all specimens clearly visible, bold and distinct, colored white or tawny, distinctly contrasting with a darker background surface scattered with metallic blue or green flecks of various sizes that remain separated from markings; most specimens marked as noted, other specimens may have reduced markings, but apical lunule always present	4
2	Head small, eyes proportionally large and distinctly bulging laterally; pronotum shape dorsally subglobose to subarcuate; suture between clypeus and labrum abruptly arcuate; appendages primarily testaceous, translucent, in female, appendages mainly dark metallic in male; female 8th sternum median notch shallowly incised (Dominican Republic: Pedernales Province, Sierra de Baoruco, southern slope, lower elevations)	*Brasiella youngi*, new species
2'	Head large, eyes proportionally small and only slightly or not bulging laterally; pronotum shape dorsally square to slightly arcuate; suture between clypeus and labrum broadly arcuate; appendages in both sexes dark, opaque, dominated by metallic reflections, only trochanters testaceous; female 8th sternum median notch deeply incised (Dominican Republic: Pedernales Province, Sierra de Baoruco, southern slope, higher elevations)	3
3	Larger species, body size of males > 6.5 mm, of females > 7.0 mm; head longer than pronotum in female, head and pronotum of similar length in male; eyes larger, slightly bulging laterally; elytral markings obvious in most specimens, especially females; male genitalia apical neck short and wide, tip acutely bent and tapering evenly to an elongated point; aedeagus inner sac stylet broad and recurved distad (Dominican Republic: Pedernales Province, Sierra de Baoruco, Las Abejas)	*Brasiella iviei*, new species
3'	Smaller species, body size of males ≤ 6.5 mm, of females ≤ 7.0 mm; head and pronotum of similar length in both sexes; eyes smaller not bulging laterally; elytral markings obscure in most specimens; male genitalia apical neck long and narrow, tip acutely bent and tapering abruptly to a short point; aedeagus inner sac stylet thin and straight to slightly bent distad (Dominican Republic: Pedernales Province, Sierra de Baoruco, Aceitillar)	*Brasiella rawlinsi*, new species
4	Elytra marked with a complete pattern consisting of three isolated markings (apical lunule entire, middle band sinuate usually slightly expanded at lateral margin and enlarged or recurved anteriorly near suture, humeral lunule complete or only slightly broken at posterior discal end) (Dominican Republic)	5
4'	Elytra marked with reduced pattern consisting of four or five isolated markings (apical lunule entire or broken into marginal band and subapical dot, middle band sinuate never expanded laterally nor recurved anteriorly near suture, humeral lunule broken into two widely separated terminal dots, one at humeral angle and one on disc) (Haiti)	8
5	Body size smaller, < 6.0 mm; head and pronotum shiny copper red, markedly contrasting with darker elytra; head wide, eyes slightly bulging laterally; pronotum square; male aedeagus inner sac stylet tip recurved, shield angled distad, large tooth short, narrow and pointed at tip, arched piece short and thick; female unknown (Dominican Republic: Peravia Province, Rio Ocoa flood plain near south coast)	*Brasiella ocoa*, new species
5'	Body size larger, ≥ 6.0 mm; head and pronotum dull black brown to copper, not contrasting with elytra; head width, eyes bulging or not; pronotal width varied; male genitalia inner sac stylet tip bent, shield rounded distad, large tooth long and pointed at tip, arched piece long and thin; females known for species compared in next couplets	6
6	Elytral middle band distinctly recurved anteriorly near suture; male genitalia bulky, aedeagus distal neck short and broad, apical spine field forming a short and wide pad; aedeagus inner sac apical hook evenly rounded, tip elongated and nearly at right angle to aedeagus, inner sac stylet tip recurved, shield tapered distad; female 5th abdominal sternum with narrow membranous band along midline, and a small membranous wedge along posterior margin (Dominican Republic: Santiago Province, Cordillera Central)	*Brasiella philipi*, new species
6'	Elytral middle band not distinctly recurved near suture; male genitalia slim, aedeagus distal neck long and narrow, apical spine field forming a long and narrow pad; apical hook abruptly rounded, tip shortened and at acute angle to aedeagus, inner sac stylet tip bent, shield rounded distad; female 5th abdominal sternum with wide membranous band along midline, and a large membranous wedge along posterior margin	7
7	Elytral apices obliquely curved, sutural spine strongly withdrawn from apex; elytral markings cream to pale white, broad, humeral lunule and middle band of constant width medially on disc in most specimens, only humeral lunule slightly broken at posterior discal end in others; eyes slightly bulging laterally; head, pronotum and proepisterna shiny dark copper brown; aedeagus inner sac stylet long, stylet tip slightly bent and evenly tapered to a narrow point, large tooth long, broad and pointed at tip with large dark fields near roots; small lateral gibbosities on 6th female sternum (Dominican Republic: Peravia Province, Rio Bani south of Cordillera Central)	*Brasiella dominicana* (Mandl)
7'	Elytral apices evenly rounded, sutural spine feebly withdrawn from apex; elytral markings tawny, narrow, humeral lunule and middle band thinned medially on disc in most specimens, both markings slightly broken at posterior discal end in others; eyes not bulging laterally; head shiny black brown to blue brown; pronotum black brown to copper; proepisterna shiny black green; aedeagus inner sac stylet short, stylet tip bent and unevenly tapered to a broad point rounded in some specimens or sharply pointed in others, large tooth short, broad and rounded at tip with small dark fields near root; large lateral gibbosities on 6th female sternum (Dominican Republic: La Vega Province, Cordillera Central)	*Brasiella bellorum*, new species
8	Labrum anterior margin protruding broadly mesad with a small medial tooth; eyes smaller, neither prominent nor bulging laterally; elytra marked anteriorly with a small, oval discal spot, middle band and apical lunule wide; male genitalia broadly and evenly curved along aedeagus neck, apex terminating in an extremely short, blunt hook; aedeagus inner sac stylet hooked at terminal tip, shield tapered distad, short and narrow apical spine field in aedeagus neck; female 5th abdominal sternum without a membranous band or wedge along posterior margin (Haiti: Département du Sud, Département du Grand' Anse, Massif de la Hotte)	*Brasiella davidsoni*, new species
8'	Labrum anterior margin nearly straight to feebly protruding mesad with a tiny medial tooth; eyes larger, prominent, but not bulging laterally; elytra marked anteriorly with a small, circular discal dot, middle band and apical lunule narrow; male genitalia straight along aedeagus neck, apex terminating in a long acute hook; aedeagus inner sac stylet tip recurved at terminal tip, shield rounded distad, short and wide apical spine field in aedeagus neck; female 5th abdominal sternum with a short, wide membranous medial band anterior to a wide membranous wedge along posterior margin (Haiti: Départment du l'Ouest, Massif de la Selle)	*Brasiella darlingtoniana*, new species

## Species Accounts

### Brasiella bellorum sp. n.

urn:lsid:zoobank.org:act:04AF5BBF-F5E7-4A01-9737-B4EB84C9D8C9

http://species-id.net/wiki/Brasiella_bellorum

Figs 1 2

#### Holotype.

Male! labeled “DOMINICAN REPUBLIC: La / Vega. Cordillera Central / 4.1 km SW El Convento. /18-50-37N, 70-42-48W, / 1730 m, 31 May 2003” [typeset black on white label]; “J. Rawlins, R.Davidson, / C. Young, C. Nunez, P. / Acevedo,dense secondary / evergreen forest with / pine, hand collected / Sample 22242” [typeset black on white label]; “Carnegie Museum / Specimen Number / CMNH-310,643” [typeset black on white label]; “HOLOTYPE / Brasiella / bellorum / Acciavatti” [typeset black on red label]. [Genitalia in glycerin in a microvial pinned beneath specimen.]

#### Allotype.

Female! labeled with same locality data as the holotype; “Carnegie Museum / Specimen Number / CMNH-307,997” [typeset black on white label] “ALLOTYPE / Brasiella / bellorum / Acciavatti” [typeset black on red label].

#### Paratypes.

Specimens! as follows: 1) 17 males and 38 females labeled with the same locality data as the holotype; “PARATYPE / Brasiella / bellorum / Acciavatti” [typeset black on blue label]; [these paratypes each labeled with a CMNH Unique Number on file]; 2) 1 male and 1 female labeled “DOMINICAN REPUBLIC: La / Vega. Cordillera Central / 4.1 km SW El Convento. /18-50-37N, 70-42-48W, / 1710 m, 14 November 2002” [typeset black on white label]; “W.A. Zanol, C. Young, / C. Staresinic, J. Rawlins, / secondary broadleaf / forest, hand collected / Sample 20249” [typeset black on white label]; “PARATYPE / Brasiella / bellorum / Acciavatti” [typeset black on blue label]; these paratypes each labeled with a CMNH Unique Number; 3) 6 males and 4 females labeled “DOM.REP.-LA VEGA / PROV.-Constanza-Rancho / Guaraguao, 1538m / 18°53.1'N, 70°41.2'W / D. Br / zoska 19-X-2005” [typeset black on white label]; “PARATYPE / Brasiella / bellorum / Acciavatti” [typeset black on blue label].

#### Additional Specimens.

Two specimens! (one each sex), not paratypes, labeled “Constanza / Aug. '38, Dom. Rep. / 3-4000 ft. / Darlington” [typeset black on white label]; “C. (Brasiella) / dominicana Mandl / det. R. Freitag / April 1988” [typeset black on white label]; “Brasiella / bellorum / Acciavatti” [typeset black on white label]. These specimens appear to be conspecific with the holotype based on the male genitalia, but the female specimen has most of the middle band missing, although the humeral and apical lunules match specimens from the type series. Because of these slightly different elytral markings, and their lack of a specific collection site, these specimens were not designated paratypes. Both of these specimens are at MCZH.

#### Type Depositories.

Holotype, allotype, 55 paratypes at CMNH, each with CMNH Unique Number stored in data files at CMNH. Ten paratypes at DWBC.

#### Type Locality.

DOMINICAN REPUBLIC: La Vega Province, Cordillera Central, 4.1 km SW El Convento, 18°50'37"N, 70°42'48"W, 1730 m. Aerial view in Fig. 18A.

#### Diagnosis.

Distinguished from other *Brasiella* species on Hispaniola by the following combination of characters: 1) head shiny black brown, blue brown, or copper; 2) pronotum shiny black brown to copper, color not contrasting with duller elytral color; 3) proepisterna shiny black green to copper; 4) eyes prominent, not bulging laterally; 5) elytral apices evenly rounded with sutural spine feebly withdrawn from apex; 6) elytral markings tawny, narrow, slightly contrasting with the darker elytral ground color; humeral lunule and middle band narrow, thinned or broken medially ending in an enlarged spot on disc; apical lunule narrow; 7) male genitalia with long aedeagus neck and a short, recurved apex; 8) aedeagus apical spine field forming a long and narrow pad; 9) aedeagus inner sac stylet short, tip bent and unevenly tapered to a broad point rounded in some specimens or sharply pointed in others; 10) large tooth short, broad and rounded at tip; shield rounded distad; arched piece long and thin; 11) lateral gibbosities on 6th female sternum large.

#### Description.

**Figure 1.**
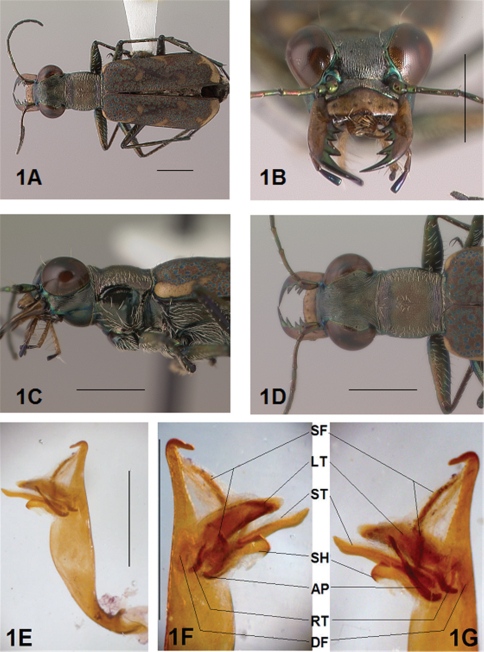
*Brasiella bellorum,* sp. n., male. Holotype **1A** Body, dorsal **1B** Head, anterior **1C** Body, anterior, left lateral **1D** Body, anterior, dorsal **1E** Aedeagus, dorsal **1F** Aedeagus inner sac, ventral aspect, and **1G** Aedeagus inner sac, dorsal aspect–AP, arched piece; LT, large tooth; SH, shield; ST, stylet; SF, spine fields (one displaced from within aedeagus neck and two isolated); RT, root of LT; DF, dark fields. [Scale lines = 1 mm].

**Figure 2.**
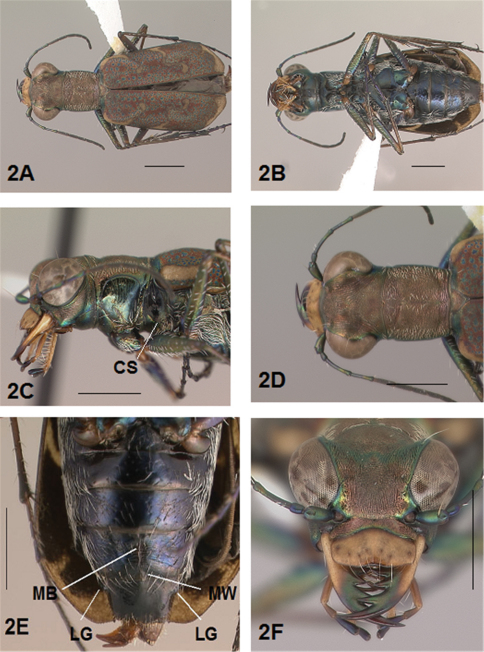
*Brasiella bellorum,* sp. n., female. Allotype **2A** Body, dorsal **2B** Body, ventral **2C** Body, anterior, left lateral–CS, coupling sulcus **2D** Body, anterior, dorsal **2E** Abdomen, sterna, ventral–5^th^ sternum, MB, membranous band; MW, membranous wedge; 6^th^ sternum, LG, lateral gibbosity **2F** Head, anterior. [Scale lines = 1 mm].

*General.* Figs 1A, 2A. *Body.* Formelongate; head broad, eyes prominent, not bulging laterally; pronotum square; elytra broadened distad, apices evenly and separately rounded. *Size*.Males, length 6.3-6.8 mm, width 2.1-2.2 mm; females, length 6.7-7.2 mm, width 2.2-2.4 mm.

*Head*. Figs 1B, 1D, 2D, 2F. Shiny dark black brown, blue brown or copper dorsally, shiny black green ventrally; entire surface glabrous except for two pairs of supraorbital sensory setae. Frons finely and longitudinally rugose. Vertex more coarsely rugose, transverse rugae along anterior margin narrow and irregularly arranged, 13-16 more or less complete longitudinal rugae between eyes and middle where rugae converge into an arcuate pattern; rugae transition abruptly into a posterior area with a finely and irregularly granulate surface. Eyes prominent, not bulging laterally, less prominent in female than male. Genae longitudinally rugose. Clypeus finely and irregularly granulate, narrowed mesad. Labrum testaceous with a dark brown margin, subrectangular, width to length ratio 3 in holotype male, ratio 2.5 in allotype female; anterior margin slightly sinuate, medial tooth minute or absent, sinuation larger, more prominent at middle in female than in male; posterior margin distinctly arcuate mesad; medial carina broad and distinctly raised; 6-9 setae in an irregular row near middle arranged on either side of medial carina. Maxillae and labium mainly testaceous, only distal palpal segments dark brown with metallic blue green reflections. Mandibles sexually dimorphic; in male, surface mainly testaceous, only teeth metallic green; in female, surface only testaceous in basal half, apical half and teeth shiny brown; mandibles symmetrical, four teeth distad of molar, apical tooth longest, first and third tooth coequal in length, second tooth shortest; gaps between teeth wider in female than in male; first and second teeth without a gap between them in male. Antennae 11 segmented; scape dorsally shiny green and ventrally pale yellow, occasionally pale yellow with a single subapical sensory seta; antennomeres 2-4 shiny green, glabrous except for a few, short erect setae along their length and distally; antennomeres 5-11 dull brown, sheathed with dense short sensory setae.

*Prothorax.* Figs 1C, 1D, 2C, 2D. Pronotum shiny black brown to copper. Proepisterna shiny black green to copper, surface wrinkled dorsad. Prosternum shiny green. Pronotum glabrous except for short, decumbent, white setae distributed in several, irregular rows medially directed, originating close to and lying in a narrow band distinctly removed from lateral suture, in a sparse narrow band transversely and anteriorly oriented within broad anterior margin, and in a sparse narrow band laterally oriented on each side of midline extending nearly to the narrow posterior margin; transverse submarginal sulci distinct, anterior sulcus shallow, posterior sulcus deeper and deepest at posterior angles; transverse rugae within broad anterior margin irregular and shallow, interrupted at middle by an irregularly arranged pattern, within posterior margin more distinctly and deeply engraved especially medially and extending onto midline; surface sculptured by fine, transverse rugae angled on disc and interrupted by a finely engraved longitudinal midline, and more finely and irregularly sculptured elsewhere. Proepisterna glabrous except for white, erect and appressed setae arising from small setigerous punctures scattered over most of the surface in males, only in ventral half in females. Prosternum glabrous, surface smooth.

*Pterothorax.* Figs 1C, 2C. Mesepisterna glabrous except for appressed setae near ventral margin; female coupling sulcus represented by a shallow, circular depression medially situated with a slightly deeper center, a distinct groove extends only dorsally from center, surface smooth below center. Mesepimeron with sparse appressed setae. Metepisterna with scattered appressed setae, more abundant in male than female. Prosternum and mesosternum glabrous, smooth to slightly wrinkled; metasternum glabrous except for long, dense white appressed setae laterad, surface smooth mesad and coarsely sculpted laterad where setae originate. Scutellum triangular, cupreous.

*Legs.* Figs 1A, 2B. Segmentstestaceous brown with metallic brown green reflections. Coxae shiny metallic brown green; trochanters testaceous or dark brown; femora and tibiae testaceous with metallic green reflections anteriorly in most specimens, metallic green except for testaceous distal end of each segment in other specimens; tarsomeres dark metallic violet black or brown; white, appressed setae on front and middle coxae, and laterally on hind coxae; erect setae and suberect closely spaced forming several regular and irregular rows on all femora; setae widely spaced in a few rows on all tibiae; middle tibiae with patch of appressed setae dorsally along distal half; tarsomeres with short scattered setae on ventral surface; distal tarsomeres with two asymmetrical rows each with a few to several small, erect setae; an erect subapical seta present only on front trochanter, absent on middle and hind trochanters; males with dense pad of erect setae ventrally on proximal three tarsal segments; tarsal claws small.

*Elytra.* Figs 1A, 2A. Form narrow in male, broadened distad and broadest at outer apical angle in female; evenly curved along posterior margins with apices separately rounded; sutural spine small and inconspicuous, feebly withdrawn from apex; posterior margins finely microserrulate. Surface finely granulate, impunctate, numerous small, irregular, metallic green or blue green flecks of various sizes scattered over a dull, dark copper brown background; elytral pattern thin, narrow slightly contrasting with the darker elytral ground color; setigerous punctures with short, erect, transparent setae indistinct in subsutural rows on disc, but distinct at elytral base, and at inner humeral angles, each surrounded by a metallic fleck slightly larger than flecks elsewhere on elytra; surface slightly depressed in humeral area and on disc creating a slight but distinct raised area basally. Elytral markings tawny, forming pattern of narrow markings reduced in width in most specimens, or partially missing in other specimens; pattern consisting of a narrow humeral lunule thinned medially and broken before terminating as a spot on disc in most specimens, or reduced to a small dot in other specimens; middle band sinuate with irregular margins, thinned medially or broken, slightly enlarged near suture, slightly expanded along lateral margin in most specimens, or reduced to small terminal spots in a few specimens; apical lunule narrow and broadened along suture in all specimens examined. Elytral epipleura testaceous except for a narrow, metallic green to copper green band along dorsal margin.

*Abdomen.* Figs 2B, 2E. Surface of 1st-5th sterna shiny black with green reflections, 6th sternum entirely shiny black to black brown; posterior margins of male 3rd-5th sterna and female 3rd-4th sterna narrowly black; posterior margin female 5th sternum broadly black; 3rd-5th sterna medially smooth with scattered, fine, erect setae in both sexes; male 1st-6th sterna and female 1st-5th sterna laterally covered with dense, scattered, appressed white setae and roughened from setal punctures; male 6th sternum glabrous medially with a broad, deep concave notch; female 5th abdominal sternum with moderately raised transverse wrinkles and a wide membranous band at midline extending anteriorly along most of the sternum from a large membranous wedge along posterior margin; female 6th sternum entirely glabrous, posterior margin with a row of 6-10 erect spines and with a large lateral gibbosity on each side.

*Male Genitalia*.Figs 1E, 1F, 1G. Shape narrow near base, broad in middle half, slim distally with neck short and narrow, apical hook evenly rounded, tip shortened and at acute angle to aedeagus, aedeagus apical spine field forming a long and narrow pad. Aedeagus inner sac sclerites: stylet tip short and bent; shield rounded distad; large tooth short, broad and rounded at tip with large root and small dark fields; arched piece long and thin.

#### Ecology.

This species occurs on the clay slopes of road cuts and adjacent dirt roads within dense secondary evergreen and broadleaf forests with pines on slopes in the central parts of the Cordillera Central between 915 to 1730 m elevations. At the type locality, adults and larvae of this species were present and abundant in May when Robert L. Davidson collected the large type series (Figs 18B-18D). The two adults taken the previous November at the type locality may not truly represent how abundant this species can be at that time year when less avid collectors visited the type locality. However, this species may actually be less abundant in November than earlier in May. Indeed, David W. Brzoska, who is an accomplished tiger beetle collector, was only able to collect 11 specimens of *Brasiella bellorum* in November at a locality at 1538 m only about 5 km away from the type locality. The specimens he collected were found near wet areas around puddles on exposed red clay along a grassy dirt road. Thus, adult activity for *Brasiella bellorum* spans seven months with adults more abundant in May than later in August and November.

#### Distribution.

Fig. 22. DOMINICAN REPUBLIC: La Vega Province, El Convento, Rancho Guaraguao, and Constanza. This species likely occurs in suitable habitats throughout the Constanza Valley and surrounding mountainous areas of the central portions of the Cordillera Central.

#### Etymology.

This Latinized plural eponym, genitive case, based on the family name of Ross T. Bell honors both Ross and his wife, Joyce, for their entomological careers and contributions as world authorities on the Rhysodidae. Ross and Joyce have been friends, colleagues, and mentors to a multitude of entomologists and taxonomists specializing in Carabidae. Both Ross and Joyce Bell were honorees of the Bell Fest Symposium held in Burlington, Vermont, June 2010, and this Festschrift is dedicated to them.

#### Remarks.

*Brasiella bellorum*, new species, and *Brasiella philipi*, new species, both occur in the Cordillera Central, Dominican Republic, the former species in the central areas and the latter species on the northern slopes of this mountain range. The elytral pattern of both species are very similar and only differ in the form of the elytral lunules; narrowed and broken in *Brasiella bellorum*, but wide and more developed in *Brasiella philipi*, especially in the shape of the hook at the discal end of the middle band. Despite this superficial similarity, there seems little doubt that these two species are distinctive based on differences in the form of the sclerites within the aedeagus of males for each species. A comparison of the other morphological characters presented in the key and the descriptions for each species further support their distinctiveness. The geographic range of both these species, although apparently allopatric based on the limited collection data currently available, may be found to coincide more with additional collecting in the Cordillera Central. Despite their distinctiveness as separate species and apparent allopatry, it is interesting to note that these two species seem to occupy a similar habitat type in these mountains with adults of both species active during the same summer months. Their geographic distributions in close proximity to each other in similar high elevation habitats suggest a common lineage.

### Brasiella darlingtoniana sp. n.

urn:lsid:zoobank.org:act:B07061D3-CB05-43C1-AA3E-E1B32D81D97F

http://species-id.net/wiki/Brasiella_darlingtoniana

Figs 3 4

#### Holotype.

Male! labeled “Kenskoff to / La Visite& vic / LaSelleRange” [first line handprinted, others typeset, black on white label]; “Haiti / 5000ft / 1934 IX.16 / Darlington” [first and fourth lines, and year, typeset, others handprinted, black on white label]; “C. (Brasiella) / dominicana Mandl / det. R. Freitag / April 1988” [typeset black on white label]; “HOLOTYPE / Brasiella / darlingtoniana / Acciavatti” [typeset black on red label]. [Genitalia in glycerin in a microvial pinned beneath specimen.]

#### Allotype.

Female! labeled “Kenskoff -- / La Visite& vic / LaSelleRange” [first line handprinted, others typeset, black on white label]; second label same locality data as holotype; “Dr.W.Horn det.1935 / Cic.argenta- / ta F.”; [first line typeset, ‘35' and next two lines handprinted black on white paper]; same Freitag det. label as holotype; “ALLOTYPE / Brasiella / darlingtoniana / Acciavatti” [typeset black on red label].

#### Paratypes.

Specimens! as follows: 1) 5 males and 3 females same locality data as the holotype and allotype; labeled either with same Freitag det. label or “Cicindela / argentata / Fab.” or “argentata / det. F. / Darlington ♂” or “argentata / det. F. / Darlington ♀” or “A. Nicolay / collection / 1950” and “USNM / 2054986”; “PARATYPE / Brasiella / darlingtoniana / Acciavatti” [typeset black on blue label]; [five of these paratypes each also labeled with a CMNH Unique Number (see below)]; 2) 1 female labeled “Auto Rd Furcy- / Kenskoff / Mf.LaSelle / 5000ft IX.23” [handprinted black on white label]; “Haiti / 1934 / Darlington” [typeset black on white label]; “argentata / W.Horn.det” [typeset black on white label]; same Freitag det. label, “PARATYPE / Brasiella / darlingtoniana / Acciavatti” [typeset black on blue label].

#### Type Depositories.

Holotype, allotype, 2 paratypes (one each sex) at MCZH; 2 male paratypes at NMNH; 5 paratypes (3 males, 2 females) at CMNH. [Five male paratypes at CMNH with Carnegie Museum Specimen Numbers: CMNH-122,066; 530,700; 533,849. Female paratypes at CMNH with Carnegie Museum Specimen Numbers: CMNH-488,300; 495,484. CMNH Unique Number stored in data files at CMNH.]

#### Type Locality.

HAITI: Département du l'Ouest, Massif de la Selle, Highway 101 below Parc de La Visite, 18°20'45"N, 72°17'19"W, 1755 m. Aerial view in Fig. 18E.

*Notes on Type Locality*. The type locality, herein established at the above coordinates, was implied from the type labels because the actual location where Darlington collected this new species is uncertain; his label data only generalized his collecting along the route he took during his expedition to Haiti. La Visite is a National Park, Parc de La Visite, located along Highway 101 in a relatively flat, high elevation area along this highway at 6000 feet in the Massif de la Selle south of Port-au-Prince, Haiti. However, Darlington almost certainly did not collect this new species within La Visite National Park based on the discussion presented below under Ecology. In consideration of that information, the type locality for this species is more precisely established above using Google Earth©. The type locality was chosen to be a site adjoining La Visite slightly to the north (Fig. 18F) with the coordinates shown above.

#### Diagnosis.

Distinguished from other *Brasiella* species on Hispaniola by the following combination of characters: 1) elytral markings a humeral lunule reduced to its extremities, a middle lunule terminating near the suture in a broad hook, never anteriorly recurved, a narrow apical lunule; 2) eyes prominent, not bulging laterally; 3) males with a long, acutely curved aedeagus apex with an inner sac having a recurved terminal tip of the stylet; 4) females with a short, wide membranous band anterior to a wide membranous band along posterior margin of 5th abdominal sternum.

#### Description.

**Figure 3.**
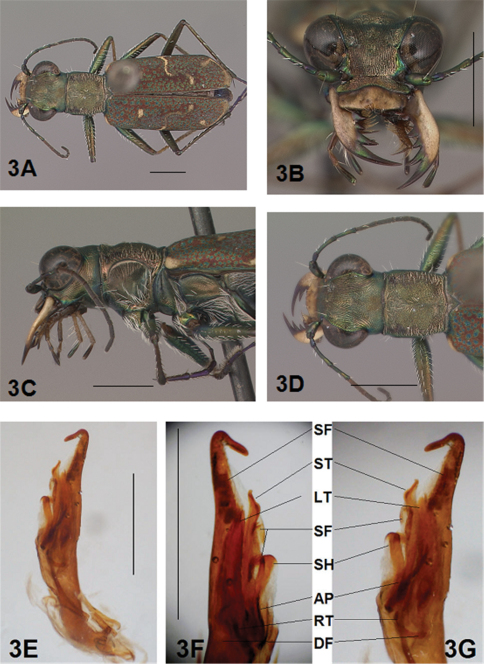
*Brasiella darlingtoniana*, sp. n., male. Holotype **3A** Body, dorsal **3B** Head, anterior **3C** Body, anterior, left lateral **3D** Body, anterior, dorsal **3E** Aedeagus, dorsal **3F** Aedeagus inner sac, ventral aspect, and **3G** Aedeagus inner sac, dorsal aspect–AP, arched piece; LT, large tooth; SH, shield; ST, stylet; SF, spine fields (one within aedeagus neck and two isolated); RT, root of LT; DF, dark fields. [Scale lines = 1 mm].

**Figure 4.**
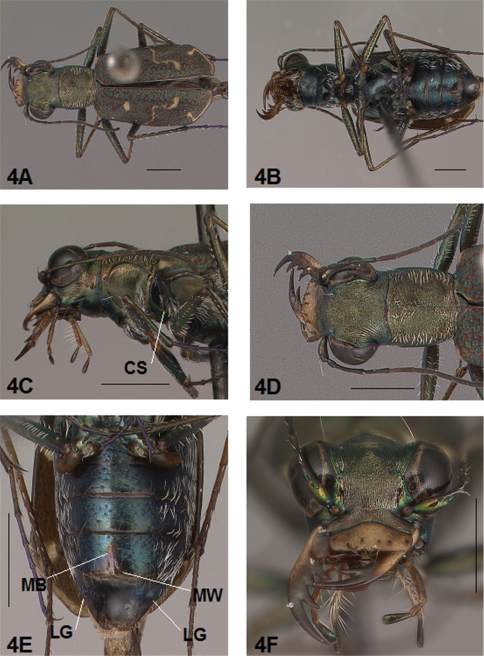
*Brasiella darlingtoniana*, sp. n., female. Allotype **4A** Body, dorsal **4B** Body, ventral **4C** Body, anterior, left lateral–CS, coupling sulcus **4D** Body, anterior, dorsal **4E** Abdomen, sterna, ventral–5^th^ sternum, MB, membranous band; 5^th^ sternum, MW, membranous wedge; 6^th^ sternum, LG, lateral gibbosity **4F** Head, anterior. [Scale lines = 1 mm].

*General.* Figs 3A, 4A. *Body.* Formelongate; head wide, eyes prominent, not bulging laterally; pronotum wide, square; elytra slender, slightly broadened distad, apices separately rounded. *Size*.Males, length 6.4-6.6 mm, width 2.0-2.1 mm; females, length 6.6-6.9 mm, width 2.1-2.2 mm.

*Head*. Figs 3B, 3D, 4D, 4F. Shiny green bronze with copper reflections dorsally and black green ventrally; entire surface glabrous except for two pairs of supraorbital sensory setae. Frons finely and longitudinally rugose. Vertex more coarsely rugose, rugae along anterior margin irregularly arranged, 15-18 more or less complete longitudinal rugae between eyes and middle where rugae merge to form a circular pattern at middle of vertex, rugae transition abruptly into a posterior area with a finely and irregularly granulate surface. Eyes prominent, not bulging laterally. Genae longitudinally rugose. Clypeus finely and irregularly granulate, narrowed mesad. Labrum testaceous with a dark brown margin, rectangular, width to length ratio 3 in holotype male, ratio 3.1 in allotype female; anterior margin nearly straight to feebly protruding mesad with a tiny medial tooth in both sexes; posterior margin broadly arcuate mesad; medial carina distinctly raised with a slight depression on each side near posterior margin; 6 setae symmetrically arranged, anterior setae in a row, lateral setae closer to clypeus. Maxillae and labium mainly testaceous, only distal palpal segments dark brown with metallic blue green reflections. Mandibles sexually dimorphic; in male, surface mainly testaceous, only teeth metallic green; in female, surface only testaceous in basal half, apical half and teeth shiny brown; mandibles symmetrical, four teeth distad of molar, apical tooth longest, first and third tooth coequal in length, second tooth shortest; gaps between three intermediate teeth equally wide in both male and female. Antennae 11 segmented; scape dorsally shiny green, ventrally testaceous with a single subapical sensory seta; antennomeres 2-4 shiny green, glabrous except for a few, short erect setae along their length and distally; antennomeres 5-11 dull brown, sheathed with dense short sensory setae.

*Prothorax.* Figs 3C, 3D, 4C, 4D. Pronotum shiny, dark copper brown. Proepisterna shiny, dark copper brown, surface wrinkled dorsad. Prosternum shiny green. Pronotum glabrous except for short, decumbent, white setae distributed in several, irregular rows medially directed, originating close to, and lying in a narrow band distinctly removed from lateral suture, in a sparse narrow band transversely and anteriorly oriented within broad anterior margin, and in a sparse narrow band laterally oriented on each side of midline extending only to the middle, surface glabrous from middle to posterior margin and along this margin; transverse submarginal sulci distinct, anterior sulcus shallow, posterior sulcus deeper and deepest at posterior angles; transverse rugae within broad anterior margin irregular and shallow, interrupted at middle by an irregularly arranged pattern, within posterior margin more distinctly and deeply engraved especially medially and extending onto midline; surface sculptured by fine, transverse rugae angled on disc and interrupted by a finely engraved longitudinal midline, and more finely and irregularly sculptured elsewhere. Proepisterna glabrous except for white, erect and appressed setae arising from small setigerous punctures scattered over most of the surface in males, only in ventral half in females. Prosternum glabrous, surface smooth.

*Pterothorax.* Figs 3C, 4C. Mesepisterna glabrous except for appressed setae near ventral margin, more abundant in male than female; female coupling sulcus represented by a shallow, circular depression medially situated with a slightly deeper center, a distinct groove extends only dorsally from center, surface smooth below center. Mesepimeron with sparse appressed setae. Metepisterna with scattered appressed setae, more abundant in male than female. Prosternum and mesosternum glabrous, smooth to slightly wrinkled; metasternum glabrous except for long, dense white appressed setae laterad, surface smooth mesad and coarsely sculpted laterad where setae originate. Scutellum triangular, cupreous green.

*Legs.* Figs 3A, 4B. Segmentsmetallic green with copper reflections and testaceous areas. Coxae shiny metallic brown green; trochanters shiny testaceous; femora metallic green with copper reflections except for testaceous distal ends; tibiae testaceous with metallic green reflections; tarsomeres brown with metallic violet reflections; white, appressed setae on front and middle coxae, and laterally on hind coxae; erect setae and suberect closely spaced in several regular and irregular rows on all femora; setae widely spaced in a few rows on all tibiae; middle tibiae with patch of appressed setae dorsally along distal half; tarsomeres with short scattered setae on ventral surface; distal tarsomeres with two asymmetrical rows each with a few to several small, erect setae; an erect subapical seta present only on front trochanter, absent on middle and hind trochanters; males with dense pad of erect setae ventrally on proximal three tarsal segments; tarsal claws small.

*Elytra.* Figs 3A, 4A. Form narrow in male, broadened distad and broadest at outer apical angle in female; evenly curved along posterior margins with apices separately rounded, more pronounced in female; sutural spine small in female, indistinct in male, feebly withdrawn from apex; posterior margins finely microserrulate. Surface finely granulate, impunctate, numerous small, irregular, shiny green or blue green flecks of various sizes scattered over a dull, dark copper brown background; fully developed elytral pattern of narrow, white markings contrasting with the darker elytral ground color; setigerous punctures with short, erect, transparent setae indistinct in subsutural rows on disc, but distinct at elytral base, and at inner humeral angles, each surrounded by a metallic fleck slightly larger than flecks elsewhere on elytra; surface slightly depressed in humeral area and on disc creating a slight but distinct raised area basally. Elytral markings white forming an broken pattern consisting of partial humeral, complete apical lunules and middle band; humeral lunule reduced to its extremities, discal spot as a small, circular dot; middle band complete, narrow, terminating near the suture in a broad hook only slightly enlarged in most specimens without the posterior end recurved anteriorly, or the lateral end expanded along lateral margin; apical lunule complete and narrow along entire elytral apex to suture. Elytral epipleura testaceous except for narrow, metallic green to copper green band along dorsal margin.

*Abdomen.* Figs 4B, 4E. Surface of 1st-5th sterna shiny black with green reflections, sterna 6 entirely shiny black to black brown; posterior margins of male 3rd-5th sterna and female 3rd-4th sterna narrowly black; posterior margin female 5th sternum broadly black; 3rd-5th sterna medially smooth with scattered, fine, erect setae in both sexes; male 1st-6th sterna and female 1st-5th sterna laterally covered with dense, scattered, appressed white setae and roughened from setal punctures; male 6th sternum glabrous medially with a broad, deep concave notch; female 5th sternum with slightly raised transverse wrinkles interrupted by a short, wide membranous band along midline extending anteriorly to middle of sternum from a wide transverse membranous wedge along posterior margin; female 6th sternum entirely glabrous, posterior margin with a row of 6-10 erect spines and a small lateral gibbosity on each side.

*Male Genitalia.* Figs 3E, 3F, 3G. Shape wide near base, uniformly broad to short wide distal neck, apical hook abruptly rounded, tip long and at acute angle to aedeagus; aedeagus inner sac apical spine field in neck short and wide, forming a distinct pad. Aedeagus inner sac sclerites: stylet tip recurved forming a sharp point; shield rounded distad; large tooth short and rounded at tip with root and dark fields large; arched piece short and thin.

#### Ecology.

The exact locality, and hence the habitat, where Darlington collected this new species is uncertain. Darlington's labels on his 1934-collecting trip to the Massif de la Selle indicate that he first collected south of Port-au-Prince from “Kenskoff [Kenscoff] to La Visite and vicinity” and then later on his return trip along an “auto road” from Furcy to Kenskoff [Kenscoff]. The auto road he took is most likely the same as current Highway 101 that traverses the Massif de la Selle from Port-au-Prince to near the southern coast of Haiti. Suitable habitats where Darlington collected likely occurred along this road at elevations of roughly 5000 feet. An image from Google Earth© (Fig. 18E), and a geotagged image from http://flickr.com taken in early 2010 along Highway 101 below its summit in the vicinity of Parc de La Visite (Fig. 18F), both show an unimproved rocky road cut into a steep slope with sparse vegetation. The road banks and roadway appear eroded and traversed mostly on foot by local inhabitants. In Parc de La Visite itself, this highway reaches 6000 feet elevations, becomes much more level, and runs through stands of *Pinus occidentalis* Swartz with grassy openings of *Andropogon* sp. within the pine forest (Fig. 18E). Darlington (1935) notes that his base camp for collecting at La Visite was in “tall pine forest at 6,000 feet beside the Rivière Chotard” [currently known as Cascade de Seguin]. However, he states that his collecting within the pine stands produced no carabids. Thus, the habitat where Darlington collected this new species is likely situated along the road he took to reach Parc de La Visite, but at a lower elevation than where he camped. The site in Fig. 18F with eroded clay banks along Highway 101 at about 1755 m (5760 ft) has been selected as the type locality for this new species. The only known specimens of this new species were collected during September.

#### Distribution.

Fig. 22. HAITI: Département du l'Ouest, Massif de la Selle. Kenskoff [Kenscoff], Furcy, and La Visite are localities on the labels of the type specimens collected by P.J. Darlington in 1934. This species likely occurs in suitable habitats from Kenscoff to the northwestern boundary of Parc de La Visite along Highway 101. This highway from Furcy southward to La Visite traverses a series of long ridges at about 5000 feet elevation before rising abruptly to 6000 feet where it crosses over to the southern slopes of the Massif de la Selle.

#### Etymology.

This Latinized eponym, genitive case, is based on the family name of the late Philip J. Darlington, Museum of Comparative Zoology, Harvard University, Cambridge, Massachusetts. The species name acknowledges his contributions as the preeminent American carabidologist during the Mid-20th Century. During his 1934 expedition to Haiti, Darlington was the first coleopterist to collect in the Massif de la Selle, the highest mountains in Haiti. Darlington collected the types of this new species along the autoroute taken by his expedition across the Massif de la Selle in the vicinity of La Visite, now designated as Parc de La Visite, a National Park in this southeastern Haitian mountain range.

#### Remarks.

This species is most closely related to *Brasiella davidsoni* from the Massif de la Hotte on the Tiburon Peninsula of western Haiti ca. 185 km to the west of the Massif de la Selle. The four typical sclerites within the inner sac of the aedeagus that characterize *Brasiella* are very similar for both species especially the unusual form of the recurved terminal tip of the stylet, although this differs in the degree of its curvature for each species. Females of both species possess similar small, paired mediolateral gibbosities on the 6th abdominal sternum. Moreover, the elytral markings of both species are quite similar in pattern with only the extremities of the humeral lunule evident; this marking on all specimens examined consistently was divided into a humeral mark and a discal spot variously reduced or enlarged depending on the specimen. Additionally, the middle lunule always terminates near the suture in a broad hook never anteriorly recurved. However, the distinctiveness of these two species is confirmed by differences between the two in the size of the recurved aedeagus apex of males, as well as, differences in the extent of development of the membranous longitudinal median band and membranous wedge on the 5th abdominal sternum of females.

### Brasiella davidsoni sp. n.

urn:lsid:zoobank.org:act:1BFF492B-912D-4B09-8240-1BD99E02969C

http://species-id.net/wiki/Brasiella_davidsoni

Figs 5 6

#### Holotype.

Male! labeled “HAITI: Departement / du Sud, Ville Formon / 31 km NW Les Cayes, / S slope Morne Formon / Massif de La Hotte” [typeset black on white label]; “18-20N, 74-01W / 1405m, 7-8 Sept 1995 / R.Davidson, G.Onore / J.Rawlins. Disturbed / forest and fields” [typeset black on white label]; “Carnegie Museum / Specimen Number / CMNH-500,316” [typeset black on white label]; “HOLOTYPE / Brasiella / davidsoni / Acciavatti” [typeset black on red label]. [Genitalia in glycerin in a microvial pinned beneath specimen.]

#### Allotype.

Female! labeled with the same locality data as the holotype; “Carnegie Museum / Specimen Number / CMNH-494,052” [typeset black on white label]; “ALLOTYPE / Brasiella / davidsoni / Acciavatti” [typeset black on red label].

#### Paratypes.

Specimens! as follows: 1) 95 males and 64 females labeled with the same locality data as the holotype; “PARATYPE / Brasiella / davidsoni / Acciavatti” [typeset black on blue label]; [these paratypes each labeled with a CMNH Unique Number]; 2) 3 males and 3 females labeled “Desbarriere / Mf.LaHotte / nr. 4000 ft. / Oct. 12-14” [typeset black on white label]; “Haiti / 1934 / Darlington” [typeset black on white label]; “C. (Brasiella) / dominicana Mandl / det. R. Freitag / April 1988” [typeset black on white label]. “PARATYPE / Brasiella / davidsoni / Acciavatti” [typeset black on blue label].

#### Type Depositories.

Holotype, allotype and 157 paratypes at CMNH; each CMNH Unique Number stored in data files at CMNH. Paratypes (4 each sex) at MCZH.

[Male paratype from Desbarriere at CMNH with Carnegie Museum Specimen Numbers: CMNH-495,489. Female paratype from Desbarriere at CMNH with Carnegie Museum Specimen Numbers: CMNH-524,004. Male paratypes from Ville Formon at MCZH with Carnegie Museum Specimen Numbers: CMNH-529,026; 519,749. Female paratype from Ville Formon at MCZH with Carnegie Museum Specimen Numbers: CMNH-514,359; 521,194.]

#### Type Locality.

HAITI: Département du l'Sud, Ville Formon, Morne Formon, Massif de La Hotte, 18°20'N, 74°01'W, 1405 m. Aerial view in Fig. 19A.

#### Diagnosis.

Distinguished from other *Brasiella* species on Hispaniola by the following combination of characters: 1) elytral markings exhibiting a humeral lunule reduced to its extremities; middle lunule terminating near the suture in a broad hook, never anteriorly recurved; apical lunule entire or broken into marginal band and subapical dot; 2) eyes small, neither prominent nor bulging laterally; 3) male genitalia with aedeagus neck broadly and distinctly bowed, its apex terminating in an extremely short, blunt curved tip; 4) male aedeagus inner sac stylet with distal apex distinctively hooked at tip; 5) females completely lack the longitudinal membranous band and membranous wedge mesad on the female 5th abdominal sternum developed to various degrees in the other *Brasiella* species on Hispaniola.

#### Description.

*General.* Fig.s 5A, 6A. *Body.* Formslender; head wide, eyes small, neither prominent nor bulging laterally; pronotum square, sligthly wider than long; elytra in both sexes narrow only slightly wider distad, apices separately rounded. *Size*.Males, length 5.6-5.8 mm, width 1.7-1.8 mm; females, length 6.2-6.5 mm, width 1.8-1.9 mm.

*Head*. Fig.s 5B, 5D, 6D, 6F. Shiny dark copper black, back green or violet green dorsally and black green ventrally; entire surface glabrous except for two pairs of supraorbital sensory setae. Frons finely and longitudinally rugose. Vertex more coarsely rugose, transverse rugae along anterior margin narrow and irregularly arranged, 11-14 more or less complete longitudinal rugae between eyes and middle where rugae converge into an arcuate pattern; rugae transition abruptly into a posterior area with a finely and irregularly granulate surface. Eyes small, neither prominent nor bulging laterally. Genae longitudinally rugose. Clypeus finely and irregularly granulate, narrowed mesad. Labrum testaceous with a dark brown margin, subrectangular, width to length ratio 3.1 in holotype male, ratio 2.4 in allotype female; anterior margin protruding broadly mesad with a small medial tooth, both protruded margin and tooth larger in female; posterior margin very broadly arcuate mesad; medial carina broadly raised, very slight depression on each side near posterior margin; 6-7 setae in an irregular row near middle, most often symmetrically arranged. Maxillae and labium mainly testaceous, only distal palpal segments dark brown with metallic blue green reflections. Mandibles sexually dimorphic; in male, surface mainly testaceous, only teeth shiny brown with metallic green; in female, surface only testaceous in basal half, apical half and teeth shiny brown with metallic green; mandibles symmetrical, four teeth distad of molar, apical tooth longest, first and third tooth coequal in length, second tooth shortest; gaps between three intermediate teeth wide in both sexes. Antennae 11 segmented; scape dorsally shiny green, ventrally testaceous with a single subapical sensory seta; antennomeres 2-4 shiny green, glabrous except for a few, short erect setae along their length and distally; antennomeres 5-11 dull black, sheathed with dense short sensory setae.

**Figure 5.**
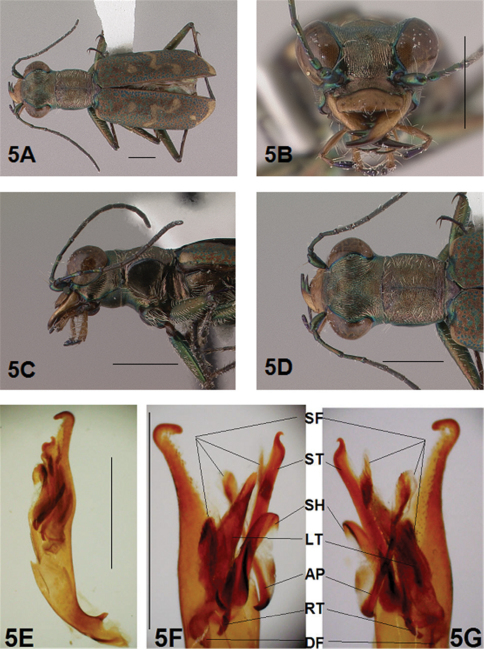
*Brasiella davidsoni*, sp. n., male. Holotype **5A** Body, dorsal **5B** Head, anterior **5C** Body, anterior, left lateral **5D** Body, anterior, dorsal **5E** Aedeagus, dorsal **5F** Aedeagus inner sac, ventral aspect, and **5G** Aedeagus inner sac, dorsal aspect–AP, arched piece; LT, large tooth; SH, shield; ST, stylet; SF, spine fields (one displaced from within aedeagus neck and two isolated); RT, root of LT; DF, dark fields. [Scale lines = 1 mm].

**Figure 6.**
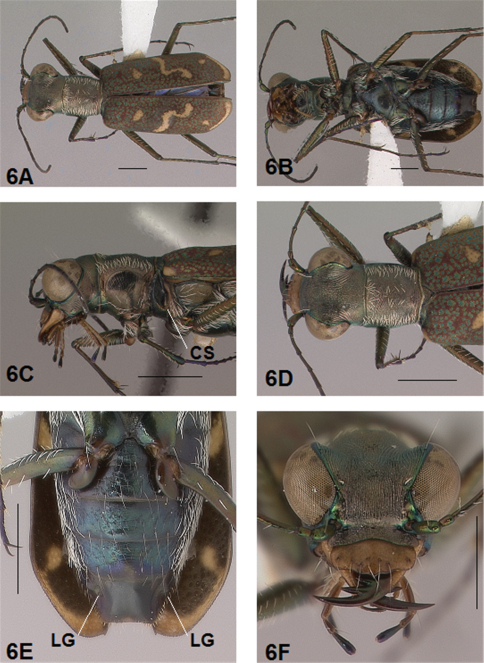
*Brasiella davidsoni*, sp. n., female. Allotype **6A** Body, dorsal **6B** Body, ventral **6C** Body, anterior, left lateral–CS, coupling sulcus **6D** Body, anterior, dorsal **6E** Abdomen, sterna, ventral–6^th^ sternum, LG, lateral gibbosity **6F** Head, anterior. [Scale lines = 1 mm].

*Prothorax.* Figs 5C, 5D, 6C, 6D. Pronotum shiny, dark copper black. Proepisterna shiny, dark copper black, surface wrinkled dorsad. Prosternum shiny, dark copper black, surface wrinkled dorsad. Pronotum glabrous except for short, decumbent, white setae distributed in several, irregular rows medially directed, originating close to, and lying in a narrow band distinctly removed from lateral suture, in a sparse narrow band transversely and anteriorly oriented within broad anterior margin, and in a sparse narrow band laterally oriented on each side of midline extending nearly to the narrow posterior margin; transverse submarginal sulci distinct, anterior sulcus shallow, posterior sulcus deeper and deepest at posterior angles; transverse rugae within broad anterior margin irregular and shallow, interrupted at middle by an irregularly arranged pattern, within narrow posterior margin more distinctly and deeply engraved especially medially and extending onto midline; surface sculptured by fine, transverse rugae angled on disc and interrupted by a finely engraved longitudinal midline, and more finely and irregularly sculptured elsewhere. Proepisterna glabrous except for white, erect and appressed setae arising from small setigerous punctures scattered over ventral and posterior surfaces in males, only in ventral half in females. Prosternum glabrous, surface smooth.

*Pterothorax.* Figs 5C, 6C. Mesepisterna glabrous except for appressed setae near ventral margin; female coupling sulcus represented by a small, shallow depression medially situated, a distinct groove extends only dorsally from pit, surface smooth below pit. Mesepimeron with sparse appressed setae. Metepisterna with scattered, appressed setae, more abundant in male than female. Prosternum and mesosternum glabrous, smooth to slightly wrinkled; metasternum glabrous except for long, dense white appressed setae laterad, surface smooth mesad and coarsely sculpted laterad where setae originate. Scutellum triangular, copper black.

*Legs.* Figs 5A, 6B. Segmentstestaceous brown with metallic brown green reflections. Coxae shiny metallic brown green; trochanters shiny testaceous; femora and tibiae testaceous with metallic green reflections anteriorly; tarsomeres dark metallic brown; white, appressed setae on front and middle coxae, and laterally on hind coxae; erect setae and suberect closely spaced in several regular and irregular rows on all femora; setae widely spaced in a few rows on all tibiae; middle tibiae with patch of appressed setae dorsally along distal half; tarsomeres with short scattered setae on ventral surface; distal tarsomeres with two asymmetrical rows each with a few to several small, erect setae; an erect subapical seta present only on front trochanter, absent on middle and hind trochanters; males with dense pad of erect setae ventrally on proximal three tarsal segments; tarsal claws small.

*Elytra.* Figs 5A, 6A. Form narrow in male, only slightly wider distad in female; posterior margins rounded, apices more evenly curved and separately rounded in female; sutural spine tiny, feebly withdrawn from apex; posterior margins finely microserrulate. Surface finely granulate, impunctate, numerous small, irregular, shiny green or blue green flecks of various sizes scattered over a dull, dark copper brown background; elytral pattern broad, broken, contrasting with the darker elytral ground color; setigerous punctures with short, erect, transparent setae indistinct in subsutural rows on disc, but distinct at elytral base, and at inner humeral angles, each surrounded by a metallic fleck slightly larger than flecks elsewhere on elytra; surface slightly depressed in humeral area and on disc creating a slight but distinct raised area basally. Elytral markings tawny, reduced pattern consisting of four or five isolated markings; humeral lunule broken into two widely separated terminal dots, humeral angle spot very small, discal spot small and oval; middle band sinuate, uniformly wide, not expanded laterally nor recurved anteriorly near suture; apical lunule entire or narrowly broken into marginal band and subapical dot. Elytral epipleura testaceous except for narrow, metallic green to copper green band along dorsal margin.

*Abdomen.* Figs 6B, 6E. Surface of 1st-5th sterna shiny black with green reflections, sterna 6th entirely shiny black to black brown; posterior margins of male 3rd-5th sterna and female 3rd-4th sterna narrowly black; posterior margin female 5th sternum broadly black; 3rd-5th sterna medially smooth with scattered, fine, erect setae in both sexes; male 1st-6th sterna and female 1st-5th sterna laterally covered with dense, scattered, appressed white setae and roughened from setal punctures; male 6th sternum glabrous medially with a broad, deep concave notch; female 5th sternum with slightly raised transverse wrinkles, longitudinal membranous band and membranous wedge absent; female 6th sternum entirely glabrous, posterior margin with a row of 6-10 erect spines and a small lateral gibbosity on each side.

*Male Genitalia.* Figs 5E, 5F, 5G. Shape broad near base, gradually tapering and uniformly broad to long, broad distal neck evenly curved, its apex terminating in an extremely short, blunt curved tip. Aedeagus inner sac sclerites: stylet long and straight, small hook at terminal tip; shield tapered distad; large tooth long and pointed at tip with large root and large dark fields; arched piece long and broad at base; spine field within aedeagus neck short and narrow.

#### Ecology.

Robert L. Davidson informed the author that this new species, named in his honor, was extremely abundant at the type locality when he and other CMNH staff collected there in early September. This new species was collected along dirt roads and trails through fields and disturbed forests at 1405 m on the south slope of Morne Formon in the Massif de la Hotte (Fig. 19A). Darlington's specimen labels indicate he collected this new species at about the same elevation around the paratype locality of Desbarriere in mid-October. Darlington (1935) during his Haitian Expedition traversed treacherous terrain at Desbarriere where vegetation covered eroded limestone slopes on the north side of the Massif de la Hotte. From the collection data available, it appears that adults of this new species are active during the latter part of the year in September and October.

#### Distribution.

Fig. 22. HAITI: Département du l'Sud, Morne Formon, and Département du Grand'Anse, Desbarriere, in the Massif de la Hotte on the Tiburon Peninsula of western Haiti. This species is likely to be encountered in suitable habitats at the higher elevations throughout the Massif de la Hotte.

#### Etymology.

This Latinized eponym, genitive case, is based on the family name of Robert L. Davidson, Carnegie Museum of Natural History, Pittsburgh, Pennsylvania. Robert is a recognized taxonomic expert on Carabidae and, for several decades, has been my mentor for studying these beetles and a close friend. I am honored to name this species for him, not only because he collected this new species, but also for his outstanding efforts to acquire specimens of several other new *Brasiella* species described in this revision. Although Philip J. Darlington collected specimens of this new species in the Massif de la Hotte during his 1934 expedition to Haiti, earlier workers did not consider these to be distinct from either *Brasiella argentata* (Fabricius) or *Brasiella dominicana* (Mandl). The conspecificity of the Darlington specimens with *Brasiella davidsoni* was established in this revision. The Darlington specimens fall within the range of variation exhibited by this new species as documented by the large series of specimens collected by Robert L. Davidson and CMNH colleagues.

#### Remarks.

This species appears closely related to *Brasiella darlingtoniana* from the Massif de la Selle situated farther eastward in central Haiti (refer to discussion under that species) because of the suite of unique synapomorhic characters the two share that are not found in other *Brasiella* on Hispaniola. The shape and form of the male aedeagus and the four typical sclerites within the inner sac of the aedeagus that characterize *Brasiella* are the most highly modified in this species compared to others on Hispaniola. Among these modifications, the aedeagus neck is broadly and distinctly recurved with an apex terminating in an extremely short, blunt end. Furthermore, the inner sac of the male genitalia has a stylet with a distinctively hooked terminal tip. For females, the membranous transverse wedge and longitudinal median band on the 5th abdominal sternum, fully or partially developed in the other *Brasiella* species on Hispaniola, are completely absent. These modified morphological characters of this species, unique among the *Brasiella* on Hispaniola, were not previously considered in earlier phylogenetic analyses (Freitag and Barnes 1989). Because of this omission, it is unclear how to interpret their conclusions about phylogeny within the *Brasiella viridicollis* Dejean species group, a concept now greatly expanded by the addition of numerous species described in this revision. Thus, the relationship of *Brasiella davidsoni* to the other *Brasiella* in the Dominican Republic remains uncertain. Loss or modification of several characters by *Brasiella davidsoni* of homologous characters found in related species implies isolation for a long period of time leading to these character divergences.

### Brasiella dominicana

(Mandl, 1983)

http://species-id.net/wiki/Brasiella_dominicana

Figs 7 8

Cicindela (Brasiella) dominicana Mandl, 1983: 117
Brasiella dominicana (Mandl); Wiesner, 1992: 205
Brasiella dominicana (Mandl); Erwin and Pearson, 2008: 109

#### Holotype.

Male! labeled “Dominikenische Republik / Bani / leg. Klapperich 5,9.1971” [typeset black on white label]; “HOLOTYPUS” [black typewritten on red label glued onto white label]; “HOLOTYPUS / Cic. (Brasiella) / dominicana m. / Dr. K. MANDL det. 1980 2” [first three lines handprinted, fourth line typeset except numeral ‘2' handprinted, first line in red ink, other lines black ink]; “Cicindela / Brasiliella) / nova species aus / der Dominikeni- / sche Republick” [handscript blue ink on white label folded in half]; “709” [handprinted black on white]; “Cic. (Brasiella) / dominicana / 291 Mandl” [handscript black letters, red numerals, on white label]. “Brasiella ♂ / dominicana (Mandl) / det.R.E.Acciavatti” [handprinted and typeset black on white label]. [Holotype specimen and its extracted aedeagus both glued to the same card.]

#### Allotype.

Mandl designated no allotype for this species (Mandl 1983).

#### Paratypes.

Specimens! as follows: 1) 4 males and 1 female labeled with the same locality and collection data on the same style label as the holotype; additional labels as “PARATYPUS” [typewritten black on red label] and /or “PARATYPUS / Cic.(Brasiella) / dominicana nov. / Dr.K.Mandl det.1982” [typeset black on red label] and /or “PARATYPUS / Cic. (Brasiella) / dominicana m. / Dr. K. MANDL det. 1980 2” [first three lines handprinted, fourth line typeset except numeral 2 handprinted, first line in red ink, other lines black ink]; “Brasiella / dominicana (Mandl) / det. R.E.Acciavatti” [handprinted and typeset black on white label, ♂ or ♀ symbol added to first line as appropriate]; 2) 1 female labeled with the same locality data as the holotype, but collection date “22,8.1971” [22 August 1971]. [Five paratypes at CMNH each also labeled with a CMNH Unique Number on file].

#### Type Depositories.

Holotype, paratype (1 male) at NHMW; 5 paratypes (3 males, 2 females) at CMNH; each CMNH specimen with a Unique Number stored in data files at CMNH. Additional paratypes likely deposited at NHMW were not examined, nor were other paratypes known to have been distributed to colleagues elsewhere (Freitag 1992) after Mandl described this species.

#### Type Locality.

DOMINICAN REPUBLIC: Peravia Province, Bani, 18°16'51"N, 70°19'28"W at 59 m. Aerial view in Fig. 19B.

#### Notes on Type Locality.

The coordinates shown above were obtained from Google© and hereby establish the type locality of this species as inferred from the locality data on the types and from the original description. Bani is a town situated on the coastal plain where the Cordillera Central terminate along the southcentral coast of the Dominican Republic. Exactly where the type series for this species originated previously has been uncertain as Mandl did not present a specific locality other than Bani nor have any other specimens of this species been collected subsequent to the original type series. However, refer to the discussion under its Ecology below for circumstantial evidence as to the most likely collection site to help establish the type locality for this species at the coordinates shown above.

#### Diagnosis.

Distinguished from other *Brasiella* species on Hispaniola by the following combination of characters: 1) head, pronotum and proepisterna shiny dark copper; 2) elytral pattern complete, markings broad, bold, cream to pale white, contrasting with the darker copper brown and blue green flecked elytral ground color; 3) posterior elytral margins obliquely curved with apices separately rounded and sutural spine distinctly not retracted (more pronounced in females); 4) pronotum slender, narrower than long; 5) head and prontoum shiny dark copper brown contrasting with duller elytral ground color; 6) legs with large testaceous regions, especially the femora of females; 7) male genitalia with a long aedeagus neck and a short apical hook; 8) aedeagus apical spine field forming a long and narrow pad; 9) aedeagus inner sac stylet long, tip slightly bent and evenly tapered to a narrow point; 10) female 5^th^ abdominal sternum with with transverse wrinkles, a wide membranous band at midline along most the sternum, and a large membranous wedge along posterior margin.

#### Redescription.

**Figure 7.**
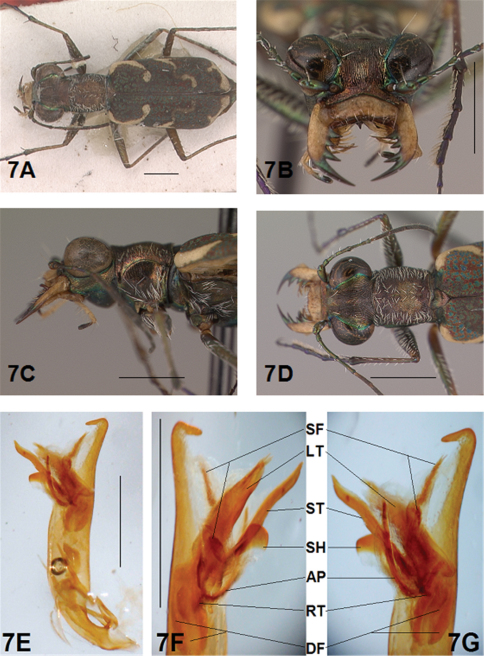
*Brasiella dominicana* (Mandl), male. Holotype **7A** Body, dorsal. Paratype **7B** Head, anterior **7C** Body, anterior, left lateral **7D** Body, anterior, dorsal **7E** Aedeagus, dorsal **7F** Aedeagus inner sac, ventral aspect, and **7G** Aedeagus inner sac, dorsal aspect–AP, arched piece; LT, large tooth; SH, shield; ST, stylet; SF, spine fields (one displaced from within aedeagus neck and two isolated); RT, root of LT; DF, dark fields. [Scale lines = 1 mm].

**Figure 8.**
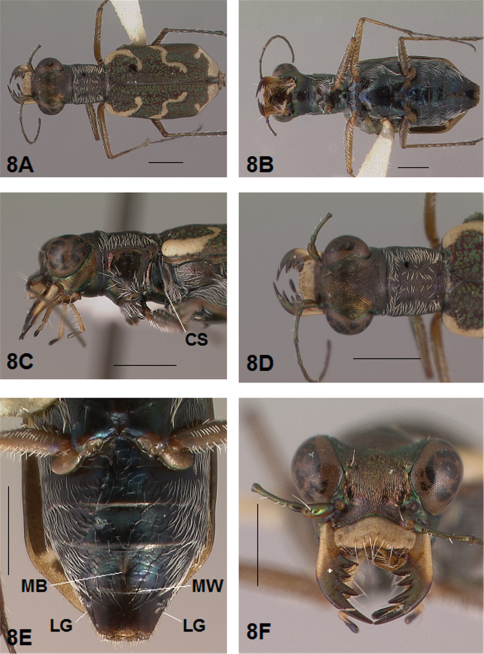
*Brasiella dominicana* (Mandl), female. Paratype **8A** Body, dorsal **8B** Body, ventral **8C** Body, anterior, left lateral–CS, coupling sulcus **8D** Body, anterior, dorsal **8E** Abdomen, sterna, ventral–5^th^ sternum, MB, membranous band; MW, membranous wedge; 6^th^ sternum, LG, lateral gibbosity **8F** Head, anterior. [Scale lines = 1 mm].

*General.* Figs 7A, 8A. *Body.* Formelongate; head narrow, eyes prominent, slightly bulging laterally; pronotum slender, narrower than long; elytra broadened distad, apices separately rounded. *Size*.Males, length 6.0-6.2 mm, width 1.9-2.0 mm; females, length 6.5-6.6 mm, width 2.1-2.2 mm.

*Head*. Figs 7B, 7D, 8D, 8F. Shiny dark copper brown dorsally and copper blue green ventrally; entire surface glabrous except for two pairs of supraorbital sensory setae. Frons finely and longitudinally rugose. Vertex more coarsely rugose, transverse rugae along anterior margin narrow and irregularly arranged, 15-18 more or less complete longitudinal rugae between eyes and middle where rugae remain parallel or only slightly converge into an arcuate pattern; rugae transition abruptly into a posterior area with a finely and irregularly granulate surface. Eyes prominent in both sexes, slightly bulging laterally, more in male than female. Genae longitudinally rugose. Clypeus finely and irregularly granulate, narrowed mesad. Labrum testaceous with a dark brown margin, subrectangular, width to length ratio 3.5 in holotype male, ratio 2.6 in allotype female; anterior margin sinuate, prominent at middle, a small bulge on either side of a tiny tooth, sinuation and tooth smaller in male, larger more distinctly sinuate in female; posterior margin distinctly arcuate mesad; medial carina broadly and distinctly raised; 6-8 setae in an irregular row near middle most often symmetrically arranged. Maxillae and labium mainly testaceous, only distal palpal segments dark brown with metallic blue green reflections. Mandibles sexually dimorphic; in male, surface mainly testaceous, only teeth metallic green; in female, surface only testaceous in basal half, apical half and teeth shiny brown; mandibles symmetrical, four teeth distad of molar, apical tooth longest, third and first tooth coequal in length, second tooth shortest; gaps between three intermediate teeth narrow in male, wide in female. Antennae 11 segmented; scape dorsally shiny green, ventrally testaceous with a single subapical sensory seta; antennomeres 2-4 shiny green, glabrous except for a few, short erect setae along their length and distally; antennomeres 5-11 dull brown, sheathed with dense short sensory setae.

*Prothorax.* Figs 7C, 7D, 8C, 8D. Pronotum shiny, dark copper brown. Proepisterna shiny, dark copper brown, surface wrinkled dorsad. Prosternum shiny green. Pronotum glabrous except for short, decumbent, white setae distributed in several, irregular rows medially directed, originating close to, and lying in a narrow band impinging on lateral suture, in a sparse narrow band transversely and anteriorly oriented within broad anterior margin, and in a sparse narrow band laterally oriented on each side of midline extending nearly to the narrow posterior margin; transverse submarginal sulci distinct, anterior sulcus shallow, posterior sulcus deeper and deepest at posterior angles; transverse rugae within broad anterior margin irregular and shallow, interrupted at middle by an irregularly arranged pattern, within posterior margin more distinctly and deeply engraved especially medially and extending onto midline; surface sculptured by fine, transverse rugae angled on disc and interrupted by a finely engraved longitudinal midline, and more finely and irregularly sculptured elsewhere. Proepisterna glabrous except for white, erect and appressed setae arising from small setigerous punctures scattered over most of the surface in males, only in ventral half in females. Prosternum glabrous, surface smooth.

*Pterothorax.* Figs 7C, 8C. Mesepisterna glabrous except for appressed setae near ventral margin; female coupling sulcus represented by a small, circular depression medially situated, a distinct groove extends only dorsally from pit, surface smooth below pit. Mesepimeron with sparse appressed setae. Metepisterna with scattered appressed setae, more abundant in male than female. Prosternum and mesosternum glabrous, smooth to slightly wrinkled; metasternum glabrous except for long, dense white appressed setae laterad, surface smooth mesad and coarsely sculpted laterad where setae originate. Scutellum triangular, cupreous.

*Legs.* Figs 7A, 8B. Segmentstestaceous brown with metallic brown green reflections. Coxae shiny metallic brown green; trochanters shiny testaceous; femora and tibiae testaceous with metallic green reflections anteriorly; tarsomeres dark metallic brown; white, appressed setae on front and middle coxae, and laterally on hind coxae; erect setae and suberect closely spaced in several regular and irregular rows on all femora; setae widely spaced in a few rows on all tibiae; middle tibiae with patch of appressed setae dorsally along distal half; tarsomeres with short scattered setae on ventral surface; distal tarsomeres with two asymmetrical rows each with a few to several small, erect setae; an erect subapical seta present only on front trochanter, absent on middle and hind trochanters; males with dense pad of erect setae ventrally on proximal three tarsal segments; tarsal claws small.

*Elytra.* Figs 7A, 8A. Form broadened distad and broadest at outer apical angle in both sexes; obliquely curved along posterior margins with apices separately rounded; sutural spine strongly withdrawn from apex (more pronounced in females); posterior margins finely microserrulate. Surface finely granulate, impunctate, numerous small, irregular, shiny green or blue green flecks of various sizes scattered over a dull, dark copper brown background; fully developed elytral pattern of broad, bold markings contrasting with the darker elytral ground color; setigerous punctures with short, erect, transparent setae indistinct in subsutural rows on disc, but distinct at elytral base, and at inner humeral angles, each surrounded by a metallic fleck slightly larger than flecks elsewhere on elytra; surface slightly depressed in humeral area and on disc creating a slight but distinct raised area basally. Elytral markings cream to pale white, bold and distinct, forming a complete pattern consisting of humeral and apical lunules and middle band; humeral lunule complete terminating as a slightly enlarged end on disc in most specimens, slightly broken at posterior end in a few specimens; middle band slightly sinuate, complete in all specimens examined, slightly enlarged near suture and slightly expanded along lateral margin; apical lunule wide, complete and broadened along suture in all specimens examined. Elytral epipleura testaceous except for narrow, metallic green to copper green band along dorsal margin.

*Abdomen.* Figs 8B, 8E. Surface of 1st-5th sterna shiny black with green reflections, 6th sternum entirely shiny black to black brown; posterior margins of male 3rd-5th sterna and female 3rd-4th sterna narrowly black; posterior margin female 5th sternum broadly black; sterna 3-5 medially smooth with scattered, fine, erect setae in both sexes; male 1st-6th sterna and female 1st-5th sterna laterally covered with dense, scattered, appressed white setae and roughened from setal punctures; male 6th sternum glabrous medially with a broad, deep concave notch; female 5th sternum with moderately raised transverse wrinkles, a wide membranous band at midline extending anteriorly along most of the sternum from a large membranous wedge along posterior margin; female 6th sternum entirely glabrous, posterior margin with a row of 6-10 erect spines and a small lateral gibbosity on each side.

*Male Genitalia.* Figs 7E, 7F, 7G. Shape narrow near base, uniformly broad in middle half, slim distally with neck long and narrow, apical hook abruptly rounded, tip shortened and acutely angled to aedeagus. Aedeagus inner sac sclerites: stylet tip long and bent; shield rounded distad; large tooth long, broad and pointed at tip with root and large dark fields; arched piece long and thin; spine field within aedeagus neck long and narrow.

#### Ecology.

As Mandl (1983) was not specific about the exact site where this species was collected, its habitat is uncertain. Nonetheless, the habitat of *Brasiella dominicana* can be inferred from its association with another tiger beetle, *Cicindela (Plectographa) schaefferi* W. Horn 1903. Both species were collected by Klapperich on the same dates at Bani based on the information presented in Mandl's publication and specimens examined at CMNH. *Cicindela schaefferi* occurs at other locations in the Dominican Republic at low elevations in riparian habitats based on specimens collected by Robert L. Davidson deposited at CMNH. Thus, it would appear that *Brasiella dominicana* also occurs in riparian habitats at low elevations, such as along the Rio Bani. This river originates in the southern end of the Cordillera Central and emerges from these mountains and flows along the eastern edge of Bani where Highway 2 crosses the Rio Bani (Fig. 19B). Additional inference about the collection site for *Brasiella dominicana* can be made from the better substantiated collection data for *Brasiella ocoa*, new species, which actually originated from a low elevation site along the southern coast of the Dominican Republic also in association with *Cicindela schaefferi* (refer to Ecology under that species account). These low elevation coastal plain habitats appear to be unusual for *Brasiella* species on Hispaniola based on ecological information provided by CMNH entomologist Robert L. Davidson. Davidson, who collected several of the new species of *Brasiella* described in this revision, reported encountering their adults only in the mountainous regions of Hispaniola. Because *Brasiella dominicana* occurs in low elevation habitats, it would appear that *Brasiella* species on Hispaniola actually occupy a wider habitat range than previously thought (Erwin and Pearson 2008). Adults of the type series of *Brasiella dominicana* were collected in August and September indicating an adult activity period during the later summer months.

#### Distribution.

Fig. 22. DOMINICAN REPUBLIC: Peravia Province, Bani vicinity along Rio Bani. The town of Bani lies in the coastal plain on the southcentral coast of the Dominican Republic. The Rio Bani emerges from the southeastern end of Cordillera Central and flows along the eastern edge of the town. This species likely occurs elsewhere along the Rio Bani further southward toward the coast and northward into the Cordillera Central.

#### Remarks.

*Brasiella dominicana* (Mandl) and *Brasiella ocoa*, new species, are the only species on Hispaniola with an elytral pattern of shiny, white markings with all lunules wide, complete and boldly contrasting with the darker, background color in most specimens examined. Despite this superficial similarity, there seems little doubt that these two species are distinctive based on differences in the form of the sclerites within the aedeagus of males of each species. A comparison of the other morphological characters presented in the key and the descriptions for each species further support their distinctiveness. These two species also appear to be allopatric based on the limited collection data currently available. However, additional collecting may change our understanding of their allopatry. Despite this distinctiveness as separate species and apparent allopatry, it is interesting to note that these two species seem to occupy a similar habitat type along the flood plains of major rivers flowing from the southeastern end of the Cordillera Central in Peravia Province, Dominican Republic. In these flood plain habitats, both species appear to have developed different habitat requirements from other species on Hispaniola. Their limited geographic distributions in close proximity to each other, and their similar requirements for low elevation habitats, may be the result of a common lineage.

### Brasiella iviei sp. n.

urn:lsid:zoobank.org:act:C972F17E-4FBA-481F-BB45-2C4105D4E348

http://species-id.net/wiki/Brasiella_iviei

Figs 9 10

#### Holotype.

Male! labeled “DOM.REP.Prov.Pedernales / ca.35 km N Cabo Rojo / LasAbejas, 1250m / 26AUG1988 / mixed forest, M.Ivie,Philips & Johnson” [typeset black on white label]; “HOLOTYPE / Brasiella / iviei / Acciavatti” [typeset black on red label]. [Genitalia in glycerin in a microvial pinned beneath specimen.]

#### Allotype.

Female! labeled “DOMIN.REP.:Pedernales / Province, Las Abejas / ca.35 km N Cabo Rojo / 26AUG-9SEP1988,1250m” [typeset black on white label]; “M.A.Ivie / T.K.Philips & K.A.Johnson” [typeset black on white label]; “Trap: / Flight Intercept“ [typeset black on white label]; Carnegie Museum / Specimen Number / CMNH-133,315” [typeset black on white label]; “ALLOTYPE / Brasiella / iviei / Acciavatti” [typeset black on red label].

#### Paratypes.

Specimens! as follows: 1) 1 female labeled with the same locality data format as the holotype; “PARATYPE / Brasiella / iviei / Acciavatti” [typeset black on blue label]; 2) 2 males and 1 female labeled with the same locality data format as the allotype; “PARATYPE / Brasiella / iviei / Acciavatti” [typeset black on blue label]. [Two of these paratypes (1 each sex) each labeled with Carnegie Museum Specimen Numbers: 133,315; 129,666]; 3) 5 females labeled “DOMIN.REP:Prov.Pedernales / ca.35km N Cabo Rojo, 1250m / LasAbejas, 26AUG-09SEP1988 / malaise trap, M.A.Ivie / T.K.Philips & K.A.Johnson” [typeset black on white label]; “PARATYPE / Brasiella / iviei / Acciavatti” [typeset black on blue label][Two of these paratypes each labeled with Carnegie Museum Specimen Numbers: 514,606; 130,216]; 4) 1 female labeled “DOMIN.REP:Prov.Pedern. / LasAbejas-ElAccitillar / ca.35km NNW Cabo Rojo / 23AUG1988, 1250-1430m” [typeset black on white label]; “M.A.Ivie, T.K.Philips / & K.A.Johnson” [typeset black on white label]; “PARATYPE / Brasiella / iviei / Acciavatti” [typeset black on blue label].

#### Type Depositories.

Holotype, 7 paratypes (1 male, 6 females) at WIBP; allotype and 3 paratypes (1 male, 2 females) at CMNH, each with a Unique Number stored in data files at CMNH.

#### Type Locality.

DOMINICAN REPUBLIC: Pedernales Province, Las Abejas, ca. 35 km N Cabo Rojo, 18°09'N, 71°38'W, 1250 m. An aerial view at this upper elevation along the southern slopes of the Sierra de Baoruco is shown in Fig. 20A.

#### Notes on Type Locality.

The label data on the type specimens do not provide coordinates for the site where the holotype originated. However, Las Abejas is a locality often visited by entomological expeditions to the Sierra de Baoruco, the mountains in the eastern area of the southernmost part of the Dominican Republic. The type locality is established here with coordinates shown above based on data published by Woodruff (2004) for Scarabaeidae specimens collected at Las Abejas by Robert L. Davidson and John E. Rawlins on their 1987 CMNH expedition to the Sierra de Baoruco, Dominican Republic. Fig. 20A depicts the ravines typically found at upper elevations on the southern slope of the Sierra de Baoruco; Las Abejas being one of the ravines accessible by a footpath from higher elevations (refer to Ecology below).

#### Diagnosis.

Distinguished from other *Brasiella* species on Hispaniola by the following combination of characters: 1) body size, males > 6.5 mm, females > 7.0 mm; 2) head longer than pronotum in female, similar in length in male; 3) eyes large, prominent and slightly bulging laterally; 4) head and pronotum shiny copper red, elytra dull cupreous to copper brown; 5) elytra with a complete pattern of pale, tawny markings in most specimens, especially females, or inconspicuous and obscured by ground color in others; 6) male genitalia apex short and wide, tip acutely bent and tapering gradually to an elongated point; 7) aedeagus inner sac stylet broad, tip recurved; 8) female 6th abdominal sternum lateral gibbosities small, posterior margin broadly rounded; 9) female 8th abdominal sternum median notch wide and deep.

#### Description.

**Figure 9.**
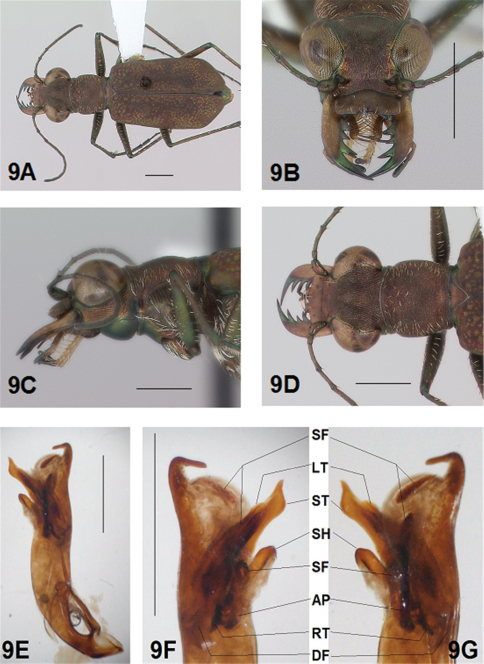
*Brasiella iviei*, sp. n., male. Holotype **9A** Body, dorsal **9B** Head, anterior **9C** Body, anterior, left lateral **9D** Body, anterior, dorsal **9E** Aedeagus, dorsal **9F** Aedeagus inner sac, ventral aspect, and **9G** Aedeagus inner sac, dorsal aspect–AP, arched piece; LT, large tooth; SH, shield; ST, stylet; SF, spine fields (one displaced from within aedeagus neck and two isolated); RT, root of LT; DF, dark fields. [Scale lines = 1 mm].

**Figure 10.**
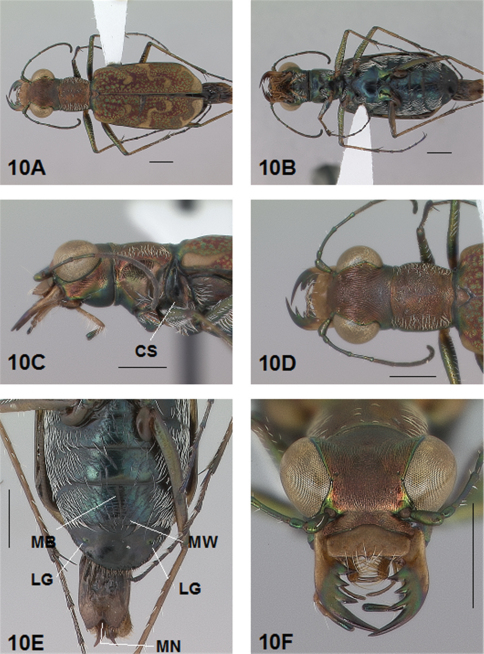
*Brasiella iviei*, sp. n., female. Allotype **10A** Body, dorsal **10B** Body, ventral **10C** Body, anterior, left lateral–CS, coupling sulcus **10D** Body, anterior, dorsal **10E** Abdomen, sterna, ventral–5^th^ sternum, MB, membranous band; 5^th^ sternum, MW, membranous wedge; 6^th^ sternum, LG, lateral gibbosity; 8^th^ sternum, MN, median notch **10F** Head, anterior. [Scale lines = 1 mm].

*General.* Figs 9A, 10A. *Body.* Formelongate; head long and broad, eyes large, prominent and slightly bulging; pronotum square; elytra elongate only sightly broadened distad, apices separately rounded. *Size*.Males, length 6.6-6.7 mm, width 1.9-2.0 mm; females, length 7.1-7.5 mm; width 2.1-2.2 mm.

*Head*. Figs 9B, 9D, 10D, 10F. Shiny copper red dorsally and copper blue green ventrally; entire surface glabrous except for two pairs of supraorbital sensory setae. Frons finely and longitudinally rugose. Vertex more coarsely rugose, transverse rugae along anterior margin narrow and irregularly arranged, 15-18 more or less complete longitudinal rugae between eyes and middle where rugae converge into an arcuate pattern; rugae transition abruptly into a posterior area with a finely and irregularly granulate surface. Eyes large, prominent and slightly bulging in both sexes. Genae longitudinally rugose. Clypeus finely and irregularly granulate, narrowed mesad. Labrum testaceous with a dark brown margin, subrectangular, width to length ratio 3.5 in holotype male, ratio 2.8 in allotype female; anterior margin in female slightly sinuate, protruding at middle with a small bulge on either side of a small tooth; anterior margin in male less sinuate, not protruding and tooth minute in some specimens or indented mesad in others; posterior margin broadly arcuate mesad; medial carina narrowly and distinctly raised, very slight depression on each side near posterior margin; 6-10 setae in an irregular row near middle most often symmetrically arranged. Maxillae and labium mainly testaceous, only distal palpal segments dark brown with metallic blue green reflections. Mandibles sexually dimorphic; in male, surface mainly testaceous, only teeth metallic green; in female, surface only testaceous in basal half, apical half and teeth shiny brown; mandibles symmetrical, four teeth distad of molar, apical tooth longest, first and third tooth coequal in length, second tooth shortest; gaps between three intermediate teeth narrow in male, wide in female. Antennae 11 segmented; scape dorsally shiny green, ventrally testaceous with a single subapical sensory seta; antennomeres 2-4 shiny green, glabrous except for a few, short erect setae along their length and distally; antennomeres 5-11 dull brown, sheathed with dense short sensory setae.

*Prothorax.* Figs 9C, 9D, 10C, 10D. Pronotum shiny copper. Proepisterna shiny, dark copper brown, surface wrinkled dorsad. Prosternum shiny green. Pronotum glabrous except for short, decumbent, white setae distributed in several, irregular rows medially directed, originating close to, and lying in a narrow band slightly impinging on lateral suture, in a sparse narrow band transversely and anteriorly oriented within broad anterior margin, and in a sparse narrow band laterally oriented on each side of midline extending nearly to the narrow posterior margin; transverse submarginal sulci distinct, anterior sulcus shallow, posterior sulcus deeper and deepest at posterior angles; transverse rugae within broad anterior margin irregular and shallow, interrupted at middle by an irregularly arranged pattern, within posterior margin more distinctly and deeply engraved especially medially and extending onto midline; surface sculptured by fine, transverse rugae angled on disc and interrupted by a finely engraved longitudinal midline, and more finely and irregularly sculptured elsewhere. Proepisterna glabrous except for white, erect and appressed setae arising from small, setigerous punctures scattered over ventral and posterior surfaces in males, only near ventral margin in females. Prosternum glabrous, surface wrinkled.

*Pterothorax.* Figs 9C, 10C. Mesepisterna glabrous except for appressed setae near ventral margin; female coupling sulcus represented by an elongate depression medially situated with a slightly deeper center, a distinct groove extends only dorsally from center, surface smooth below center. Mesepimeron with sparse appressed setae. Metepisterna with scattered appressed setae, more abundant in male than female. Prosternum and mesosternum glabrous, smooth to slightly wrinkled; metasternum glabrous except for long, dense white appressed setae laterad, surface smooth mesad and coarsely sculpted laterad where setae originate. Scutellum triangular, cupreous.

*Legs.* Figs 9A, 10B. Segmentstestaceous brown with metallic brown green reflections. Coxae shiny metallic brown green; trochanters shiny testaceous brown; femora and tibiae entirely metallic green to partially metallic green except for testaceous distal ends and posterior margin; tarsomeres dark metallic brown; white, appressed setae on front and middle coxae, and laterally on hind coxae; erect setae and suberect closely spaced in several regular and irregular rows on all femora; setae widely spaced in a few rows on all tibiae; middle tibiae with patch of appressed setae dorsally along distal half; tarsomeres with short scattered setae on ventral surface; distal tarsomeres with two asymmetrical rows each with a few to several small, erect setae; an erect subapical seta present only on front trochanter, absent on middle and hind trochanters; males with dense pad of erect setae ventrally on proximal three tarsal segments; tarsal claws small.

*Elytra.* Figs 9A, 10A. Form elongate, narrow in male, broadened distad and broadest at outer apical angle in female; obliquely curved along posterior margins with apices separately rounded; sutural spine feebly withdrawn from apex; posterior margins finely microserrulate. Surface finely granulate, impunctate, numerous small, irregular, shiny green to copper green flecks of various sizes scattered over a dull, cupreous to copper brown background; pale elytral markings barely contrasting with the darker elytral ground color in most marked female specimens, or obscured by ground color in others of both sexes; setigerous punctures with short, erect, transparent setae indistinct in subsutural rows on disc, but distinct at elytral base, and at inner humeral angles, each surrounded by a metallic fleck slightly larger than flecks elsewhere on elytra; surface slightly depressed in humeral area and on disc creating a slight but distinct raised area basally. Elytra marked with a complete pattern of pale, tawny lunules and bands in most specimens, especially females, or inconspicuous and obscured by ground color in others; complete pattern consisting of humeral and apical lunules and middle band, but pattern wide or thin or broken or obscured depending on the specimen; in marked specimens humeral lunule complete terminating on disc in a slightly enlarged end or isolated spot, middle band slightly sinuate and slightly enlarged near suture and slightly expanded along lateral margin; apical lunule wide, complete and broadened along suture. Elytral epipleura testaceous except for narrow, metallic green to copper green band along dorsal margin.

*Abdomen.* Figs 10B, 10E. Surface of 1st-5th sterna shiny black with green reflections, sterna 6th entirely shiny black to black brown; posterior margins of male 3rd-5th sterna and female 3rd-4th sterna narrowly black; posterior margin female 5th sternum broadly black; sterna 3rd-5th medially smooth with scattered, fine, erect setae in both sexes; male 1st-6th sterna and female 1st-5th sterna laterally covered with dense, scattered, appressed white setae and roughened from setal punctures; male 6th sternum glabrous medially with a broad, deep concave notch; female 5th sternum with moderately raised, transverse wrinkles interrupted by a membranous band along midline extending anteriorly from a short transverse membranous wedge along posterior margin; female 6th sternum entirely glabrous, posterior margin with a row of 6-10 erect spines and a small lateral gibbosity on each side.

*Male Genitalia.* Figs 9E, 9F, 9G. Shape narrow near base, gradually wider and uniformly broad along most of its length, distally ending in a short, wide apical neck; apical tip acutely bent and tapering evenly to an elongated point. Aedeagus inner sac sclerites: stylet broad, tip wide and recurved; shield rounded distad; large tooth short, bluntly rounded at tip with large root and dark fields; arched piece short and wide; spine field within aedeagus neck long and narrow.

#### Ecology.

The type series was collected at 1250 m within Las Abejas, a deep ravine abruptly descending from the forests of Hispaniolan Pine, *Pinus occidentalis* Swartz 1788, that cover the higher elevations on the southern slopes of the Sierra de Baoruco (Konstantinov and Chamorro-Lacayo 2006). The habitat at Las Abejas has a premontane, wet forest, rich in epiphytes (Fisher-Meerow and Judd 2008). As shown in Fig. 20A, these moist forests occur in ravines at upper elevations along the southern slopes of the Sierra de Baoruco. Fig. 20B depicts the highly erosive nature of the clay soils on these slopes, and Fig. 20C depicts the diverse nature of the vegetation along the footpath descending into the higher parts of Las Abejas. In these habitats, the adults of *Brasiella iviei* would appear to be rarely seen, and if they were seen, then to readily escape hand collecting by running into the vegetation. Indeed, nearly all the known adult specimens of this new species were collected with flight intercept or malaise traps. Fig. 20D shows a tropical malaise trap set up in an opening along a trail within the forest at Las Abejas during the 1987 CMNH expedition to the Sierra de Baoruco, Dominican Republic. Based on the type series of *Brasiella iviei* collected in August and September, this new species likely has its period of adult activity during the later summer months.

#### Distribution.

Fig. 22. DOMINICAN REPUBLIC: Pedernales Province, Las Abejas, ca. 35 km N Cabo Rojo, 1250 m, in moist ravines at higher elevations on the southern slopes of the Sierra de Baoruco.

#### Etymology.

This Latinized eponym, genitive case, is based on the family name of Michael A. Ivie, Department of Entomology, Montana State University, Bozeman, Montana. Ivie collected the first series of *Brasiella* specimens in 1988 from the Sierra de Baoruco, Pedernales Province, Dominican Republic, as part of his West Indian Beetle Fauna Project (WIBP). He previously considered these specimens to be only variants of *Brasiella dominicana* (Mandl). I am grateful for the opportunity he has provided for me to examine these WIBP specimens because it has led to the discovery of *Brasiella iviei*,and other related new species in the Sierra de Baoruco, an area in the Dominican Republic known for its high endemism (Woodruff 2004).

#### Remarks.

*Brasiella iviei* appears to be most closely related to *Brasiella rawlinsi*. However, their distinctiveness as separate species was established in this revision by differences presented in the key to their identification and under their descriptions. Adults of the former species are larger in body size with a proportionately longer head and pronotum than any known individuals of *Brasiella rawlinsi* in the Sierra de Baoruco. Also, elytral markings of *Brasiella iviei*, although still faint compared to other species on Hispaniola, are more obvious and wider than the majority of known *Brasiella rawlinsi* specimens. Males of these two species possess distinctive aedeagus inner sac sclerites, in particular, the form of the stylet. Additional differences between females of these two species are evident in the extent to which the membranous, longitudinal median band is developed on the 5th abdominal sternum, the size of the lateral gibbosities on the 6th abdominal sternum, along with the breadth and depth of the median notch on the 8^th^ abdominal sternum. In addition to these morphological differences between *Brasiella iviei* and *Brasiella rawlinsi*, each species occurs in a markedly different habitat, despite their adjacent locations in the Sierra de Baoruco. As presented under Ecology for each species, *Brasiella iviei* is found in moist habitats of mixed deciduous forests, whereas *Brasiella rawlinsi* occurs in drier grassland habitats within pine forests.

### Brasiella ocoa sp. n.

urn:lsid:zoobank.org:act:444EED26-C691-4402-8A11-1C56D07E2446

http://species-id.net/wiki/Brasiella_ocoa

Fig. 11

#### Holotype.

Male! labeled “DOMINICAN REPUBLIC: / Peravia Prov. / Rio Ocoa-0.8km S, 0.2km W / Hy. 2-Las Carreras, 130 m / 18°20.7'N, 70°29.3'W / D. Brzoska 18-X-2005” [typeset black on white label]; Carnegie Museum / Specimen Number / CMNH-493,579” [typeset black on white label]; “HOLOTYPE / Brasiella / ocoa / Acciavatti” [typeset black on red label]. [Genitalia placed in glycerin within a microvial pinned under the holotype.]

#### Allotype.

Female unknown.

#### Paratypes.

One male! labeled with same label data as holotype; “PARATYPE / Brasiella / ocoa / Acciavatti” [typeset black on red label].

#### Type Depositories.

Holotype at CMNH; CMNH Unique Number stored in data files at CMNH. Paratype at DWBC.

#### Type Locality.

DOMINICAN REPUBLIC: Peravia Province, Las Carreras vicinity along Rio Ocoa, 0.8 km south and 0.2 km west of Highway 2, 18°20.7'N, 70°29.3'W, 130 m. Aerial view in Fig. 19D.

#### Diagnosis.

Distinguished from other *Brasiella* species on Hispaniola by the following combination of characters: 1) small body size of males; 2) head and pronotum shiny, shiny copper; 3) proepisterna shiny copper; 4) elytral markings bold and complete, pale tan to white against a duller cupreous ground color with metallic blue and green flecks scattered randomly over the unmarked portions of their surface; 5) male genitalia with a long aedeagus neck and a apical hook rounded nearly at a right angle and tip short; 6) aedeagus inner sac stylet short with recurved tip; 7) aedeagus inner sac large tooth short, narrowly pointed at tip; 8) aedeagus inner sac shield angled distad.

#### Description.

**Figure 11.**
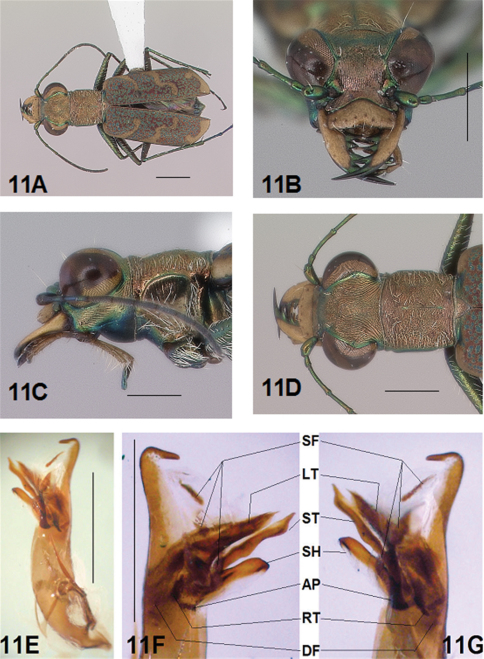
*Brasiella ocoa*, sp. n., male. Holotype **11A** Body, dorsal **11B** Head, anterior **11C** Body, anterior, left lateral **11D** Body, anterior, dorsal **11E** Aedeagus, dorsal **11F** Aedeagus inner sac, ventral aspect, and **11G** Aedeagus inner sac, dorsal aspect–AP, arched piece; LT, large tooth; SH, shield; ST, stylet; SF, spine fields (one f displaced from within aedeagus neck and two isolated); RT, root of LT; DF, dark fields. [Scale lines = 1 mm].

*General.* Fig. 11A. *Body.* Formelongate; head broad, eyes slightly bulging; pronotum broad and square; elytra slender. *Size*.Males, length 5.8-5.9 mm, width 1.8-1.9 mm; female unknown.

*Head*. Figs 11B, 11D. Shiny, shiny copper dorsally, copper green laterally, blue green ventrally; entire surface glabrous except for two pairs of supraorbital sensory setae. Frons finely and longitudinally rugose. Vertex more coarsely rugose, transverse rugae along anterior margin narrow and irregularly arranged, 16-18 more or less complete longitudinal rugae between eyes and middle where rugae converge into an arcuate pattern; rugae transition abruptly into a posterior area with a finely and irregularly granulate surface. Eyes prominent, slightly bulging laterally. Genae longitudinally rugose. Clypeus finely and irregularly granulate, anterior margin broadly arcuate. Labrum testaceous with a dark brown margin, subrectangular, width to length ratio 3 in holotype male; anterior margin sinuate, prominent at middle, a small bulge on either side of a tiny tooth; medial carina broadly and distinctly raised; 7 setae in an irregular row near middleasymmetrically arranged. Maxillae and labium mainly testaceous, only distal palpal segments dark brown with metallic blue green reflections. Mandibles in male with surface mainly testaceous, only teeth metallic green; mandibles symmetrical, four teeth distad of molar, apical tooth longest, first and second tooth coequal in length, both shorter than third tooth; gaps between three intermediate teeth narrow in male. Antennae 11 segmented; scape entirely to partially shiny green and slightly testaceous ventrally; scape with a single subapical sensory seta; antennomeres 2-4 shiny green to slightly testaceous, glabrous except for a few, short erect setae along their length and distally; antennomeres 5-11 dull brown, sheathed with dense short sensory setae.

*Prothorax.* Figs 11C, 11D. Pronotum shiny, shiny copper. Proepisterna shiny copper, surface wrinkled dorsad. Prosternum shiny green. Pronotum glabrous except for short, decumbent, white setae distributed in several, irregular rows medially directed, originating close to, and lying in a narrow band nearly impinging on lateral suture, in a sparse narrow band transversely and anteriorly oriented within broad anterior margin, and in a sparse narrow band laterally oriented on each side of midline extending nearly to the narrow posterior margin; transverse submarginal sulci distinct, anterior sulcus shallow, posterior sulcus deeper and deepest at posterior angles; transverse rugae within broad anterior margin and within posterior margin parallel, distinctly and deeply engraved, extending onto midline; surface sculptured by finer, transverse rugae angled on disc and interrupted by a finely engraved longitudinal midline, and more finely and irregularly sculptured elsewhere. Proepisterna glabrous except for white, erect and appressed setae arising from small setigerous punctures scattered over most of the surface in males.

*Pterothorax.* Fig. 11C. Mesepisterna glabrous except for appressed setae near ventral margin; female coupling sulcus represented by a small depression medially situated, a distinct groove extends only dorsally from pit, surface smooth below pit. Mesepimeron with sparse appressed setae. Metepisterna with scattered appressed setae, more abundant in male than female. Prosternum and mesosternum glabrous, smooth to slightly wrinkled; metasternum glabrous except for long, dense white appressed setae laterad, surface smooth mesad and coarsely sculpted laterad where setae originate. Scutellum triangular, cupreous.

*Legs.* Fig. 11A. Segmentsentirely shiny, metallic yellow green to partially testaceous brown with metallic green reflections. Coxae shiny, dull metallic brown green; trochanters shiny testaceous; femora and tibiae entirely metallic green to partially testaceous with metallic green reflections anteriorly; tarsomeres dark metallic brown; white, appressed setae on front and middle coxae, and laterally on hind coxae with one (rarely two) sensory setae mesad; erect setae and suberect closely spaced in several regular and irregular rows on all femora; setae widely spaced in a few rows on all tibiae; middle tibiae with patch of appressed setae dorsally along distal half; tarsomeres with short scattered setae on ventral surface; distal tarsomeres with two asymmetrical rows each with a few to several small, erect setae; an erect subapical seta present only on front trochanter, absent on middle and hind trochanters; males with dense pad of erect setae ventrally on proximal three tarsal segments; tarsal claws small.

*Elytra.* Fig. 11A. Elytra elongate, narrow, sides nearly parallel, only slightly broader at outer apical angle; evenly curved along posterior margins with apices separately rounded; sutural spine very small, prominent, feebly withdrawn from apex; posterior margins finely microserrulate. Surface finely granulate, impunctate, numerous small, irregular, shiny green or blue green flecks of various sizes scattered over a dull, dark copper brown background; fully developed elytral pattern of broad, bold markings contrasting with the darker elytral ground color; setigerous punctures with short, erect, transparent setae indistinct in subsutural rows on disc, but distinct at elytral base, and at inner humeral angles, each surrounded by a metallic fleck slightly larger than flecks elsewhere on elytra; surface very slightly depressed in humeral area and on disc creating only a very slight raised area basally. Elytral markings tawny, bold and distinct, forming a complete pattern consisting of humeral and apical lunules and middle band; humeral lunule complete terminating as a slightly enlarged end on disc in holotype, slightly broken before enlarged posterior end in paratype; middle band distinctly sinuate, complete, edges irregular, slightly enlarged near suture and slightly expanded along lateral margin; apical lunule complete broadened at suture. Elytral epipleura testaceous except for narrow, metallic green to copper green band along dorsal margin.

*Abdomen.* Not Fig.d. Male surface of 1st-5th sterna shiny black with green reflections, 6th sternum entirely shiny black green; posterior margins of male 3rd-5th sterna dark brown; male 3rd-5th sterna medially smooth with scattered, fine, erect setae in both sexes; male 1st-6th sterna laterally covered with dense, scattered, appressed white setae and roughened from setal punctures; male 6th sternum glabrous medially with a broad, deep concave notch.

*Male Genitalia.* Figs 11E, 11F, 11G. Shape narrow near base, uniformly broad in middle half, slim distally with neck long and narrow, apical hook evenly rounded nearly at a right angle, tip short. Aedeagus inner sac sclerites: stylet short, tip recurved; shield angled distad; large tooth short, narrow and pointed at tip with root and small dark fields; arched piece short and thick; spine field within aedeagus neck short and narrow.

#### Ecology.

Adults of this species are active in October based on the type series of two male specimens collected by David Brzoska on sand and gravel in the flood plain of the Rio Ocoa at 130 m (Fig. 19D). *Brasiella ocoa* occurred at its type locality in association with a much larger tiger beetle species, *Cicindela (Plectographa) schafferi* W. Horn 1903, based on information from Dave Brzoska that accompanied the *Brasiella ocoa* specimens. Robert L. Davidson also collected *Cicindela schaefferi* specimens at this Rio Ocoa location on a 1987 CMNH expedition to the Dominican Republic. He collected adults of this larger tiger beetle species both on the shoreline and on the gravel riverbed along the Rio Ocoa (Fig. 19D).

#### Distribution.

Fig. 22. DOMINICAN REPUBLIC: Peravia Province, Las Carreras vicinity along Rio Ocoa flood plain south of Highway 2. This species likely occurs elsewhere along the Rio Ocoa south toward the coast and further north into the Cordillera Central.

#### Etymology.

This Latinized eponym, a noun in aposition, is derived the Rio Ocoa, a major river that originates in the southern end of the Cordillera Central, flows across the southwestern coast of Peravia Province, and empties into the Caribbean Sea along the border with Azua Province in the Dominican Republic.

#### Remarks.

*Brasiella ocoa*, new species, superficially resembles *Brasiella dominicana*, an allopatric species that occurs nearby apparently along the Rio Bani (refer to Ecology for the latter species). However, their distinctiveness as separate species has been established by differences in the sclerites within the adult male aedeagus and other characters presented in the key and under their descriptions. Nonetheless, that fact that both species appear to have similar habitat requirements in low elevation flood plains, in contrast to the high elevation montane habitat requirements of most other *Brasiella* species known from Hispaniola, suggests that they may share a common lineage.

### Brasiella philipi sp. n.

urn:lsid:zoobank.org:act:762DC18A-4FF3-48F7-A81D-5A2B36695D8D

http://species-id.net/wiki/Brasiella_philipi

Figs 12 13

#### Holotype.

Male! labeled “DOMINICAN REPUBLIC: / Santiago Province / Cierrecita above / Mata Grande, 1290 m / Brian Farrell,coll.” [typeset black on white label]; “15 August 2008 / 19-13-05N, 070-59-52 W / North slope of the / Cordillera Central” [typeset black on white label]; “HOLOTYPE / Brasiella / philipi / Acciavatti” [typeset black on red label]. [Genitalia in glycerin in a microvial pinned beneath specimen.]

#### Allotype.

Female! labeled with same locality data as the holotype; allotype with “ALLOTYPE / Brasiella / philipi / Acciavatti” [typeset black on red label).

#### Paratypes.

Specimens! as follows: 1) 3 males and 3 females labeled with same locality data as the holotype, each with “PARATYPE / Brasiella / philipi / Acciavatti” [typeset black on blue label]; [these paratypes each labeled with a CMNH Unique Number]; 2) 1 male and 2 females labeled “fthills Cord.Cent. / S. of Santiago / June'38,Dom.Rep. / Darlington” [typeset black on white paper]; “C. (Brasiella) / dominicana Mandl / det. R. Freitag / April 1988” [typeset black on white label]; “PARATYPE / Brasiella / philipi / Acciavatti” [typeset black on blue label]; [these paratypes each labeled with a CMNH Unique Number on file].

#### Additional Specimens.

Male specimen! not a paratype labeled “DOMINICAN REPUBLIC / Los Tablones-Aqüita Fria, / Parque Nac. A. Bermúndez / La Vega Prov., 18.vii.2002, / DPerez, BHierro, RBastardo” [typeset black on white label]; “Specimen is property of / Museo Nacional de / Historia Natural / Santa Domingo, / Républica Dominicana” [typeset black on white label]; “Brasiella / philipi / Acciavatti” [typeset black on white label]. This male specimen agrees with the concept of this new species and appears conspecific with its holotype based on comparing its genitalia. However, because the specimen originated in a different province from the type locality, I have decided not to include it in the type series. This non-paratypic male is at MNHN.

#### Type Depositories.

Holotype, allotype, 4 paratypes (2 each sex) at MCZH; 5 paratypes (3 males, 2 females) at CMNH. [Male paratypes at CMNH with Carnegie Museum Specimen Numbers: CMNH-536,894; 539,785; 542,951. Female paratypes CMNH with Carnegie Museum Specimen Numbers: CMNH-488,249; 497,224.] One non-paratypic male at MNHN.

#### Type Locality.

DOMINICAN REPUBLIC: Santiago Province, Cierrecita above Mata Grande, 19°13'05"N, 70°59'52"W, north slope of the Cordillera Central, 1290 m. Cierrecita is a small community on a ridge to the north of Mata Grande which itself lies on the north side of the Rio Bao. Aerial view in Fig. 19E.

#### Diagnosis.

Distinguished from other *Brasiella* species on Hispaniola by the following combination of characters: 1) elytral lunules tawny, fully developed, wide and contrasting with darker, dull copper brown background; 2) middle band of nearly uniform width and distinctly recurved anteriorly near suture; 3) male genitalia bulky, aedeagus distal neck short and broadly, apical hook evenly rounded, tip elongated and nearly at right angle to aedeagus; 4) aedeagus inner sac stylet tip recurved, tapering to sharp point; 5) aedeagus inner sac shield tapered distad; 6) aedeagus inner sac apical spine field in neck short and wide, forming a distinct pad; 7) female 5^th^ abdominal sternum with transverse wrinkles, a narrow membranous band along the midline, and a small membranous wedge along posterior margin; 8) female 6^th^ abdominal sternum with large lateral gibbosities.

#### Description.

**Figure 12.**
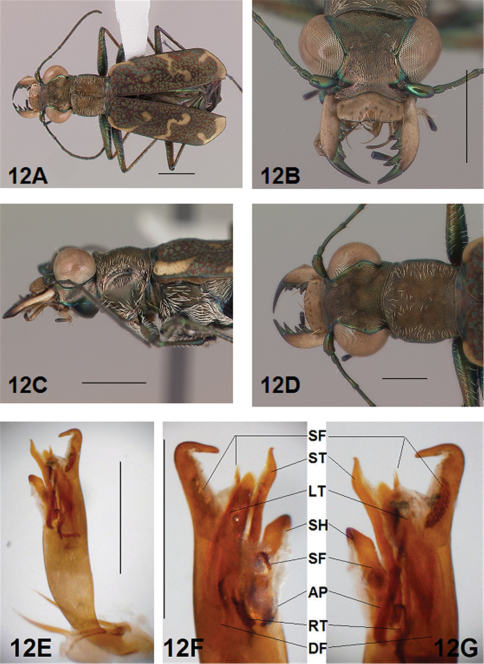
*Brasiella philipi*, sp. n., male. Holotype **12A** Body, dorsal **12B** Head, anterior **12C** Body, anterior, left lateral **12D** Body, anterior, dorsal **12E** Aedeagus, dorsal **12F** Aedeagus inner sac, ventral aspect, and **12G** Aedeagus inner sac, dorsal aspect–AP, arched piece; LT, large tooth; SH, shield; ST, stylet; SF, spine fields (one within aedeagus neck and two isolated); RT, root of LT; DF, dark fields. [Scale lines = 1 mm].

**Figure 13.**
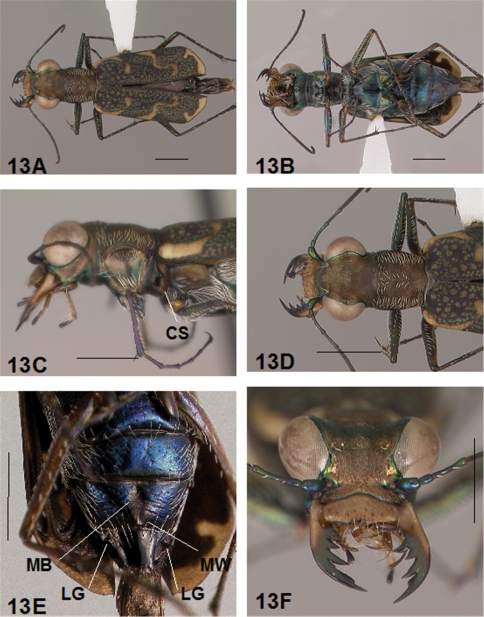
*Brasiella philipi*, sp. n., female. Allotype **13A** Body, dorsal **13B** Body, ventral **13C** Body, anterior, left lateral–CS, coupling sulcus **13D** Body, anterior, dorsal **13E** Abdomen, sterna, ventral–5^th^ sternum, MB, membranous band; MW, membranous wedge; 6^th^ sternum, LG, lateral gibbosity **6F** Head, anterior. [Scale lines = 1 mm].

*General.* Figs 12A, 13A. *Body.* Formelongate; head narrow, eyes prominent, slightly bulging laterally; pronotum subarcuate, wider than long; elytra broadened distad, each slightly rounded. *Size*.Males, length 6.0-6.4 mm, width 1.9-2.0 mm; females, length 6.4-6.7 mm, width 2.0-2.1 mm.

*Head*. Figs 12B, 12D, 13D, 13F. Shiny dark copper brown to dark brown dorsally and blue green ventrally; entire surface glabrous except for two pairs of supraorbital sensory setae. Frons finely and longitudinally rugose. Vertex more coarsely rugose, transverse rugae along anterior margin narrow and irregularly arranged, 15-18 more or less complete longitudinal rugae between eyes and middle where rugae converge into an arcuate pattern; rugae transition abruptly into a posterior area with a finely and irregularly granulate surface. Eyes prominent, slightly bulging laterally. Genae longitudinally rugose. Clypeus finely and irregularly granulate, narrowed mesad. Labrum testaceous with a dark brown margin, subrectangular, width to length ratio 2.2 in holotype male, ratio 2.6 in allotype female; anterior margin sinuate, a small bulge on either side of a tiny medial tooth in most specimens, tooth absent in others specimens; posterior margin broadly arcuate mesad; medial carina narrow, distinctly raised with a broad depression on either side; 6-8 setae in an irregular row near middle, most often symmetrically arranged. Maxillae and labium mainly testaceous, only distal palpal segments dark brown with metallic blue green reflections. Mandibles sexually dimorphic; in male, surface mainly testaceous, only teeth metallic green; in female, surface only testaceous in basal half, apical half and teeth shiny brown; mandibles symmetrical, four teeth distad of molar, apical tooth longest, third and first tooth coequal in length, second tooth shortest; gaps between three intermediate teeth narrow in male, wide in female. Antennae 11 segmented; scape dorsally shiny green with a single subapical sensory seta; antennomeres 2-4 shiny green, glabrous except for a few, short erect setae along their length and distally; antennomeres 5-11 black green, sheathed with dense short sensory setae.

*Prothorax.* Figs 12C, 12D, 13C, 13D. Pronotum shiny dark copper brown. Proepisterna shiny copper black, surface wrinkled dorsad. Prosternum shiny green. Pronotum glabrous except for short, decumbent, white setae distributed in several, irregular rows medially directed, originating close to, and lying in a narrow band distinctly removed from lateral margin, in a sparse narrow band transversely and anteriorly oriented within broad anterior margin, and in a sparse narrow band laterally oriented on each side of midline extending nearly to the narrow posterior margin; transverse submarginal sulci distinct, anterior sulcus shallow, posterior sulcus deeper and deepest at posterior angles; transverse rugae within broad anterior margin irregular and shallow, interrupted at middle by an irregularly arranged pattern, within posterior margin more distinctly and deeply engraved especially medially and extending onto midline; surface sculptured by fine, transverse rugae angled on disc and interrupted by a finely engraved longitudinal midline, and more finely and irregularly sculptured elsewhere. Proepisterna glabrous except for white, erect and appressed setae arising from small, setigerous punctures scattered over ventral half and along posterior margin in males, only near ventral margin females. Prosternum glabrous.

*Pterothorax.* Figs 12C, 13C. Mesepisterna glabrous except for appressed setae near ventral margin; female coupling sulcus represented by a small depression medially situated, a distinct groove extends only dorsally from pit, surface smooth below pit. Mesepimeron with sparse appressed setae. Metepisterna with scattered appressed setae, more abundant in male than female. Prosternum and mesosternum glabrous, smooth to slightly wrinkled; metasternum glabrous except for long, dense white appressed setae laterad, surface smooth mesad and coarsely sculpted laterad where setae originate. Scutellum triangular, copper to dark cupreous black.

*Legs.* Figs 12A, 13B. Segmentsmetallic green with copper reflections or metallic green and testaceous. Coxae shiny metallic brown green; trochanters shiny testaceous; femora and tibiae testaceous with metallic green reflections anteriorly along most of their lengths except distal ends; tarsomeres dark metallic violet black; white, appressed setae on front and middle coxae, and laterally on hind coxae; erect setae and suberect closely spaced in several regular and irregular rows on all femora; setae widely spaced in a few rows on all tibiae; middle tibiae with patch of appressed setae dorsally along distal half; tarsomeres with short scattered setae on ventral surface; distal tarsomeres with two asymmetrical rows each with a few to several small, erect setae; an erect subapical seta present only on front trochanter, absent on middle and hind trochanters; males with dense pad of erect setae ventrally on proximal three tarsal segments; tarsal claws small.

*Elytra.* Figs 12A, 13A. Form narrow in male, broadened slightly distad and broadest at outer apical angle in female; evenly curved along posterior margins with apices separately rounded; sutural spine small, feebly withdrawn from apex; posterior margins finely microserrulate. Surface finely granulate, impunctate, numerous small, irregular metallic blotches comprised of shiny green or blue green flecks of various sizes scattered over a dull, dark copper brown background; fully developed elytral pattern of bold markings contrasting with the darker elytral ground color; setigerous punctures with short, erect, transparent setae indistinct in subsutural rows on disc, but distinct at elytral base, and at inner humeral angles, each surrounded by a metallic fleck slightly larger than flecks elsewhere on elytra; surface slightly depressed in humeral area and on disc creating a slight but distinct raised area basally. Elytral markings tawny, fully developed, wide and contrasting with darker, dull copper brown background; pattern consisting of humeral and apical lunules and middle band; humeral lunule complete terminating as a slightly enlarged end on disc in most specimens, slightly broken at posterior end in a few specimens; middle band of nearly uniform width, distinctly recurved anteriorly near suture, and slightly expanded along lateral margin; apical lunule wide, complete and broadened near suture in all specimens examined. Elytral epipleura testaceous except for narrow, metallic green to copper green band along dorsal margin.

*Abdomen.* Figs 13B, 13E. Surface of 1st-5th sterna shiny black with green reflections, 6th sternum entirely shiny black to black brown; posterior margins of male 3rd-5th sterna and female 3rd-4th sterna narrowly black; posterior margin female 5th sternum broadly black; 3rd-5th sterna medially smooth with scattered, fine, erect setae in both sexes; male 1st-6th sterna and female 1st-5th sterna laterally covered with dense, scattered, appressed white setae and roughened from setal punctures; male 6th sternum glabrous medially with a broad, deep concave notch; female 5th sternum with moderately raised, transverse wrinkles and a membranous band at midline extending anteriorly along most of the sternum from a small, transverse membranous wedge along posterior margin; female 6th sternum entirely glabrous, posterior margin with a row of 6-10 erect spines and a large lateral gibbosity on each side.

*Male Genitalia.* Figs 12E, 12F, 12G. Shape bulky, narrow only basally, uniformly broad along most of its length, distal neck short and broad, apical hook evenly rounded, tip elongated and nearly at right angle to aedeagus. Aedeagus inner sac sclerites: stylet tip recurved, tapering to sharp point; shield tapered distad; large tooth long and pointed at tip with large root and large dark fields; arched piece long and thin; spine field within aedeagus neck short and and wide, forming a distinct pad.

#### Ecology.

This species occurs along dirt roads, road cuts, and bare areas in fields in the foothills on the north slope of the Cordillera Central at 1290 m elevation (Figs 19E, 19F). From the collection records June, July, and August, it is concluded that adults of this new species are active during most summer months.

#### Distribution.

Fig. 22. DOMINICAN REPUBLIC, Santiago Province, and La Vega Province, on the north slopes of the Cordillera Central. This species likely will be found in suitable habitats distributed over the entire northern slopes of the Cordillera Central.

#### Etymology.

This Latinized eponym, genitive case, is based on the first name of the late Philip J. Darlington, Museum of Comparative Zoology, Harvard University, Cambridge, Massachusetts. Darlington was the distinguished American carabidologist who pioneered studies of Carabidae island biogeography in the Mid-20th Century. The first specimens of this new species were collected by Darlington during his 1938 expedition to the Dominican Republic. Brian Farrell, also from MCZH, in 2008 collected additional specimens with more precise locality data, while retracing the route of Darlington's expedition 50 years earlier.

#### Remarks.

Both *Brasiella philipi*, new species, and *Brasiella bellorum*, new species, are allopatric in the Cordillera Central, Dominican Republic, the former species on the northern slopes and the latter species in the mountainous central areas. The elytral pattern of both species is very similar and only differs in the form of the elytral lunules. *Brasiella philipi* possesses more fully developed and wider lunules of nearly uniform width, whereas the lunules of *Brasiella bellorum* are narrow and broken. These lunule differences are most obvious in the shape of the hook at the discal end of the middle band. Although the elytral patterns are similar, the distinctiveness of each species is established by differences in their genitalia, especially the form of the sclerites and spine fields within the aedeagus of the males. A comparison of the other morphological characters presented in the key and the descriptions for each species further support their distinctiveness. Although these two species are apparently allopatric based on available collection data, more collecting in the Cordillera Central will help confirm their allopatry. Despite their distinctiveness as separate species and apparent allopatry, it should be noted that these two species are found in similar mountain habitats with adults of both species active during the same summer months. Their geographic distributions in close proximity to each other in similar high elevation habitats suggest a common lineage.

### Brasiella rawlinsi sp. n.

urn:lsid:zoobank.org:act:E744F348-18DE-434F-9073-B84C2B35C1F0

http://species-id.net/wiki/Brasiella_rawlinsi

Figs 14 15

#### Holotype.

Male! labeled “DOMINICAN REPUBLIC: / Pedernales. 37 km N / Cabo Rojo, 1500 m. / 18-09N, 71-35W” [typeset black on white label]; “25 September 1991. / J.Rawlins,R.Davidson /C.Young, S. Thompson / Grassland with pines” [typeset black on white label]; “Carnegie Museum / Specimen Number / CMNH-125,928” [typeset black on white label]; “HOLOTYPE / Brasiella / rawlinsi / Acciavatti” [typeset black on red label]. [Genitalia stored in glycerin in a microvial pinned beneath specimen.]

#### Allotype.

Female! labeled with the same locality data as the holotype; “Carnegie Museum / Specimen Number / CMNH-136,271” [typeset black on white label]; “ALLOTYPE / Brasiella / rawlinsi / Acciavatti” [typeset black on red label].

#### Paratypes.

Specimens! as follows: 1) 80 males and 45 females labeled with the same locality data as the holotype; “PARATYPE / Brasiella / rawlinsi / Acciavatti” [typeset black on blue label]; these paratypes each labeled with a CMNH Unique Number; 2) 20 males and 5 females labeled “DOMIN.REP.: Pedernales / Prov., El Accitillar” (sic) / “ca.35km NNW.Cabo Rojo / 23AUG1988,1370-1430m” [typeset black on white label]; “M.A.Ivie,T.K.Philips / & K.A.Johnson colrs.” [typeset black on white label]; “PARATYPE / Brasiella / rawlinsi / Acciavatti” [typeset black on blue label]; 3) 4 males and 6 females labeled “DOMIN.REP.: Pedernales / Prov., El Accitillar” (sic) / “ca.35km NNW.Cabo Rojo / 26AUG1988, 1325-1370m” [typeset black on white label]; “M.A.Ivie,T.K.Philips / & K.A.Johnson colrs.” [typeset black on white label]; “PARATYPE / Brasiella / rawlinsi / Acciavatti” [typeset black on blue label]; 4) 10 males and 6 females labeled “DOMIN.REP.: Pedernales / Prov., El Accitillar” (sic) / “ca.35km NNW.Cabo Rojo / 09SEP1988, 1430m” [typeset black on white label]; “M.A.Ivie,T.K.Philips / & K.A.Johnson colrs.” [typeset black on white label]; “PARATYPE / Brasiella / rawlinsi / Acciavatti” [typeset black on blue label]; 5) 1 male labeled “DOM.REP: Pedernales / ca.35km NNW.Cabo Rojo / 1370 m, El Aceitillar / 26AUG-09 SEP1988 / flight intercept trap” [typeset black on white label]; “M.A.Ivie,T.K.Philips / & K.A.Johnson colrs.” [typeset black on white label]; “PARATYPE / Brasiella / rawlinsi / Acciavatti” [typeset black on blue label]; 6) 6 males and 7 females labeled “DOMIN.REP.:Prov.Pedern. / Las Abejas-El Accitillar” (sic) / “ca.35km NNW.Cabo Rojo / 23AUG1988,1250-1430m” [typeset black on white label]; “M.A.Ivie,T.K.Philips / & K.A.Johnson colrs.” [typeset black on white label]; “PARATYPE / Brasiella / rawlinsi / Acciavatti” [typeset black on blue label]; 7) 1 male and 1 female labeled “DOM.REP.:Prov.Pedernales / ca.35km N Cabo Rojo / El Aceitillar-Las Abejas / 1250-1430m, 23 AUG 1988 / M.Ivie,Philips & Johnson” [typeset black on white label]; “PARATYPE / Brasiella / rawlinsi / Acciavatti” [typeset black on blue label]; 8) 4 males and1 female from MNHN, each labeled as previous specimens, along with several other labels, a unique specimen number from 30,097 to 30,101 [typed on white label]; a unique bar code with a number; “Photographed / 2002-2003 / CBSD” [typeset black on white label], “Specimen property of / MUSEO NACIONAL de / HISTORIA NATURAL / Santa Domingo, / REPUBLICA DOMINICANA” [typeset black on white label], “PARATYPE / Brasiella / rawlinsi / Acciavatti” [typeset black on blue label]. One male of the MNHN paratypes also labeled “Cicindela / dominicana / Mandl / det. M.A. Ivie 1989” [handprinted and typeset black on white label, black border]; “Brasiella / prob. / n.sp.” [handprinted black on white label, black border].

#### Type Depositories.

Holotype, allotype, 133 paratypes at CMNH [each CMNH Unique Number stored in data files at CMNH]; 61 paratypes (39 males, 22 females) at WIBP; 6 paratypes (3 each sex) at WKSU; 5 paratypes (4 males, 1 female) at MNHN [each MNHN Unique Number and Bar Code stored in data files at MNHN].

#### Type Locality.

DOMINICAN REPUBLIC: Pedernales Province, 37 km N Cabo Rojo, 18°09'N, 71°35'W, 1500 m. Aerial view in Fig. 21A.

#### Notes on Type Locality.

It is likely that the actual type locality lies at a slightly higher elevation than provided in this revision. CMNH entomologists visited this type locality again in June 2003, two years after the type series was collected (J.E. Rawlins, R.L. Davidson, 2010, personal communications). At that time, perhaps with improved GPS devices, the following coordinates and elevation were obtained with more precision: 18°09'23"N, 71°34'09"W at a slightly higher elevation of 1560 m. These coordinates and the elevation are based on data published by Woodruff (2004) for Scarabaeidae specimens collected at Aceitillar by Robert L. Davidson and John E. Rawlins on the 1987, 1991 and 2003 CMNH expeditions to this locality in the Sierra de Baoruco, Dominican Republic (Figs 20E, 20F). According to Woodruff (2004), the locality known as Aceitillar is based on a common *Andropogon* sp.grass that grows there in assocation with *Pinus occidentalis* Swartz; this grass is known locally as aceitillo; aceitillar being the place where the aceitillo grows. Indeed, Accitillar [Aceitillar] was printed on labels for certain paratypes of *Brasiella rawlinsi* originating with WIBP, and later Aceitillar appeared on labels from the June 2003 CMNH expedition to the Sierra de Baoruco.

#### Diagnosis.

Distinguished from other *Brasiella* species on Hispaniola by the following combination of characters: 1) head and pronotum of similar length in both sexes; 2) eyes small, not prominent nor bulging laterally; 3) head and pronotum shiny copper red, elytra dull cupreous or coppery green; 4) elytra unmarked in most specimens or only faintly marked in others or marked more distinctly in a few; 5) cupreous or coppery green elytral ground color and faint elytral markings infuscated with metallic blue and green flecks; 6) male genitalia apical neck short and wide, apex inner and outer angles evenly rounded, tip acutely bent and terminating in a short point; 7) aedeagus inner sac stylet thin and straight to slightly bent distad.

#### Description.

**Figure 14.**
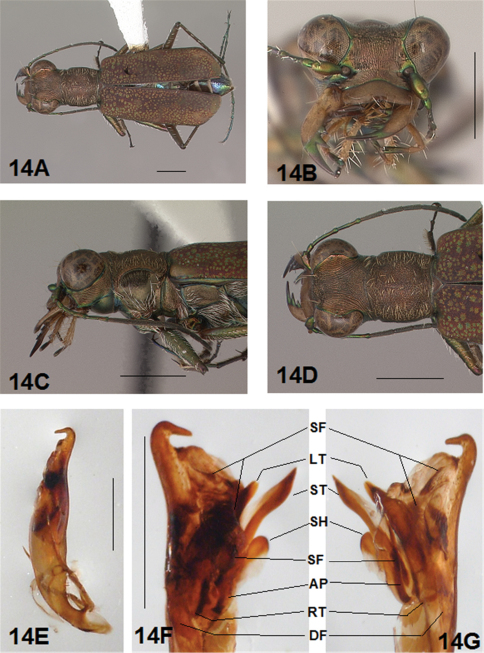
*Brasiella rawlinsi*, sp. n., male. Holotype **14A** Body, dorsal **14B** Head, anterior **14C** Body, anterior, left lateral **14D** Body, anterior, dorsal **14E** Aedeagus, dorsal **14F** Aedeagus inner sac, ventral aspect, and **14G** Aedeagus inner sac, dorsal aspect–AP, arched piece; LT, large tooth; SH, shield; ST, stylet; SF, spine fields (one displaced from within aedeagus neck and two isolated); RT, root of LT; DF, dark fields. [Scale lines = 1 mm].

**Figure 15.**
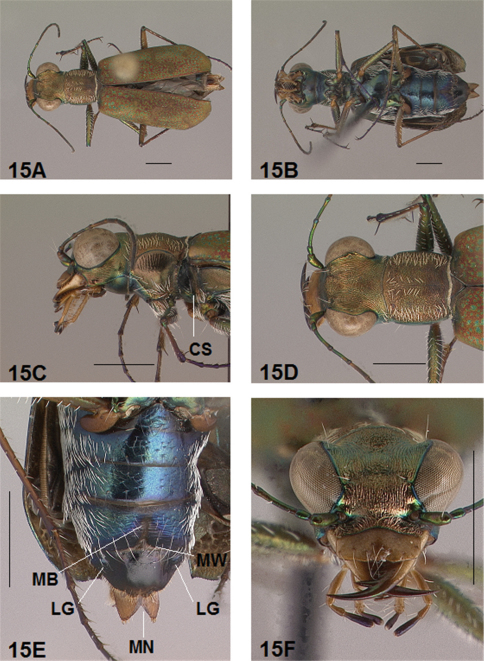
*Brasiella rawlinsi*, sp. n., female. Allotype **15A** Body, dorsal **15B** Body, ventral **15C** Body, anterior, left lateral–CS, coupling sulcus **15D** Body, anterior, dorsal **15E** Abdomen, sterna, ventral–5^th^ sternum, MB, membranous band; 5^th^ sternum, MW, membranous wedge; 6^th^ sternum, LG, lateral gibbosity; 8^th^ sternum, MN, median notch **15F** Head, anterior. [Scale lines = 1 mm].

*General.* Figs 14A, 15A. *Body.* Formslender; head large, eyes proportionally small, not prominent nor bulging laterally; pronotum square, width equals length; elytra narrow, slightly broadened distad, posterior margins evenly rounded, apices separately rounded. *Size*.Males, length 5.8-6.5 mm, width 1.8-2.1 mm; females, length 6.5-7.0 mm, width 2.0-2.2 mm.

*Head*. Figs 14B, 14D, 15D, 15F. Shiny copper brown dorsally with green reflections, and black green ventrally; entire surface glabrous except for two pairs of supraorbital sensory setae. Frons finely and longitudinally rugose. Vertex more coarsely rugose, transverse rugae along anterior margin narrow and irregularly arranged, 15-18 more or less complete longitudinal rugae between eyes and middle where rugae converge, forming an arcuate to slightly circular pattern; rugae transition abruptly into a posterior area with a finely and irregularly granulate surface. Eyes small, neither prominent nor bulging laterally. Genae longitudinally rugose. Clypeus finely and irregularly granulate, narrowed mesad. Labrum testaceous with a dark brown margin, rectangular in male with width to length ratio 4 in holotype male, subrectangular in female with width to length ratio 2 in allotype female; anterior margin transverse in male with a minute tooth mesad, very slightly protruding at middle in female with a small tooth mesad; posterior margin broadly arcuate mesad; medial carina very broadly, slightly raised, without an obvious depression on either side; 4-8 setae in an irregular row near middle most often symmetrically arranged, fewer setae in male. Maxillae and labium mainly testaceous, only distal palpal segments dark brown with metallic blue green reflections. Mandibles sexually dimorphic; in male, surface mainly testaceous, only teeth shiny green; in female, surface only testaceous in basal half, apical half and teeth shiny to violet green; mandibles symmetrical, four teeth distad of molar, apical tooth longest, first and third tooth coequal in length, second tooth shortest; first and second teeth without a gap between them in male, gaps between three intermediate teeth wide in female. Antennae 11 segmented; scape dorsally shiny green, ventrally testaceous with a single subapical sensory seta; antennomeres 2-4 shiny green, glabrous except for a few, short erect setae along their length and distally; antennomeres 5-11 dull brown, sheathed with dense short sensory setae.

*Prothorax.* Figs 14C, 14D, 15C, 15D. Pronotum metallic copper brown with green reflections. Proepisterna shiny copper, surface wrinkled dorsad. Prosternum shiny green. Pronotum glabrous except for short, decumbent, white setae distributed in several, irregular rows inside lateral margin medially directed, originating close to and lying in a narrow band nearly impinging on lateral suture, in a sparse narrow band transversely and anteriorly oriented distinctly removed from anterior margin, and in a sparse narrow band laterally oriented on each side of midline extending nearly to the narrow posterior margin; transverse submarginal sulci distinct, anterior sulcus shallow, posterior sulcus deeper and deepest at posterior angles; transverse rugae within broad anterior margin irregular and shallow, interrupted at middle by an irregularly arranged pattern, within posterior margin more distinctly and deeply engraved especially medially and extending onto midline; surface sculptured by fine, transverse rugae angled on disc and interrupted by a finely engraved longitudinal midline, and more finely and irregularly sculptured elsewhere. Proepisterna glabrous except for white, erect and appressed setae arising from small setigerous punctures scattered over the surface near ventral margin in female, over ventral half of the surface and near the posterior margin in males. Prosternum glabrous.

*Pterothorax.* Figs 14C, 15C. Mesepisterna glabrous except for a few appressed setae near ventral margin; female coupling sulcus represented by a small depression medially situated, a distinct groove extends only dorsally from pit, surface smooth below pit. Mesepimeron with a few sparse appressed setae. Metepisterna with scattered appressed setae, more abundant in male than female. Prosternum and mesosternum glabrous, smooth to slightly wrinkled; metasternum glabrous except for long, dense white appressed setae laterad, surface smooth mesad and coarsely sculpted laterad where setae originate. Scutellum triangular, shiny cupreous.

*Legs.* Figs 14A, 15B. Segmentstestaceous brown with metallic brown green reflections. Coxae shiny metallic brown green; trochanters shiny testaceous; femora and tibiae testaceous with metallic green reflections anteriorly; tarsomeres dark metallic brown; white, appressed setae on front and middle coxae, and laterally on hind coxae; erect setae and suberect closely spaced in several regular and irregular rows on all femora; setae widely spaced in a few rows on all tibiae; middle tibiae with patch of appressed setae dorsally along distal half; tarsomeres with short scattered setae on ventral surface; distal tarsomeres with two asymmetrical rows each with a few to several small, erect setae; an erect subapical seta present only on front trochanter, absent on middle and hind trochanters; males with dense pad of erect setae ventrally on proximal three tarsal segments; tarsal claws small.

*Elytra.* Figs 14A, 15A. Form narrow in male, broadened slightly distad and broadest at outer apical angle in female; posterior margins evenly rounded, apices separately rounded; sutural spine tiny in male, small in female, feebly withdrawn from apex; posterior margins finely microserrulate. Surface finely granulate, impunctate, numerous small, irregular, metallic flecks of various sizes comprised of blue or blue green flecks scattered over a cupreous or coppery green background; setigerous punctures with short, erect, transparent setae indistinct in subsutural rows on disc, but distinct at elytral base, and at inner humeral angles, each surrounded by a metallic fleck slightly larger than flecks elsewhere on elytra; surface slightly depressed in humeral area and on disc creating a slight but distinct raised area basally. Elytra unmarked in most specimens, only faintly marked in others, or marked with more distinct tawny lunules in a few; elytral ground color cupreous in most specimens, coppery green in others or green in others; faint elytral markings infuscated with metallic blue green flecks; specimens with the most extensive infuscated markings appear immaculate, while others have faint and indistinct markings; faint markings comprised of a humeral lunule reduced to its extremities, a middle lunule terminating near the suture in a broad hook never anteriorly recurved, and an apical lunule usually reaching the suture. Elytral epipleura testaceous except for narrow, metallic green to copper green band along dorsal margin.

*Abdomen.* Figs 14B, 15E. Surface of 1st-5th sterna shiny black with green reflections, 6th sternum entirely shiny black to black brown; posterior margins of male 3rd-5th sterna and female 3rd-4th sterna narrowly black; posterior margin female 5th sternum broadly black; 3rd-5th sterna medially smooth with scattered, fine, erect setae in both sexes; male 1st-6th sterna and female 1st-5th sterna laterally covered with dense, scattered, appressed white setae and roughened from setal punctures; male 6th sternum glabrous medially with a broad, deep concave notch; female 5th sternum with moderately raised, transverse wrinkles interrupted by a short, wide membranous band along midline extending anteriorly to middle of sternum from a wide transverse membranous wedge along posterior margin; female 6th sternum entirely glabrous, posterior margin with a row of 6-10 erect spines and a large lateral gibbosity on each side.

*Male Genitalia.* Figs 14E, 14F, 14G. Shape narrow near base, widening gradually and uniformly broad in middle three-quarters, narrowing gradually distally with neck short and wide, apical hook inner and outer angles evenly rounded, tip short and acutely angled to aedeagus. Aedeagus inner sac sclerites: stylet thin and straight to slightly bent distad; shield rounded distad; large tooth wide and pointed at tip with large root and large dark fields; arched piece short and narrow; spine field within aedeagus neck short and thin.

#### Ecology.

This new species is found in the Dominican Republic across a range of higher elevations in the Sierra de Baoruco from 1250 to 1560 m. Its habitats are dominated by Hispaniolan pine, *Pinus occidentalis* Swartz, and *Andropogon* sp. grasses (Figs 20E, 20F, 21A, 21B). The red clay soil types prevalent in these habitats are derived from bauxite deposits that are surface mined and reclaimed extensively (Fig. 21A) in this part of the Sierra de Baoruco (Woodruff 2004). Adults have been collected in disturbed sites along dirt roads and trails during August and September. These adults were abundant along an infrequently traveled road where Robert L. Davidson collected the type series during the 1991 CMNH expedition to the Dominican Republic. This type locality is in the vicinity of the campsite used by the several CMNH expeditions to this locality (Fig. 20E). Adults were also abundant at several other sites at slightly lower elevations in 1988 based on WIBP collection data. It should be noted that CMNH entomologists have visited the type locality at other times of the year, July 1987 and June 2003, but no specimens of *Brasiella rawlinsi* were seen (R.L. Davidson, 2010, personal communication). Thus, adult activity for this new species appears restricted to a distinctive period during the late summer. In addition to hand collecting adults both running and flying, this new species has been taken in malaise and intercept traps. *Brasiella rawlinsi* occurs sympatrically with *Brasiella iviei* and *Brasiella youngi* in the Sierra de Baoruco, but in different habitats; the former species occurs in drier pine and grass woodlands, whereas the latter two species occur in mixed deciduous forests on the southern slopes of these mountains. Refer to the discussions under Ecology for *Brasiella iviei* and *Brasiella youngi* to obtain more details about their habitats.

#### Distribution.

Fig. 22. DOMINICAN REPUBLIC: Pedernales Province, Sierra de Baoruco at higher elevations in grass and pine habitats typical of the locality known as Aceitillar. Localities include: 37 km N Cabo Rojo at 1500 m (more precisely 1560 m); El Aceitillar ca. 35km NNW Cabo Rojo at elevations from 1250 m to 1430 m. This species in likely distributed throughout the Sierra de Baoruco at higher elevations in areas of red, bauxite soils in habitats with pines and grasslands.

#### Etymology.

This Latinized eponym, genitive case, is based on the family name of John E. Rawlins, Curator of Invertebrate Zoology, Carnegie Museum of Natural History, Pittsburgh, Pennsylvania. The species name honors John for his encouragement and the assistance he provided to move this revision toward its completion. He also has been a colleague and friend for several decades while the author has been associated with the CMNH. Dr. Rawlins organized the several expeditions to inventory the insect fauna of Hispaniola that resulted in the collections of several of the new *Brasiella* species described in this revision.

#### Remarks.

The distinctiveness of *Brasiella rawlinsi* from the closely related *Brasiella iviei* was established in this revision by differences presented in the key to their identification and under their descriptions. Other than the more obvious differences in smaller body size and proportionately shorter head and pronotum of *Brasiella rawlinsi* compared with *Brasiella iviei*, important distinctions exist between the male aedeagus of these two species. Both species differ in the shape of the apical hook, as well as, the form of the sclerites of the inner sac, especially the thickness and degree of curvature in the stylet apex. Additional differences between females of these two species are evident in the extent to which the membranous, longitudinal median band is developed on the 5^th^ abdominal sternum. Previously, M. Ivie (WIBP) and T.K. Philips (WKSU), who collected specimens of both these new species in the Sierra de Baoruco in 1988, initially thought the populations in these mountains might represent a new species; however, their unpublished study considered them only *Brasiella dominicana* variants (Philips 1994, personal communication).

### Brasiella youngi sp. n.

urn:lsid:zoobank.org:act:C4961C52-2126-47C3-89FC-22C54EAB6F34

http://species-id.net/wiki/Brasiella_youngi

Figs 16 17

#### Holotype.

Male! labeled “DOMIN.REP.:Prov.Pedernales / 13.5km N Cabo Rojo, 140m / 21AUG-10SEP1988, flight / intercept trap, M.A.Ivie / T.K.Philips & K.A.Johnson” [typeset black on white label]; “HOLOTYPE / Brasiella / youngi / Acciavatti” [typeset black on red label]. [Genitalia in glycerin in a microvial pinned beneath specimen.]

#### Allotype.

Female! labeled “DOMINICAN REPUBLIC: / Pedernales. 26 km N / Cabo Rojo, 730 m. / 18-06N, 71-38W” [typeset black on white label]; “25-27 September 1991. / R.Davidson, C.Young / S. Thompson, J.Rawlins / Wet deciduous forest” [typeset black on white label]; “Carnegie Museum / Specimen Number / CMNH-132,618” [typeset black on white label]; “ALLOTYPE / Brasiella / youngi / Acciavatti” [typeset black on red label].

#### Type Depositories.

Holotype at WIBP; allotype at CMNH, [CMNH Unique Number stored in data files at CMNH].

#### Type Locality.

DOMINICAN REPUBLIC: Pedernales Province, 13.5 km N Cabo Rojo, 18°02'04"N, 71°38'38"W at 140 m, in the Sierra de Baoruco. Aerial view taken at lower elevations on the southern slopes of the Sierra de Baoruco in Fig. 21E.

*Notes on the Type Locality*. No coordinates are on the holotype labels; however, an approximation of the coordinates shown above was obtained by measuring 13.5 km to 140 m along the only road north of Cabo Rojo using Google Earth©. Deciduous woodlands adjoining desert scrub typically are found at this elevation on the lower southern slopes of the Sierra de Baoruco (Figs 21E, 21F). The allotype was collected at a site called Haitian Hut on the CMNH 1991 expedition (J.E. Rawlins, 2010, personal communication). The site was given this name because of the Haitian squatters who have gained easy access to the lower southern slopes of the Sierra de Baoruco by using the highway built by Alcoa, Inc., to reach bauxite mines higher in the Sierra de Baoruco (Woodruff 2004). The Haitian Hut site (Figs 21C, 21D), located in the deciduous forest accessible from the Alcoa highway, has coordinates of 18°06'42"N, 71°37'21"W at 730 m at 26 km N of Cabo Rojo; these slightly more precise coordinates, than originally obtained in the field, were based on using Google Earth©.

#### Diagnosis.

Distinguished from *Brasiella* species on Hispaniola by the following combination of characters: 1) head small, eyes proportionally large, prominent and distinctly bulging laterally; 2) subglobose to subarcuate pronotum; 3) female legs and antennal scape primarily testaceous, male appendages dominated by dark metallic reflections; 4) female mesepisternal coupling sulcus a shallow, concentric depression with a small, central pit; 5) faint, nearly translucent elytral markings, forming a complete, but reduced pattern with humeral lunule divided into a humeral spot and a discal dot; 6) female 5th abdominal sternum with a small, membranous wedge along the posterior margin and a wide, membranous band extending along midline only to middle; 7) female 8^th^ sternum median notch shallowly incised.

#### Description.

**Figure 16.**
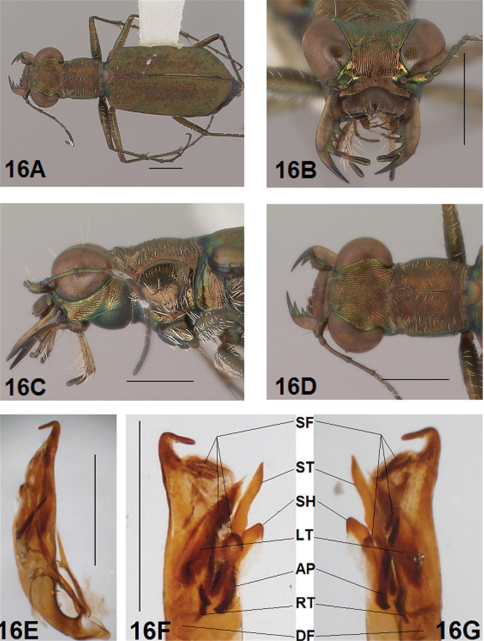
*Brasiella youngi*, sp. n., male. Holotype **16A** Body, dorsal **16B** Body, ventral **16C** Body, anterior, left lateral **16D** Body, anterior, dorsal **16E** Aedeagus, dorsal **16F** Aedeagus inner sac, ventral aspect, and **16G** Aedeagus inner sac, dorsal aspect–AP, arched piece LT large tooth; SH, shield; ST, stylet; SF, spine fields (one displaced from within aedeagus neck and two isolated); RT, root of LT; DF, dark fields. [Scale lines = 1 mm].

**Figure 17.**
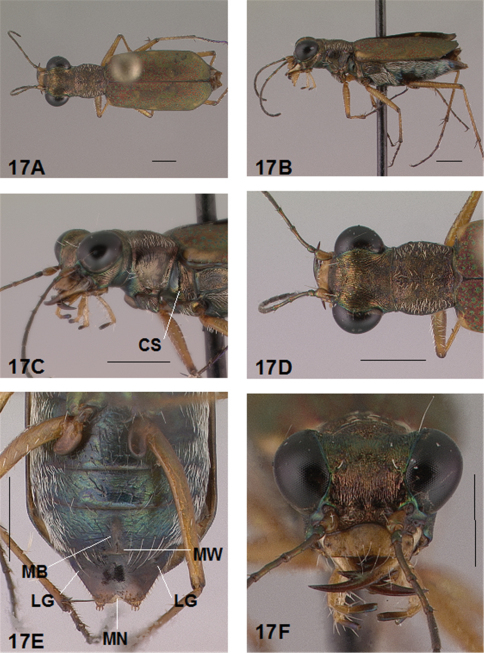
*Brasiella youngi*, sp. n., female. Allotype **17A** Body, dorsal **17B** Body, lateral **17C** Body, anterior, left lateral–CS, coupling sulcus **17D** Body, anterior, dorsal **17E** Abdomen, sterna, ventral–5^th^ sternum, MB, membranous band; 5^th^ sternum, MW, membranous wedge; 6^th^ sternum, LG, lateral gibbosity; 8^th^ sternum, MN, median notch **17F** Head, anterior. [Scale lines = 1 mm].

*General.* Figs 16A, 17A. *Body.* Formslender; head small, eyes proportionally large, prominent and distinctly bulging laterally; pronotum slender, subglobose to subarcuate, wider than long; elytra uniformly narrow, apices separately rounded in female, conjointly rounded in male; appendages primarily testaceous in female, mainly dark metallic in male. *Size*.Male, length 6.0 mm, width 1.7 mm; female, length 6.2 mm, width 1.8 mm.

*Head*. Figs 16B, 16D, 17D, 17F. Shiny dark copper brown dorsally and blue green ventrally; entire surface glabrous except for two pairs of supraorbital sensory setae. Frons finely and longitudinally rugose. Vertex more coarsely rugose, transverse rugae along anterior margin narrow and irregularly arranged, 15-18 more or less complete longitudinal rugae between eyes and middle, in female rugae mainly parallel only slightly converge, in male rugae converge into an arcuate pattern; rugae transition abruptly into a posterior area with a finely and irregularly granulate surface. Eyes prominent and greatly bulging laterally in both sexes. Genae longitudinally rugose. Clypeus finely and irregularly granulate, narrowed mesad. Labrum testaceous with a dark brown margin, rectangular, width to length ratio 3 in holotype male, ratio 3 in allotype female; anterior margin nearly straight with a small tooth; posterior margin distinctly arcuate mesad; medial carina broadly raised; 8 setae in an irregular row near middle symmetrically arranged. Maxillae and labium mainly testaceous, only distal palpal segments dark brown with metallic green black reflections. Mandibles sexually dimorphic; in male, surface mainly testaceous, only teeth metallic green; in female, surface only testaceous in basal half, apical half and teeth shiny brown; mandibles symmetrical, four teeth distad of molar, apical tooth longest, first and third tooth coequal in length, second tooth shortest; gaps between three intermediate teeth narrow in male, wide in female. Antennae 11 segmented; scape in female entirely testaceous, in male dorsally shiny green, ventrally testaceous; scape with a single subapical sensory seta; antennomeres 2-4 shiny copper green, glabrous except for a few, short erect setae along their length and distally; antennomeres 5-11 dull brown, sheathed with dense short sensory setae.

*Prothorax.* Figs 16C, 16D, 17C, 17D. Pronotum shiny, dark copper brown. Proepisterna shiny, dark copper brown, surface wrinkled dorsad. Pronotum glabrous except for short, decumbent, white setae distributed in several, irregular rows medially directed, originating close to, and lying in a narrow band inside and slightly impinging on lateral suture, in a sparse narrow band transversely and anteriorly oriented on anterior margin, and in a sparse narrow band laterally oriented on each side of midline extending nearly to the narrow posterior margin; transverse submarginal sulci distinct, anterior sulcus shallow, posterior sulcus deeper and deepest at posterior angles; transverse rugae within broad anterior margin irregular and shallow, interrupted at middle by an irregularly arranged pattern, within posterior margin more distinctly and deeply engraved especially medially and extending onto midline; surface sculptured by fine, transverse rugae angled on disc and interrupted by a finely engraved longitudinal midline, and more finely and irregularly sculptured elsewhere. Proepisterna glabrous except for white, erect and appressed setae arising from small setigerous punctures scattered over most of the surface in male, only ventrally and along anterior margin in female. Prosternum glabrous, surface slightly roughened.

*Pterothorax.* Figs 16C, 17C. Mesepisterna glabrous except for appressed setae near ventral margin; female mesepisternal coupling sulcus a shallow, concentric depression with a small, central pit, indistinct groove extends only dorsally from pit, surface smooth below pit. Mesepimeron with a few appressed setae. Metepisterna with scattered appressed setae, more abundant in male than female. Prosternum and mesosternum glabrous, smooth to slightly wrinkled; metasternum glabrous except for long, dense white appressed setae laterad, surface smooth mesad and coarsely sculpted laterad where setae originate. Scutellum triangular, cupreous.

*Legs.* Figs 16A, 17B. Appendages in female primarily testaceous and translucent except for slightly darker coxae and tarsomeres; appendages in male dominated by dark metallic reflections. Coxae shiny metallic brown green; tarsomeres shiny violet to green; white, appressed setae on front and middle coxae, and laterally on hind coxae; erect setae and suberect closely spaced in several regular and irregular rows on all femora; setae widely spaced in a few rows on all tibiae; middle tibiae with patch of appressed setae dorsally along distal half; tarsomeres with short scattered setae mainly on ventral surface; distal tarsomeres with two asymmetrical rows each with a few to several small, erect setae; an erect subapical seta present only on front trochanter, absent on middle and hind trochanters; males with dense pad of erect setae ventrally on proximal three tarsal segments; tarsal claws small.

*Elytra.* Figs 16A, 17A. Form uniformly narrow in both sexes; apices separately rounded in female, conjointly rounded in male; sutural spine at apex small and distinct; posterior margins minutely microserrulate. Surface finely granulate, impunctate, numerous small, irregular, shiny green or blue green flecks of various sizes scattered over a dull, dark copper brown background; fully developed elytral pattern of broad, bold markings contrasting with the darker elytral ground color; setigerous punctures with short, erect, transparent setae indistinct in subsutural rows on disc, but distinct at elytral base, and at inner humeral angles, each surrounded by a metallic fleck slightly larger than flecks elsewhere on elytra; surface slightly depressed in humeral area and on disc creating a slight but distinct raised area basally. Elytra dull cupreous ground color with metallic blue and green flecks scattered randomly over the unmarked portions of the surface. The markings faint, nearly translucent elytral markings, forming a complete, but reduced pattern with humeral lunule divided into a humeral spot and a discal dot, a complete middle lunule enlarged near the lateral margin and near the suture, and a complete apical lunule broadly reaching the suture. Elytral epipleura testaceous except for narrow, metallic green to copper green band along dorsal margin.

*Abdomen.* Figs 17B, 17E. Surface of 1st-5th sterna shiny black with green reflections, 6th sternum entirely shiny black to black brown; posterior margins of male 3rd-5th sterna and female 3rd-4th sterna narrowly black; posterior margin female 5th sternum broadly black; 3rd-5th sterna medially smooth with scattered, fine, erect setae in both sexes; male 1st-6th sterna and female 1st-5th sterna laterally mainly covered with dense, scattered, appressed white setae and roughened from setal punctures; male 6th sternum glabrous medially with a broad, deep concave notch; female 5th sternum with moderately raised, transverse wrinkles interrupted by a wide, membranous band along midline extending anteriorly only to middle of sternum from a small, membranous wedge along posterior margin; female 6th sternum entirely glabrous, posterior margin with a row of 6-10 erect spines and a small lateral gibbosity on each side; female 8th sternum median notch shallowly incised.

*Male Genitalia.* Figs 16E, 16F, 16G. Shape narrow near base, uniformly broad in along most of its length, distally narrowed to a short and wide neck, apical hook inner angle acutely rounded and outer angles evenly rounded, tip long and acutely angled to aedeagus. Aedeagus inner sac sclerites: stylet long and straight,, tip pointed; shield unevenly rounded distad; large tooth small and pointed at tip with very long root and large dark fields; arched piece long and thick; spine field within aedeagus neck short and forked.

#### Ecology.

The unique holotype male was collected at 130 m in a flight intercept trap in deciduous forest, whereas the unique allotype female specimen was collected in wet, deciduous forest at 730 m. Deciduous forest typical of the lower slopes is shown in Fig. 20F, whereas deciduous forest typical of the middle slopes is shown in Fig. 20D. Although the collection method for the allotype was not specified on its labels, a passive method of either malaise or yellow pan traps were both employed by CMNH staff while collecting at a site for several days. Both specimens were found on the lower south slopes of the Sierra de Baoruco in September. Vegetation in this part of the Sierra de Baoruco gradually transitions from desert-like habitats below 255 m through dry deciduous forest at 255 m to moist tropical forest and eventually grass and pine habitat at higher elevations (Woodruff 2004). Because of this great diversity of habitats, these mountains at the eastern end of the southernmost part of the Dominican Republic have high floristic and faunal endemism (Woodruff 2004).

#### Distribution.

Fig. 22. DOMINICAN REPUBLIC: Pedernales Province, 13.5 km to 26 km north of Cabo Rojo on the lower southern slopes of the Sierra de Baoruco in deciduous forest habitats at elevations from 130 to 730 m.

#### Etymology.

This Latinized eponym, genitive case, is based on the last name of Chen W. Young, Associate Curator of Invertebrate Zoology, Carnegie Museum of Natural History, Pittsburgh, PA, and world authority on the Diptera family Tipulidae. Chen participated in most of the expeditions by CMNH staff to the Dominican Republic and specifically the one that collected of this new *Brasiella* species. As a friend and colleague, I have the honor of naming this new species for him.

#### Remarks.

*Brasiella youngi* and *Brasiella rawlinsi* appear to be closely related species based on their male genitalia, but their distinctiveness as separate species has been established by differences in the external adult male and female characters presented in the key and under their descriptions. Obvious morphological differences between the two species are exhibited by the size of their eyes and the extent to which the pale elytral pattern is infuscated by the darker ground color. Other important distinctions exist between females of these two species in the depth and position of the female coupling sulcus, the extent to which the membranous, longitudinal median band is developed on the 5th abdominal sternum and the form of the emargination on the 8th sternum. Although both species occur in the Sierra de Baoruco at the same time of year, each species occurs at a different elevation in quite different habitats (refer to Ecology for each of these species). *Brasiella youngi* is less closely related to *Brasiella iviei* based on their male genitalia; although both of these species apparently occur in more moist habitats than *Brasiella rawlinsi*.

**Figure 18.**
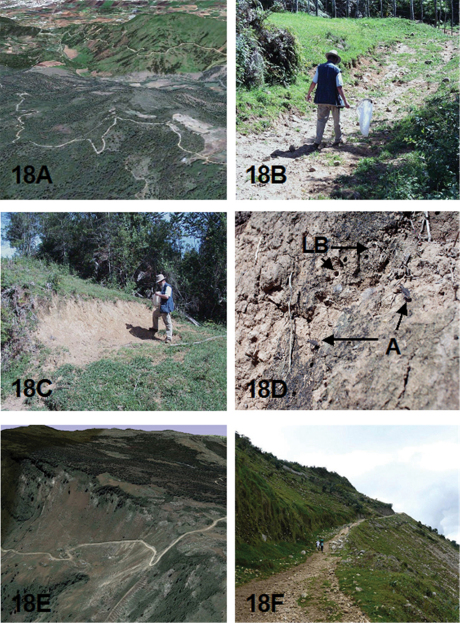
*Brasiella* Habitats on Hispaniola **18A** *Brasiella bellorum*, sp. n., type locality vicinity; aerial view of fields and forests at U-shaped curve in road network near El Convento, Dominican Republic **18B** *Brasiella bellorum*, sp. n., type locality; Robert L. Davidson collecting along eroded pasture road **18C** *Brasiella bellorum*, sp. n., type locality; Robert L. Davidson collecting at eroded clay bank in pasture **18D** *Brasiella bellorum*, sp. n., type locality; breeding site in clay bank with larval burrows (LB) and adults (A) **18E** *Brasiella darlingtoniana*, sp. n., type locality vicinity; aerial view along Highway 101 below La Visite National Park, Massif de la Selle, Haiti **18F** *Brasiella darlingtoniana*, sp. n., type locality; eroded banks and road surface along Highway 101 below La Visite National Park, Massif de la Selle, Haiti.

**Figure 19.**
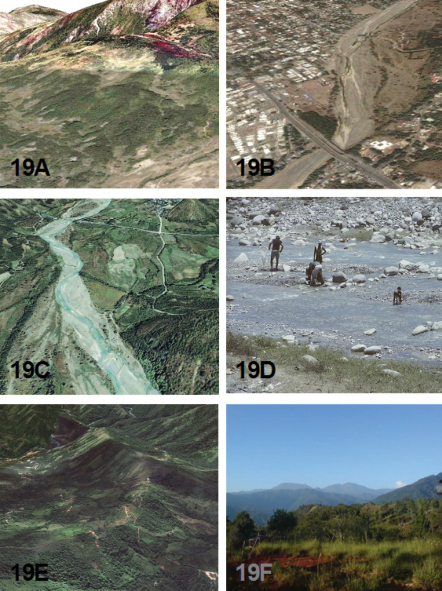
*Brasiella* Habitats on Hispaniola **19A** *Brasiella davidsoni*, sp. n., type locality vicinity; aerial view of roads and fields in disturbed forests at Morne Formon southern slope of the Massif de la Hotte, Haiti **19B** *Brasiella dominicana* (Mandl), type locality vicinity; aerial view of Highway 2 crossing Rio Bani along east side of Bani, Dominican Republic **19C** *Brasiella ocoa*, sp. n., type locality vicinity; aerial view of Rio Ocoa flood plain south of Highway 2 near Las Carreras, Dominican Republic **19D** *Brasiella ocoa*, sp. n., habitat; sparsely vegetated riverbanks along the sand, gravel and rock river bed of Rio Ocoa at Highway 2 crossing near Carreras, Dominican Republic **19E** *Brasiella philipi*, sp. n., type locality vicinity; aerial view of dirt roads among fields and forests at Cierracita above Mata Grande in the northern foothills of the Cordillera Central, Dominican Republic **19F** *Brasiella philipi*, sp. n., type locality vicinity; clay soil openings in field looking south to the Cordillera Central, Dominican Republic.

**Figure 20.**
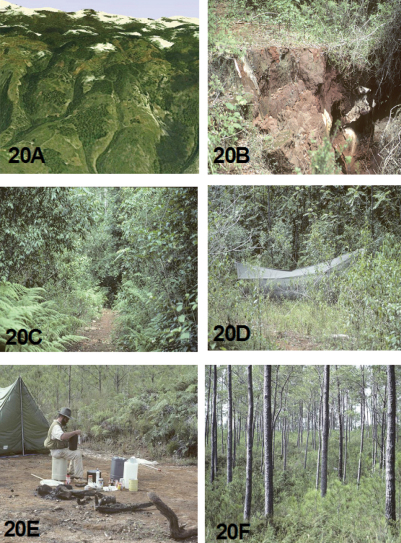
*Brasiella* Habitats on Hispaniola **20A** *Brasiella iviei*, sp. n., type locality vicinity; aerial view of forested ravines similar to the Las Abejas type locality, mid-elevations, southern slopes of the Sierra de Baoruco, Dominican Republic **20B** *Brasiella iviei*, sp. n., habitat; eroded, clay banks of watercourse along trail at upper parts of Las Abejas, south side of the Sierra de Baoruco, Dominican Republic **20C** *Brasiella iviei*, sp. n., habitat; trail entering upper parts of Las Abejas, southern slopes of the Sierra de Baoruco, Dominican Republic **20D** *Brasiella iviei*, sp. n., type locality vicinity; tropical malaise trap at lower parts of Las Abejas set by 1987 CMNH Expedition, southern slopes of the Sierra de Baoruco, Dominican Republic **20E** *Brasiella rawlinsi*, sp. n., type locality vicinity; Robert L. Davidson at 1987 CMNH Expedition campsite in compacted clay opening within an old burn regenerating with shrubby regeneration and young *Pinus occidentalis* Swartz in background, upper elevations, southern slopes of the Sierra de Baoruco, Dominican Republic **20F** *Brasiella rawlinsi*, sp. n., type locality vicinity; Aceitillar with *Pinus occidentalis* Swartz forest at upper elevations, southern slopes of the Sierra de Baoruco, Dominican Republic.Dominican Republic.

**Figure 21.**
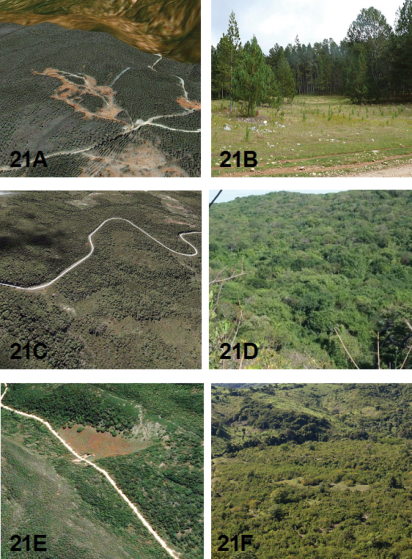
*Brasiella* Habitats on Hispaniola **21A** *Brasiella rawlinsi*, sp. n., type locality vicinity; aerial view of Aceitillar, *Andropogon* sp. grassland with *Pinus occidentalis* Swartz and bauxite surface mines at upper elevations, southern slopes of the Sierra de Baoruco, Dominican Republic **21B** *Brasiella rawlinsi*, sp. n., habitat; Aceitillar*, Andropogon* sp. grassland openings in young *Pinus occidentalis* Swartz on red clay soils, southern slopes of the Sierra de Baoruco, Dominican Republic **21C** *Brasiella youngi*, sp. n., type locality vicinity; aerial view of wet, deciduous forest near the Haitian Hut along Alcoa Road at mid-elevations on the southern slopes of the Sierra de Baoruco, Dominican Republic **21D** *Brasiella youngi*, sp. n., habitat: wet deciduous forest typical of allotype site of this species was collected **21E** *Brasiella youngi*, sp. n., type locality vicinity; aerial view of deciduous woodlands adjoining desert scrub typical of holotype site at lower elevations on the southern slopes of the Sierra de Baoruco, Dominican Republic **21F** *Brasiella youngi*, sp. n., habitat; dry, deciduous woodlands typical of holotype site on the lower slopes of the Sierra de Baoruco, Dominican Republic.

**Figure 22.**
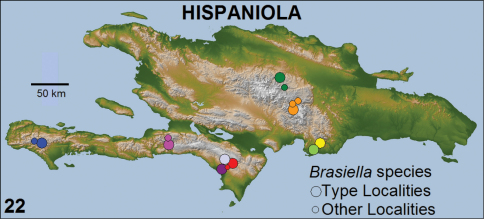
Geographic Distribution of *Brasiella* species on Hispaniola. The type locality, other localities, country, province or département and geographic feature associated with each species: (ORANGE) *Brasiella bellorum*, sp. n., DOMINICAN REPUBLIC: La Vega Province, El Convento, Cordillera Central; (VIOLET) *Brasiella darlingtoniana*, sp. n., HAITI: Département du l'Ouest, La Visite, Massif de la Selle; (BLUE) *Brasiella davidsoni*, sp. n., HAITI: Département du l'Sud, Ville Formon, Massif de la Hotte; (YELLOW) *Brasiella dominicana* (Mandl), DOMINICAN REPUBLIC: Peravia Province, Bani, Rio Bani; (GRAY) *Brasiella iviei*, sp. n., DOMINICAN REPUBLIC: Pedernales Province, Las Abejas, Sierra de Baoruco; (LIME GREEN) *Brasiella ocoa*, sp. n., DOMINICAN REPUBLIC: Peravia Province, Las Carreras, Rio Ocoa; (GREEN) *Brasiella philipi*, sp. n., DOMINICAN REPUBLIC: Santiago Province, La Sierrecita, Cordillera Central; (RED) *Brasiella rawlinsi*, sp. n., DOMINICAN REPUBLIC: Pedernales Province, Aceitillar, Sierra de Baoruco; (PURPLE) *Brasiella youngi*, sp. n., DOMINICAN REPUBLIC: Pedernales Province, 13.5 km N Cabo Rojo, Sierra de Baoruco.

##### Relationships within the *Brasiella virdicollis* Species Group of *Brasiella* Rivalier

Although a phylogenetic analysis of *Brasiella* species on Hispaniola is not part of the present revision, a general discussion of the latest published phylogenetic treatment of *Brasiella* relative to the Hispaniola species, is considered relevant. Emphasis is placed on the composition of the *Brasiella virdicollis* species group, along with its current and ancestral geographic distributions in the West Indies. Such a discussion is considered relevant for suggesting further studies to investigate the phylogenetic relationships for the Hispaniola *Brasiella* species treated in this revision relative to other Western Hemisphere *Brasiella*.

Previous phylogenetic and biogeographic studies of tiger beetle genera in Brazil (Freitag and Barnes 1989) and West Indies (Freitag 1992) recognized that *Brasiella sensu* Rivalier possessed a unique suite of synapomorphous adult characters not found in related Western Hemisphere taxa of Cicindelinae. However, their studies treated *Brasiella* Rivalier 1954 subspecifically within *Cicindela* Linnaeus 1758, a concept held by many North American tiger beetle students, but not by those studying either elsewhere in the world or the higher classifications of Coleoptera. Consequently, in the most recent and comprehensive treatments of the world Geadephaga (Lorenz 2005) and Western Hemisphere Caraboidea (Erwin and Pearson 2008), *Brasiella* was recognized as distinct among Cicindelina Latreille 1802 lineages and given full generic status. Although treated as a distinctive genus, the latter authors considered the taxonomic status of species within *Brasiella* to be unstable, likely because further discovery of new species would require changes to the current classification. As the present revision demonstrates, the Neotropical *Brasiella* fauna is likely to be far richer than indicated by current faunal lists, especially after habitats have been more fully explored in both new and previously visited geographic areas throughout all seasons. Additionally, more detailed studies of genitalic structures, among populations of those species already described, may reveal cryptic species, as the present revision has shown to be the situation on Hispaniola.

Indeed, the discovery and description of new species periodically provides the opportunity to reconsider any existing published phylogeny so that the relationships of the new species to those previously described are better understood. One approach would be to use the criteria of the existing classification and simply fit the new species into it. This first approach seems appropriate when a small number of new species are considered from a restricted geographic area where populations have been adequately sampled such that the faunal diversity is considered to be entirely known. Another approach would be a more comprehensive reevaluation of the criteria of the existing classification in order to develop a revised classification. This second approach would be most appropriate when considering a large number of new species originating over broadly diverse geographic areas. Under this second approach, results would be most informative when populations from all these geographic areas have been adequately sampled.

Thus, for either approach the question of adequately sampling existing populations is critical to any meaningful reconstructed phylogeny. Because the present revision revealed new *Brasiella* species with each expedition to Hispaniola, there is a high probability that many additional new species will be discovered on this island. Further collecting is likely to find these, not only within new areas and habitats, but also within those previously visited, especially during different seasons on Hispaniola.

Even though the *Brasiella* populations on Hispaniola may not have been completely sampled, this present revision did provide an opportunity for subjective interpretations of the species relationships within this genus on Hispaniola based on the population samples that were collected thus far and studied by the author. These relationships were discussed earlier in this revision under the Remarks within each Species Account. Such interpretations, while based on the author's observations and experience with the species, should provide a working hypothesis for any future phylogenetic analysis designed to examine more rigorously the relationships among the Hispaniolan *Brasiella* species, all of which are endemic to this island and superficially appear similar to one another.

The current classification into species groups of nearly all *Brasiella* described at the time was presented by Freitag and Barnes (1989). *Brasiella dominicana* (Mandl), described in 1983, unexplainably was overlooked. However, this species later was studied and subsequently assigned to a species group within *Brasiella* by Freitag (1992). In their publication, Freitag and Barnes (1989) established seven species groups for 29 species within the genus based on a reconstructed phylogeny involving 50 adult morphological characters. Despite the fact that male genitalic characters of most species were not actually examined and documented by dissection, but rather interpreted only from the published descriptions and illustrations, their results were important because they provided a basis for all subsequent efforts to phylogenetically organize the ever-growing number of *Brasiella* species found in the Western Hemisphere. The latest catalogue (Erwin and Pearson 2008) treated 39 *Brasiella* species, and with the eight more added in the present revision alone, *Brasiella* is now at 47 species.

Although Freitag and Barnes (1989) established morphological characters and assigned their polarities--plesiomorphic (ancestral) or apomorphic (derived)--for all 50 adult characters, male and female genitalic characters were given the most value in establishing the species groups within *Brasiella* phylogeny. While they treated primarily Brazilian species in their publication, they did establish the *viridicollis* species group for several *Brasiella* species occurring in the West Indies together with one species from North America. However, this species group was not discussed in detail until Freitag (1992) studied the West Indian Cicindelidae. In that publication, he reassigned two *Brasiella* species he had originally placed within the *viridicollis* species group, and added one species he had previously overlooked, so that the species group at that time included the following three species--*Brasiella viridicollis* (Dejean 1831) from Cuba, *Brasiella dominicana* (Mandl) from Hispaniola, and *Brasiella wickhami* (W. Horn 1903) from northern Mexico and the southwestern United States.

The numerous new species described in the present revision have made it necessary to redefine the earlier concept of the *Brasiella viridicollis* species group. Decisions to include newly described species, or exclude those previously described, would result from applying the revised concept. The present revision suggests that the *Brasiella viridicollis* species group concept would be strengthened if a previously overlooked adult female structure, a small gibbosity located on each side of the 6^th^ abdominal sternum, was included in its concept. This structure, and two membranous structures on the 5^th^ abdominal sternum, are considered to be apomorphic. The synapomorphic gibbosity character for females of nearly all Hispaniolan *Brasiella*, as well as, the Cuban *Brasiella virdicollis* (Dejean). provides further evidence for treating the *viridicollis* species group as an evolutionary unit within the *Brasiella*. All three structures appear relevant to the *Brasiella viridicollis* species group concept and are discussed in detail here.

Freitag and Barnes (1989) were the first to report a membraneous structure along the posterior margin of the female 5^th^ sternum. They considered this unusual female abdominal character unique within Western Hemisphere Cicindelinae and termed it an “unpigmented bell-shaped spot”. However, this structure is actually membranous as explained above under Methods; thus, in the present revision it has been termed a “membranous wedge.” The second membranous structure, also on the same female 5^th^ abdominal sternum, is a longitudinal “membranous band”, mesad and anterior to this membranous wedge. The membranous band does not seem to have been considered a separate structure by Freitag and Barnes (1989). These two structures exist for nearly all *Brasiella* species on Hispaniola. The exceptions being *Brasiella ocoa* whose females are unknown, and *Brasiella davidsoni* whose females plausibly have secondarily lost the 5^th^ abdominal sternum structures.

The third structure is the small, raised gibbosity on each side of the female 6^th^ abdominal sternum located mesad on each surface. These two gibbosities have not been previously mentioned in any publication dealing with *Brasiella* phylogeny (Freitag and Barnes 1989)(Freitag 1992). As mentioned above, these abdominal gibbosities are considered an important apomorphic character for including or excluding species within the established *Brasiella* species groups. The gibbosity character is synapomorphic for females of eight of the nine *Brasiella* species on Hispaniola; *Brasiella ocoa* is known only from males, but its females are likely to possess this character.

Those species in the *Brasiella viridicollis* species group that do not occur on Hispaniola require consideration. Apparently, two subspecies of *Brasiella virdicollis* now have been recorded on Cuba; the nominotypic subspecies and *Brasiella viridicollis fernandozayasi* (Kippenhan, Ivie & Hopp 2009). *Cicindela fernandozayasi* was proposed as a replacement name (Kippenhan et al. 2009) for *Cicindela carbonaria* Zayas 1988 (Zayas 1988). Zayas (1988) described his species from a single specimen without any detail as to where it was collected on Cuba. This taxon currently is classified as *Cicindela viridicollis fernandozayasi* (Kippenhan, Ivie & Hopp 2009), but treated as a *Brasiella* in this revision. This subspecies has a completely black dorsal body color compared to nominotypic *Brasiella viridicollis* with its distinctive blue green head and pronotum contrasting with dull brown elytra covered by metallic blue green flecks.

Nominotypic *Brasiella virdicollis* (Dejean) possesses all three of these apomorphic structures on the two last visible sterna based on the author's study of female specimens from Cuba, Cienfuegos Province, Minas Carlota, Sierra de Trinidad, at CMNH. The same cannot be said for the second subspecies because no specimens of *Brasiella viridicollis fernandozayasi* were available for examination. This subspecies is known only from its holotype deposited in the Fernando Zayas Collection in Havana, Cuba (Kippenhan et al. 2009). There seems little doubt about its placement within the *Brasiella viridicollis* species group, however, its subspecific status may need to be reconsidered. The color image of the type presented by Kippenhan et al. (2009) exhibits some morphological characters different from the nominotypic *Brasiella viridicollis* that suggest it may represent a separate species. Although the sex of the holotype of *Brasiella viridicollis fernandozayasi* was not indicated when it was described as *Cicindela carbonaria* Zayas 1988, nor when it received its current replacement name, it appears to be a male. The conclusion is based on the expanded setal pads on the proximal three tarsomeres, the shape of its elytral apices and the protruding abdomen from the illustration and image in these two publications. When compared with a male of *Brasiella viridicollis*, the most obvious difference between these two subspecies, other than their strikingly different dorsal body color, lies in the large, laterally bulging eyes of *Brasiella viridicollis fernandozayasi* compared with the smaller, less bulging eyes of the nominotypic *Brasiella viridicollis*. Final resolution about the specific status of each subspecies must await additional examination of their types, as well as, the discovery of more male specimens of *Brasiella viridicollis fernandozayasi* whose genitalia can be dissected.

Evidence is presented here that *Brasiella wickhami* (W. Horn) should not be considered part of the *Brasiella viridicollis* species group. This species has been included within the *Brasiella viridicollis* species group (Freitag and Barnes 1989, Freitag 1992) on the basis of its aedeagus having a shield rounded at the apex and slightly protruding from the male genitalia. However, the shield for *Brasiella wickhami* is actually a more complex sclerite than the simple shield possessed by the other species within the *Brasiella viridicollis* species group. Rivalier (1955) depicted the shield of *Brasiella wickhami* as a large sclerite with both a rounded knob and a pointed section at the apex. My examination of specimens from Arizona at CMNH, confirm this more complex shield shape. Moreover, *Brasiella wickhami* females lack the lateral gibbosities on the 6^th^ abdominal sternum. From these findings, it is suggested that *Brasiella wickhami* should not be a part of the *Brasiella* *viridicollis* species group. The assignment of *Brasiella wickhami* to another species group within *Brasiella* must await a phylogenetic analysis of the other mainland species in the Neotropics of North and Central America.

As the previous discussion suggests, the revised *Brasiella viridicollis* species group concept includes only the following ten species and two subspecies arranged here alphabetically by country and species within each country:

*Brasiella viridicollis fernandozayasi* (Kippenhan, Ivie & Hopp) – Cuba

*Brasiella viridicollis viridicollis* (Dejean) – Cuba

*Brasiella bellorum*, new species, Acciavatti – Dominican Republic

*Brasiella dominicana* (Mandl) – Dominican Republic

*Brasiella iviei*, new species, Acciavatti – Dominican Republic

*Brasiella ocoa*, new species, Acciavatti – Dominican Republic

*Brasiella philipi*, new species, Acciavatti – Dominican Republic

*Brasiella rawlinsi*, new species, Acciavatti – Dominican Republic

*Brasiella youngi*, new species, Acciavatti – Dominican Republic

*Brasiella darlingtoniana*, new species, Acciavatti – Haiti

*Brasiella davidsoni*, new species, Acciavatti – Haiti

How these current species distributions within the *Brasiella viridicollis* species group may have been influenced by their ancestral geographic distributions should be briefly considered. Previous biogeographic studies of tiger beetle genera in the West Indies provided only a partial explanation of the possible pathways by which the *Brasiella viridicollis* species group may have arrived on Cuba and Hispaniola from its ancestral distributions. According to Freitag (1992), the dispersal pathway accounted for the ancestors of the *Brasiella* on Cuba and Hispaniola. He suggested that these *Brasiella* ancestors where originally distributed in Middle America and arrived on these islands most likely through biogeographical patterns of dispersal common to tiger beetles. He did not distinguish between active and passive dispersal mechanisms for the large, actively flying *Cicindela* species compared with the small, primarily cursorial *Brasiella* species. Dispersal as a pathway implied movement of species from ancestral distributions in the Yucatan Peninsula or Nicaragua Highlands to the Greater Antilles over wide water gaps. Such dispersal might be rare, but likely has occurred over time even for small *Brasiella* species, as documented recently for *Brasiella viridicollis* from Cuba to the Florida Keys(Schiefer 2004).

Although the dispersal pathway undoubtedly has influenced the currrent distribution of the *Brasiella viridicollis* species group, the vicariance pathway also should be considered in explaining current geographic distributions. Woodruff (2004) provided insight into the likely influence of both dispersal and vicariance on the current geographic distributions for *Phyllophaga* species (Coleoptera: Scarabaeidae: Melolonthinae) on Hispaniola. He summarized the concepts about the geologic history of Hispaniola prevalent at the time of his revision.

*Brasiella* species divergence on Hispaniola apparently occurred after the arrival of the ancestral members of the *Brasiella viridicollis* species group. Species divergence likely was greatly influenced by the extent and duration to which populations were isolated, especially during the Pleistocene. During interglacial episodes in that geologic time period, the Caribbean Sea would have risen from melting continental glaciers and inundated the lowlands. Lowland inundation over a long time could have kept populations isolated the longest in the highest and most widely separated mountain masses, such as the Massif de la Hotte in southwestern Haiti, where the most divergent *Brasiella davidsoni* presently exists on Hispaniola. All these biogeographic concepts discussed here should be considered, and discussed in greater detail, when interpreting future phylogenetic studies as to how the *Brasiella* species on Hispaniola relate to each other, as well as, to the other *Brasiella* species in the Neotropics.

## Conclusions

In the latest and most comprehensive taxonomic treatises of Caraboidea, *Brasiella* is established as a distinct genus which is confirmed by a suite of synapomorphic characters. Earlier taxonomists, such as Walther Horn, who examined the *Brasiella* specimens first collected by Philip Darlington in Haiti, assigned all the *Brasiella* on Hispaniola to the widespread Neotropical *Brasiella argentata* (Fabricius). More recent taxonomists, such as Richard Freitag, considered all the *Brasiella* specimens on Hispaniola to be the insular endemic *Brasiella dominicana* (Mandl). The current revision of the *Brasiella* tiger beetle fauna on Hispaniola suggests a greater species diversity exists on this island than previously recognized.

From a comparison of the holotype and paratypes of *Brasiella dominicana* (Mandl) with other *Brasiella* taken on Hispaniola, it was concluded that this species is restricted to a small area likely along the Rio Bani where it flows from the eastern end of the mountainous Cordillera Central onto the southern coastal plain in the Dominican Republic. A closely related species, *Brasiella ocoa*, new species, also occurs allopatrically at low elevations west of this area along the Rio Ocoa near the coast. At high elevations in the Cordillera Central are found two other allopatric species: *Brasiella bellorum*, new species, from the central portions, and *Brasiella philipi*, new species, from the north slopes of this mountain range, respectively. Compared with *Brasiella dominicana, Brasiella ocoa* appears to be more closely related, whereas, *Brasiella bellorum* and *Brasiella philipi*,are more distantly related species.

Three endemic species isolated in the Sierra de Baoruco in southern Dominican Republic, appear to be sympatric, but occupy different ecological habitats along an altitudinal gradient. *Brasiella iviei*, new species, the largest species in Hispaniola, occurs in moist, mixed deciduous forests at upper elevations. *Brasiella rawlinsi*, new species, occurs in drier grass and pine dominated habitats at higher elevations, whereas, *Brasiella youngi*, new species, occurs in wet, deciduous forested habitats at lower elevations. All three of these species have individuals whose elytral markings are faint or completely obliterated by the darker background color with shiny iridescent flecks. In this regard, these three species appear to be more closely related to each other than to the other *Brasiella* species on Hispaniola.

The *Brasiella* in neighboring Haiti presently are known to occur only in the highest mountains of that country. *Brasiella darlingtoniana*, new species, from the Massif de la Selle, Haiti, and the most divergent species, *Brasiella davidsoni*, new species, from the Massif de la Hotte, Haiti, appear to be more closely related to each other than to the other *Brasiella* species on Hispaniola.

All nine *Brasiella* species on Hispaniola belong to the *viridicollis* species group within the earlier subgeneric concept of *Cicindela* (*Brasiella*) as established by Freitag and Barnes (1989) and Freitag (1992) from their phylogenetic studies of Brazilian and West Indian tiger beetles. *Brasiella viridicollis viridicollis* (Dejean), and *Brasiella viridicollis fernandozayasi* (Kippenhan, Ivie & Hopp) occur on Cuba. Evidence is presented for considering *Brasiella viridicollis fernandozayasi* (Kippenhan, Ivie & Hopp) may represent a distinct species within the species group, and for removing *Brasiella wickhami* (W. Horn) from this species group.

As elucidated by Freitag (1992), ancestors of the *Brasiella viridicollis* species group on Hispaniola and Cuba would have arrived there most likely through biogeographical patterns of dispersal common within the Greater Antilles from ancestral distributions in Middle America through the Yucatan Peninsula or Nicaragua Highlands. The degree to which *Brasiella* species diverged after arrival on Hispaniola apparently has been greatly influenced by the extent and duration of their isolation during Pleistocene interglacial periods when the Caribbean Sea filled the lowlands. Lowland inundation for long periods of time would likely have isolated populations in separate mountain masses on Hispaniola, thereby fostering species divergence. The most widely separated mountain masses, such as the Massif de la Hotte in southwestern Haiti, in fact, have the most divergent species, *Brasiella davidsoni*,presently known on Hispaniola. All the biogeographic concepts of dispersal and vicariance should be discussed in greater detail when interpreting future phylogenetic studies as to how the *Brasiella* species on Hispaniola relate to each other and to those in the Neotropics.

## Supplementary Material

XML Treatment for Brasiella bellorum

XML Treatment for Brasiella darlingtoniana

XML Treatment for Brasiella davidsoni

XML Treatment for Brasiella dominicana

XML Treatment for Brasiella iviei

XML Treatment for Brasiella ocoa

XML Treatment for Brasiella philipi

XML Treatment for Brasiella rawlinsi

XML Treatment for Brasiella youngi
